# A Damage‐Gated Small‐Molecule Chimera Selectively Enhances Mitophagy by Coupling Dysfunctional Mitochondria to Autophagy

**DOI:** 10.1002/advs.78570

**Published:** 2026-10-29

**Authors:** Ratislav M. Ozhiganov, Ekaterina А. Guseva, Alexander Yu. Rudenko, Konstantin G. Lyamzaev, Sofya Y. Sokolskaya, Julia A. Golubeva, Maria V. Belopolskaya, Nadezda V. Andrianova, Olga V. Fadeeva, Elena О. Morgun, Elena A. Tukhovskaya, Marina S. Kazakova, Vladimir M. Pavlov, Daria K. Yagoda, Elena I. Marusich, Rose‐Lys Zaranaina, Valeriya B. Vays, Irina M. Vangeli, Egor Y. Plotnikov, Maxim L. Lovat, Lora E. Bakeeva, Arkady N. Murashev, Vladimir I. Polshakov, Olga A. Dontsova, Peter V. Sergiev

**Affiliations:** ^1^ Belozersky Institute of Physico‐Chemical Biology Lomonosov Moscow State University Moscow Russia; ^2^ Chemical Department Lomonosov Moscow State University Moscow Russia; ^3^ Faculty of Fundamental Medicine M.V. Lomonosov Moscow State University Moscow Russia; ^4^ Institute of Mitoengineering Lomonosov Moscow State University Moscow Russia; ^5^ Pirogov Russian National Research Medical University Moscow Russia; ^6^ Shemyakin‐Ovchinnicov Institute of Bioorganic Chemistry (branch) Russian Academy of Sciences Pushchino Russia; ^7^ Faculty of Bioengineering and Bioinformatics Lomonosov Moscow State University Moscow Russia; ^8^ Moscow Center for Advanced Studies Moscow Russia; ^9^ Peoples' Friendship University of Russia named After Patrice Lumumba Moscow Russia; ^10^ Biological Faculty Lomonosov Moscow State University Moscow Russia

**Keywords:** autophagy, bifunctional molecules, damage‐responsive autophagy, drug discovery, medicinal chemistry, mitophagy, neurodegeneration, small‐molecule chimeras

## Abstract

Mitophagy is a central mitochondrial quality‐control pathway, and its pharmacological activation is considered to be geroprotective. However, its small‐molecule activators act indirectly by perturbing metabolism or mitochondrial function, limiting selectivity and increasing toxicity. Here, we describe a chemical biology strategy for activating damage‐responsive mitophagy using bifunctional small molecules that couple mitochondrial recognition to the recruitment of the autophagic machinery. We designed and synthesized a focused library of chimeric molecules and identified MG‐1 as a lead compound. It had minimal effects on basal autophagy but markedly enhanced autophagic flux and mitochondrial clearance under conditions of mitochondrial stress. Biochemical analyses, together with fluorescence and electron microscopy, showed that MG‐1 promotes lysosomal degradation of damaged mitochondria without acting as a direct mitochondrial uncoupler. MG‐1 was well tolerated across experimental systems and improved locomotor performance in a zebrafish model of MPTP‐induced mitochondrial dysfunction. These findings establish damage‐gated small‐molecule chimeras as a promising approach for selective control of mitochondrial quality.

## Graphical abstract

MG‐1 is a bifunctional small molecule that enhances the removal of damaged mitochondria through autophagy, the cell's recycling pathway. Its activity increases under mitochondrial stress, with minimal effects on basal autophagy. MG‐1 also improves movement in a zebrafish model of mitochondrial dysfunction, supporting further investigation of this approach to mitochondrial quality control.

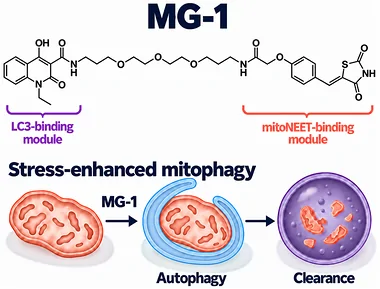

## 1 Introduction

Autophagy is a fundamental catabolic process that preserves cellular homeostasis by mediating the lysosomal degradation of misfolded proteins, damaged organelles, and other cytoplasmic components [1, 2, 3, 4]. At the molecular level, autophagy proceeds through the formation of double‐membrane autophagosomes that engulf portions of the cytoplasm and subsequently fuse with lysosomes, thereby enabling the breakdown and recycling of cellular constituents [3, 4, 5]. Functionally, autophagy serves as a central quality‐control mechanism by eliminating dysfunctional mitochondria and protein aggregates [5, 6], recycling biomolecules to sustain metabolism under nutrient stress [1], and maintaining tissue integrity through regulation of immune responses, cellular differentiation, and stem cell renewal [7].

Disruption of autophagic flux perturbs this finely balanced system and contributes to a broad spectrum of pathological processes [8, 9]. Insufficient autophagy leads to the accumulation of damaged proteins and organelles, resulting in oxidative stress, metabolic imbalance, and ultimately cell death [10]. Defects in mitochondrial quality control are particularly detrimental in the nervous system, where post‐mitotic neurons rely heavily on efficient mitochondrial function to meet high energetic demands. In neurodegenerative disorders such as Parkinson's disease, impaired mitophagy has been directly linked to progressive neuronal loss [11], as damaged mitochondria generate excessive reactive oxygen species, compromise energy metabolism, and promote cellular dysfunction [12].

Mitophagy, a selective form of autophagy, ensures the recognition, sequestration, and lysosomal degradation of dysfunctional mitochondria, thereby preserving mitochondrial integrity and overall cellular homeostasis [4, 6, 13]. Beyond its role in stress adaptation, mitophagy contributes to physiological regulation of mitochondrial number and function during cellular differentiation and metabolic remodeling [14] and interlaces closely with cell survival and death pathways by eliminating mitochondria that could otherwise trigger apoptosis or necrosis [15]. Accordingly, impaired mitophagy has been implicated in diverse pathological contexts, including neurodegenerative diseases, cardiovascular dysfunction, fibrotic disorders, and aging‐related decline [16, 17]. Recent evidence further indicates that suppression of basal mitophagy can actively promote cellular senescence programs in proliferating cells [18].

Given the central role of mitophagy in mitochondrial quality control and cellular health, pharmacological enhancement of this pathway has emerged as an attractive therapeutic strategy. Numerous small‐molecule mitophagy activators, including metabolic modulators, redox regulators, mitochondrial uncouplers, and natural compounds, have been reported to stimulate mitochondrial clearance [19, 20]. Although these agents show promise in conditions associated with mitochondrial dysfunction, such as aging, neurodegeneration, and metabolic disorders [21], many act by directly perturbing mitochondrial function or cellular metabolism, often resulting in limited selectivity and intrinsic toxicity.

At the same time, bifunctional small molecules have recently emerged as a powerful strategy for selective intracellular degradation [22]. These compounds typically comprise two functional modules: one that binds a specific protein or organelle, and another that recruits components of the cellular degradation machinery, such as E3 ubiquitin ligases or the autophagy receptor LC3 [23]. By promoting the formation of ternary or higher‐order complexes, such chimeric molecules enable targeted elimination of defined cellular substrates [23, 24]. Notably, autophagy‐targeting chimeric molecules (AUTACs) contain degradation tags that imitate S‐guanylation to induce K63‐linked polyubiquitination, recognition by autophagy receptors including p62/SQSTM1, and subsequent lysosomal delivery [25]. More recently, autophagosome‐tethering compounds (ATTECs), which simultaneously bind a protein of interest and LC3, have been developed to directly recruit a cargo into autophagosomes for lysosomal degradation [23, 24, 26]. Of particular relevance, Tan et al. recently reported the mitochondria‐directed ATTEC mT1, in which a 2‐phenylindole‐derived TSPO ligand was linked to the LC3B‐binding ligand GW5074. mT1 promoted the autophagic clearance of damaged mitochondria and attenuated disease‐associated phenotypes in cellular and organoid models, without pronounced cytotoxicity under the conditions examined [27].

Building on these advances, a chimera‐based strategy may be leveraged to selectively enhance mitophagy by functionally coupling damaged mitochondria to the autophagic machinery. In the present work, we designed and synthesized a series of hybrid small molecules, including the lead compound **MG‐1**, which integrates a mitochondrial‐targeting ligand with an LC3‐interacting moiety. Rather than acting as a constitutive autophagy inducer, this approach is intended to promote mitochondrial clearance preferentially under conditions of mitochondrial dysfunction.

The aim of this study was to develop and evaluate chimera‐like small molecules that selectively enhance mitophagy in a damage‐dependent manner and to assess their ability to promote the autophagic removal of dysfunctional mitochondria while minimizing nonspecific cytotoxic effects.

## 2 Results

### 2.1 Design and Synthesis of Hybrid Mitophagy‐Directing Molecules

Selective mitophagy can, in principle, be achieved by functionally coupling damaged mitochondria to the autophagic machinery. Guided by this concept, we designed a series of hybrid small molecules that integrate a mitochondria‐interacting module with an autophagosome‐recruiting fragment, thereby enabling chimera‐like behavior at the cellular level.

As an initial mitochondrial target, we selected mitoNEET (CISD1) for several complementary reasons. MitoNEET is anchored in the outer mitochondrial membrane by a single N‐terminal transmembrane helix, whereas its [2Fe–2S]‐containing domain is exposed to the cytosol. This topology makes the protein accessible to a soluble bifunctional molecule and potentially enables recruitment of the cytosolic autophagic machinery. Moreover, mitoNEET participates in the regulation of mitochondrial iron and redox homeostasis, oxidative capacity, and mitochondrial network organization [28, 29, 30, 31], processes which are sensitive to mitochondrial dysfunction.

MitoNEET is also an experimentally established ligandable protein. It was originally identified through saturable binding of radiolabeled pioglitazone and specific photoaffinity cross‐linking with a thiazolidinedione probe [32]. Subsequent structure‐guided studies identified additional thiazolidinedione‐based mitoNEET ligands, including NL‐1 [32, 33]. Accordingly, we selected a benzylidene–thiazolidinedione fragment, structurally related to this validated ligand class, as the mitochondrial‐recognition module. A glycolic acid residue was introduced at the para position of the phenyl ring to provide a chemically accessible handle for linker attachment. Notably, despite extensive investigation of thiazolidinedione derivatives as mitoNEET ligands, their application as targeting modules within chimeric molecular architectures has not, to our knowledge, been reported.

As an alternative mitochondria‐interacting target, we selected the translocator protein (TSPO), which is localized to the outer mitochondrial membrane [34]. TSPO is implicated in a broad range of cellular processes, including apoptosis, proliferation, respiration, immunomodulation, mitochondrial cholesterol transport, and steroid hormone biosynthesis [35, 36]. Importantly, elevated TSPO expression has been reported in activated microglia associated with neurodegenerative and neuroinflammatory disorders such as Alzheimer's disease, Huntington's disease, and multiple sclerosis [35, 37, 38]. For this targeting module, we employed a 4‐phenylquinazoline scaffold (Figure 1), as derivatives of this chemotype have been reported to function as high‐affinity TSPO‐targeting motifs in the development of fluorescent probes [39].

**FIGURE 1:**
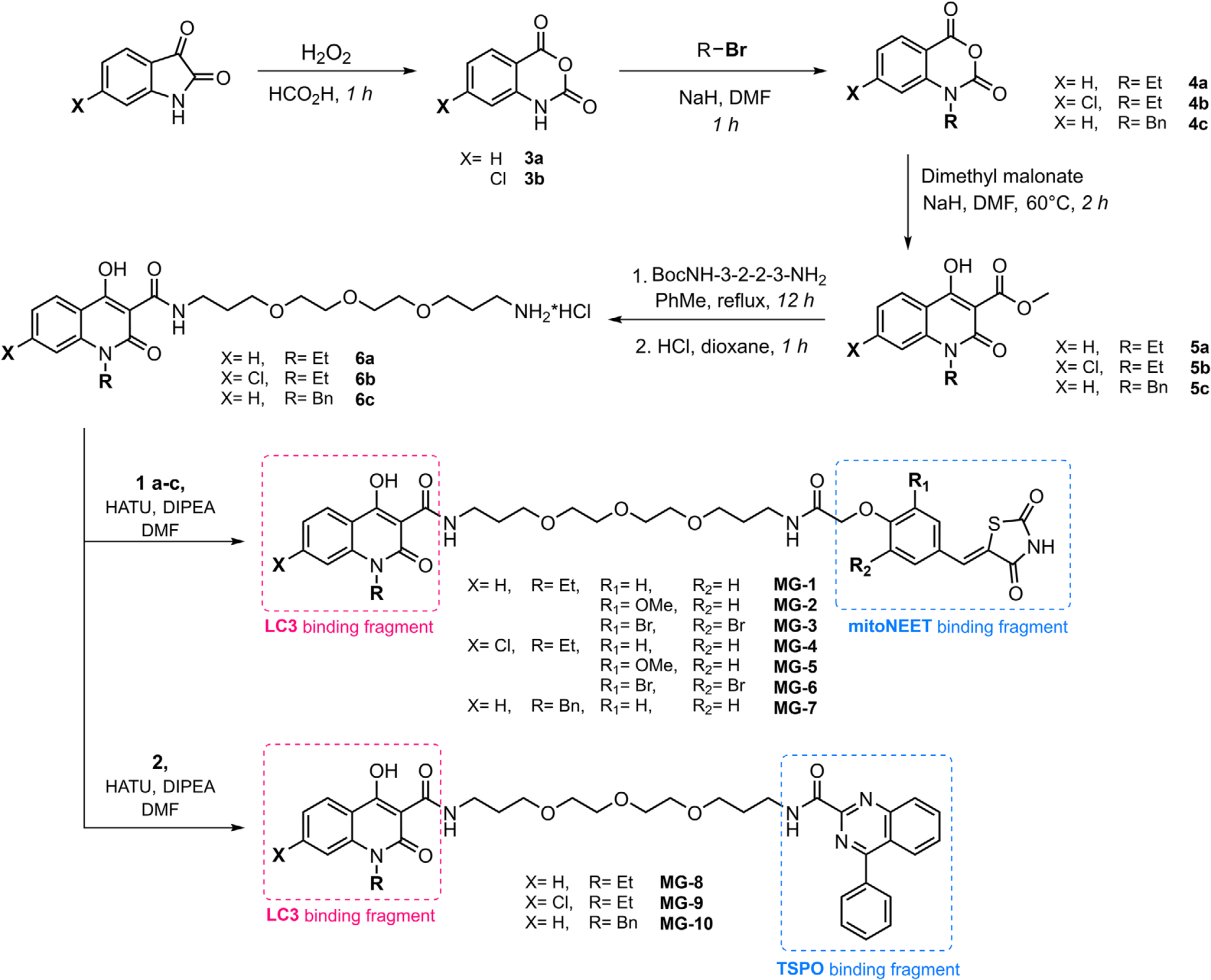
Synthesis of hybrid molecules **MG‐1‐10**. Synthetic route to the hybrid compounds MG‐1–MG‐10 combining an LC3‐binding fragment with either a mitoNEET‐binding (**MG‐1‐7**) or a TSPO‐binding (**MG‐8‐10**) mitochondrial targeting moiety via a 4,7,10‐trioxa‐1,13‐tridecanediamine linker.

The design of the autophagosome‐recruiting moiety was based on a phenotypic screening study reported in 2021 that identified small molecules capable of inducing mitophagy [40]. In that work, the compound VP07 robustly activated mitophagy, and its activity was proposed to arise from LC3 engagement and association with the mitochondrial outer membrane. Notably, VP07 exhibits structural similarity to a novobiocin derivative previously shown to bind LC3 with high confidence [41]. Based on these findings, we incorporated a VP07‐derived fragment as a putative autophagosome‐engaging module into the design of our hybrid molecules (Figure 1).

As the common linker for this proof‐of‐concept series, we selected N‐Boc‐4,7,10‐trioxa‐1,13‐tridecanediamine (3‐2‐2‐3‐Boc). Its three ether oxygen atoms provide greater polarity than a purely hydrocarbon linker, while its extended and conformationally flexible chain was expected to provide spatial separation between the mitochondrial‐targeting and LC3‐binding modules [42]. This choice was additionally supported by a precedent in autophagy‐targeting degrader design: a closely related 4,7,10‐trioxa‐1,13‐tridecanediamine‐derived spacer motif is present in AUTAC4, a mitochondria‐targeting AUTAC shown to promote the selective autophagic clearance of damaged mitochondria [43]. Furthermore, preliminary comparison of hybrid constructs containing alternative linkers indicated that compounds incorporating this linker exhibited the most favorable solubility under the conditions used for compound preparation and cellular assays (unpublished data). The terminal amino groups also enabled modular assembly through sequential amide‐bond formation. This modular synthetic strategy afforded a focused library of hybrid molecules, MG‐1–MG‐10, each comprising a mitochondrial‐interacting component and an autophagosome‐recruiting component. Systematic variation of substituents within both regions was implemented to enable preliminary exploration of structure–activity relationships.

Commercially available p‐hydroxybenzaldehyde derivatives bearing methoxy or bromo substituents in the 3‐ and 5‐positions were used as starting materials for the synthesis of the benzylidene–thiazolidinedione fragments (Figure S1). Introduction of the carboxylic acid functionality was achieved by alkylation of the corresponding phenols with tert‐butyl bromoacetate in dry DMF using potassium carbonate as a base, affording protected intermediates in nearly quantitative yields. Subsequent condensation of the substituted benzaldehydes with thiazolidinedione under Dean–Stark conditions in toluene, using piperidine as a catalyst, provided the corresponding benzylidene–thiazolidinedione adducts (Figure S1). The crude products were isolated by simple crystallization. Final deprotection of the tert‐butyl esters with trifluoroacetic acid in dichloromethane furnished the target carboxylic acids 1a–c (mitoNEET binding fragment, Figure S1) in overall yields exceeding 85% over three steps, without the need for chromatographic purification.

4‐Phenylquinazoline‐2‐carboxylic acid (**2**, TSPO binding fragment, Figure S1) was synthesized according to a previously reported procedure, starting from commercially available 2‐aminobenzophenone [44]. Condensation of the amine with glyoxylic acid in ethanol in the presence of ammonium acetate afforded the corresponding dihydroquinazolinone intermediate, which was subsequently subjected to photooxidative aromatization in DMF under ambient air using a tungsten lamp as the light source. After recrystallization, compound **2** was obtained as a beige solid in 57% yield.

Substituted isatoic anhydrides **3a,b** bearing substituents in the 4‐position were prepared via Baeyer–Villiger oxidation of commercially available substituted isatins using hydrogen peroxide in formic acid (Figure 1). Subsequent N‐alkylation with ethyl or benzyl bromide in the presence of sodium hydride afforded the corresponding N‐alkyl isatoic anhydrides **4a–c**. Condensation of these intermediates with sodium dimethyl malonate in DMF at elevated temperature yielded the corresponding methyl esters **5a–c** (Figure 1). The overall yield of this three‐step sequence was approximately 20%, and no chromatographic purification was required.

Direct amide coupling of the quinazoline fragment bearing a carboxylic acid group with the amine linker using standard peptide coupling reagents (e.g., HATU or EDC) was unsuccessful, presumably due to interference from the acidic enolic proton within the quinazoline core, which compromised chemoselectivity. Consequently, linker attachment was achieved via nucleophilic substitution of the methyl ester by the free amine of the 3‐2‐2‐3‐Boc linker (Figure 1). Methyl esters **5a–c** were refluxed in toluene with the linker to afford the corresponding amide intermediates. Following solvent removal, Boc deprotection was performed in situ using hydrogen chloride in dioxane, and the resulting amine hydrochlorides **6a–c** were purified by column chromatography.

Final conjugation of amines 6a–c with carboxylic acids **1a–c** and **2** was accomplished via HATU‐mediated amide bond formation in DMF using DIPEA as a base, affording the target hybrid compounds **MG 1–10** (Figure 1), which were isolated by column chromatography in good overall yields. Synthesized compounds were characterized NMR spectroscopy (Figures S11–S36).

### 2.2 Evaluation of Synthesized Chimeric Compounds as Autophagy Activators

To evaluate the ability of the synthesized hybrid molecules to activate autophagy, we performed an initial immunoblotting‐based screen in the human neuroblastoma cell line SH‐SY5Y using the canonical autophagy marker LC3. Each compound was tested at two concentrations (50 and 25 µm) under standard culture conditions and under conditions of mitochondrial damage induced by co‐incubation with the mitochondrial uncoupler FCCP (1 µm), which has been reported to induce autophagy at 5 µm [6] (Figure 2А). In this experimental design, FCCP was not used as a standalone autophagy inducer but rather as an uncoupler to reduce mitochondrial membrane potential, modeling a partially damaged mitochondrial state.

**FIGURE 2:**
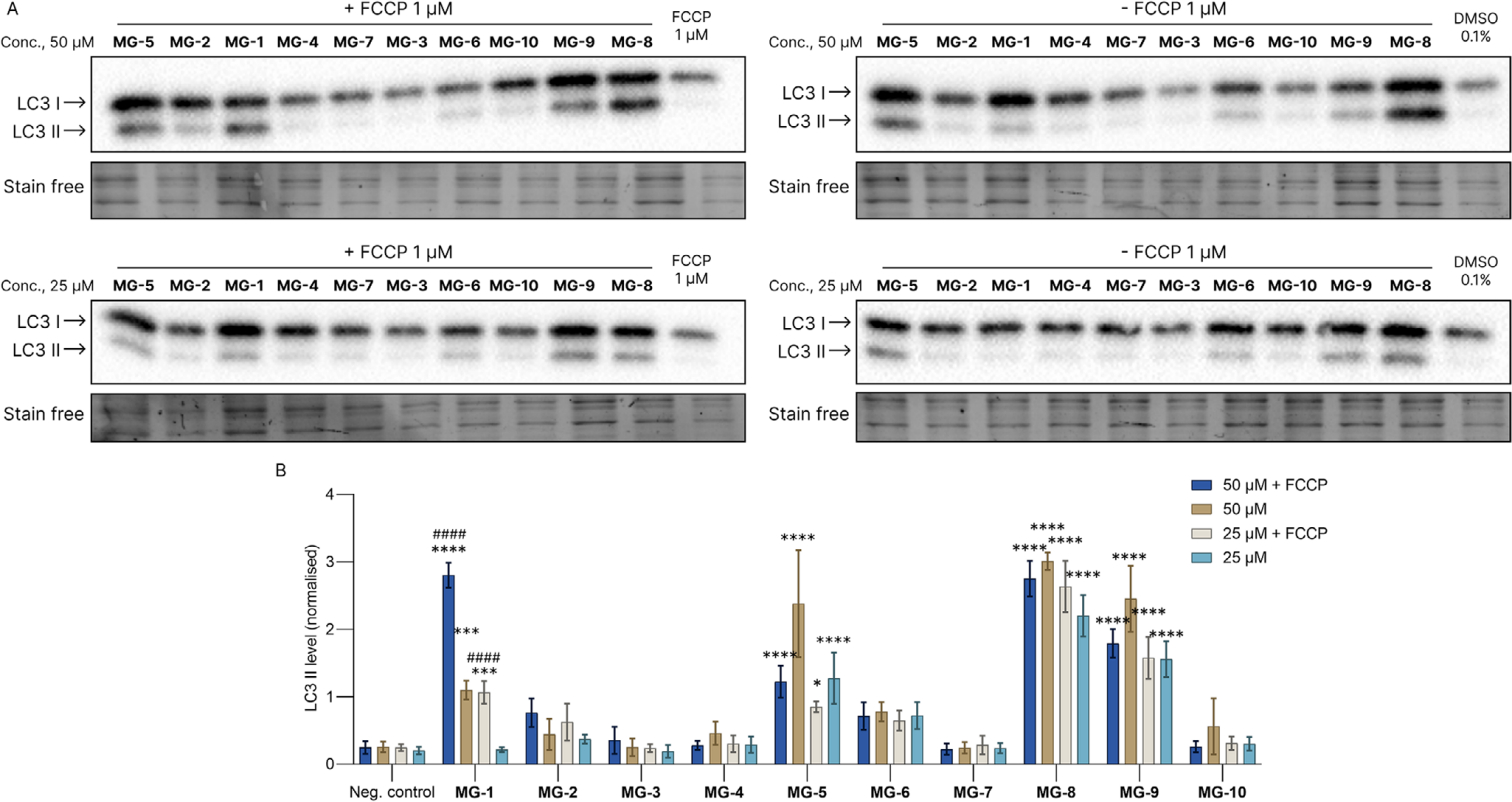
Screening of autophagy‐activating properties of **MG‐1 – MG‐10** series. (A) Western blotting of the key marker of autophagy (LC3) in cell lysates treated with **MG‐1** – **MG‐10** alone or in the presence of FCCP (1 µm). (B) Bar chart illustrating normalized levels of LC3 II, quantified on Western blots. Data represent mean ± SD (*n* = 3). The results of T‐tests are indicated on the graph. Comparisons between each sample and the corresponding negative control are indicated by asterisks: ^***^ *p* < 0.001, ^****^ *p* < 0.0001. Comparisons between samples treated with FCCP and the corresponding samples without FCCP are indicated by hash symbols: #### *p* < 0.0001.

Across the compound library, four hybrid molecules significantly induced autophagy under basal conditions and in the presence of mitochondrial damage (Figure 2). Within the TSPO‐directed subgroup, MG‐8 efficiently activated mitophagy under both conditions, MG‐9 showed a milder response, and MG‐10 was comparatively weak. However, the TSPO‐directed series also reduced SH‐SY5Y cell viability at concentrations overlapping with those used in the activity screen (Figure S2A–D). This profile differs from that of the previously reported TSPO‐directed mitochondrial ATTEC mT1, for which no pronounced cytotoxicity was observed under the tested conditions [27]. Owing to this unfavorable balance between efficacy and cellular tolerability, TSPO‐directed hybrids were excluded from further biological characterization.

For **MG‐1**, containing the mitoNEET module, autophagy induction was significantly potentiated upon FCCP treatment, indicating pronounced sensitivity to mitochondrial damage (Figures 2 and 3A). To determine whether this effect extends to other forms of mitochondrial stress, we induced oxidative stress by treating cells with antimycin A (1 µm) for 4 h prior to incubation with **MG‐1**. Under these conditions, **MG‐1** also displayed enhanced autophagy‐inducing activity (Figure S3). Collectively, these findings indicate that **MG‐1** does not function as a constitutive autophagy activator but instead exhibits stress‐dependent activity that is selectively enhanced under conditions of mitochondrial dysfunction.

**FIGURE 3:**
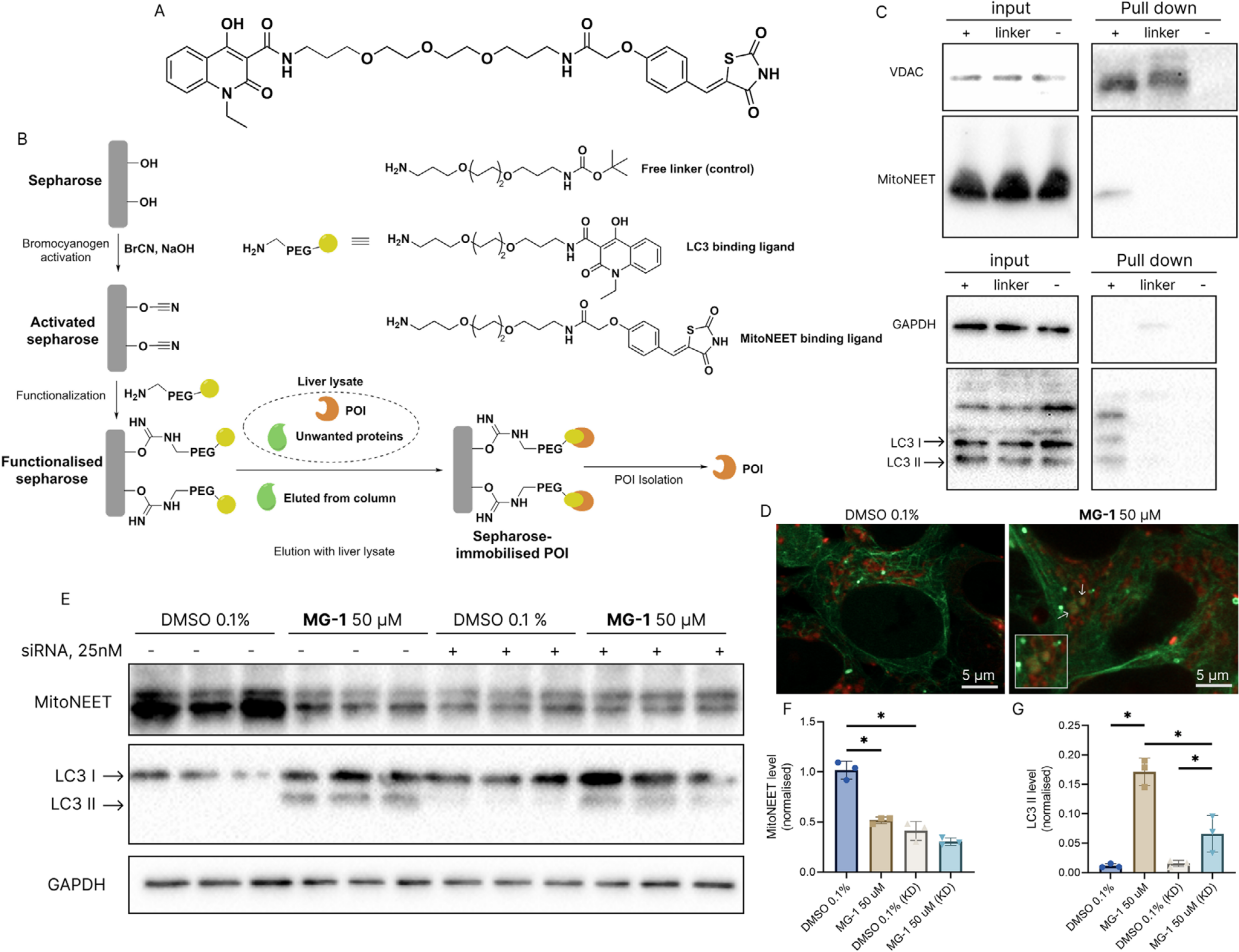
The lead compound **MG‐1** interacts with MitoNEET and LC3 in cells. (А) Structure of lead compound **MG‐1**. (B) Scheme of target‐protein verification pull‐down experiment. (C) Immunoblotting representing input and results of pull‐down. (D) Fluorescence microphotographs of SH‐SY5Y cells expressing LC3‐GFP and stained with LumiTracker Mito Red FM, treated with **MG‐1** and control cells. E. Immunoblotting representing levels of LC3 II in normal cells and with knockdown (KD) of *MitoNEET*. (F, G) Bar graph of quantified levels of MitoNEET (F) and LC3 II (G) normalized to GAPDH.

The dependence of **MG‐1** activity on different forms of mitochondrial stress, together with its relatively low cytotoxicity (Figure S2E,F), prompted us to further investigate the mechanism underlying its activity. We first examined whether the selected molecular architecture and its individual components were required for the observed activity. Control compounds corresponding to the individual structural modules of **MG‐1**, including the isolated mitochondria‐interacting ligands and the autophagosome‐recruiting fragment, failed to induce detectable autophagic flux (Figure S4). This lack of activity underscores the necessity of a hybrid molecular architecture and supports the conclusion that cooperative engagement of both functional modules is required to elicit a robust autophagic response.

We next obtained biochemical evidence that the functional moieties of MG‐1 associate with their proposed target proteins. Sepharose beads bearing the respective **MG‐1** moieties were synthesized and used for pull‐down assays from mouse liver lysates (Figure 3B,C). Immunoblotting showed that the corresponding target proteins were detected in eluates from beads bearing the respective MG‐1 moieties, but not in eluates from control beads containing the linker alone or from unconjugated beads. In contrast, control proteins, including GAPDH and VDAC, were not specifically enriched on beads bearing the tested moieties. At the cellular level, we also observed increased spatial proximity between mitochondria and LC3 following **MG‐1** treatment (Figure 3D and Figure S5).

We also observed a functional dependence of **MG‐1** activity on the presence of MitoNEET in cells (Figure 3E,G). MitoNEET‐knockdown cells (Figure 3E,F and Figure S10) failed to activate autophagy in response to MG‐1 treatment, as indicated by the low levels of LC3‐II (Figure 3G).

Taken together, these findings identify **MG‐1** as the lead hybrid molecule and demonstrate that its autophagy‐inducing activity is strongly enhanced under conditions of mitochondrial stress. The interaction of its functional moieties with the proposed targets, together with the loss of **MG‐1**‐induced autophagy following MitoNEET knockdown, supports the involvement of both MitoNEET and LC3 in its mechanism of action.

### 2.3 MG‐1 Induces Autophagy and Enhances Autophagic Flux

Treatment of SH‐SY5Y cells with **MG‐1** resulted in a marked increase in lysosomal abundance (Figure 4A and Figure S6) and a significant enhancement of autophagic flux, as assessed using the tandem GFP‐RFP‐LC3 reporter system (Figure 4C) [45, 46, 47] and by analysis of LC3‐II accumulation in the presence of the lysosomal inhibitor bafilomycin A1 (Figure S4). Combined treatment with **MG‐1** and bafilomycin A1 resulted in greater LC3‐II accumulation than either treatment alone, further supporting increased autophagic flux.

**FIGURE 4:**
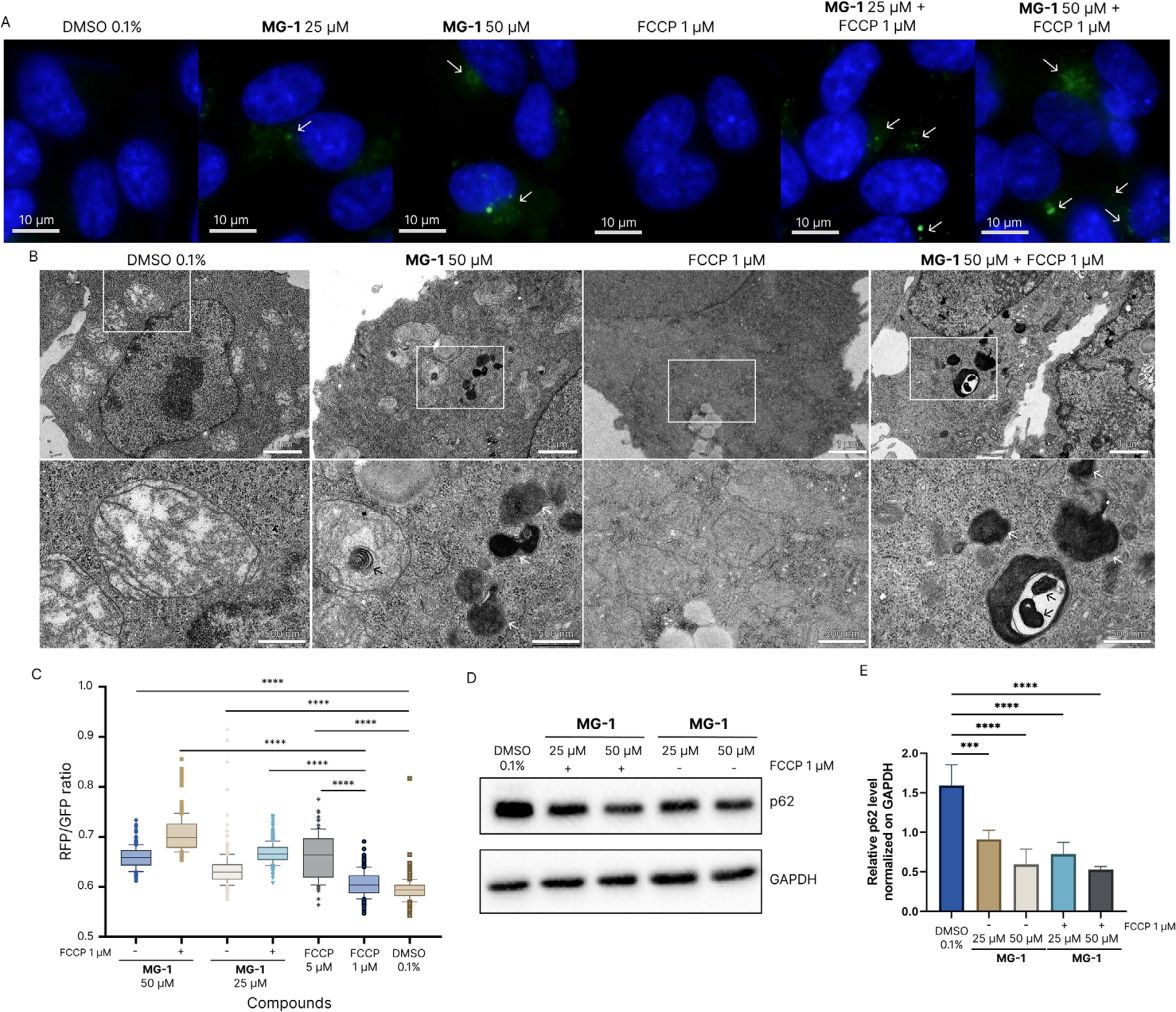
The lead compound MG‐1 induces autophagy. (А) Accumulation of lysosomes (green) in wild‐type human neuroblastoma cell line SH‐SY5Y upon 24‐h treatment with **MG‐1** alone or with **MG‐1** in combination with FCCP 1 µm or with control compounds. White arrows mark lysosomes. Nuclei (blue) are stained with Hoechst. (B) Transmission electron microphotographs showing accumulation of autophagosomes after **MG‐1** treatment alone or in combination with FCCP 1 µm. White arrows indicate autophagosomes with electron‐dense material; black arrows indicate autophagosomes with folded‐membrane structures. (C) Changes in the RFP/GFP ratio in the SH‐SY5Y pMXs GFP‐LC3‐RFP cell line when treated with **MG‐1** alone or with **MG‐1** in combination with FCCP 1 µm or with control compounds. “Plus” and “minus” correspond to the addition of FCCP 1 µm. The results of ANOVA tests are indicated on the graph: ^****^ – *p*‐value < 0.0001. (D) Western blotting of p62. (E) Bar graph indicating differences in p62 levels in SH‐SY5Y cells after **MG‐1** treatment. Data represent the mean ± SD (*n* = 3). The results of T‐tests are indicated on the graph: ^***^ – *p*‐value < 0.001, ^****^ – *p*‐value < 0.0001.

To further characterize the autophagic response at the ultrastructural level, we performed transmission electron microscopy. Ultrastructural analysis by transmission electron microscopy further revealed the accumulation of autolysosomal structures bounded by double membranes and ranging from approximately 500 nm to 1 µm in diameter (Figure 4B). These vesicles predominantly contained electron‐dense material, consistent with lipid‐ and protein‐rich cargo, as well as folded membranous structures indicative of ongoing organelle degradation.

The global increase in cellular degradative activity was further supported by a significant reduction in levels of the autophagy adaptor protein p62 following **MG‐1** treatment (Figure 4D,E). Importantly, co‐treatment with FCCP markedly potentiated all observed effects, including lysosomal accumulation, enhanced autophagic flux, and p62 degradation. These findings reinforce the conclusion that **MG‐1** activates autophagy preferentially under conditions of mitochondrial dysfunction, rather than through nonspecific induction.

Taken together, these findings demonstrate that **MG‐1** induces a coordinated autophagic response, characterized by increased lysosomal abundance, enhanced autophagic flux, accumulation of autolysosomal structures, and p62 degradation. The marked potentiation of these effects by FCCP further indicates that **MG‐1** promotes autophagic activity, particularly under conditions of mitochondrial stress.

### 2.4 Activation of Canonical Autophagy Regulatory Pathways by MG‐1

Autophagy initiation and progression are tightly regulated by two major signaling axes centered on the AMPK‐ULK1 and AKT‐mTOR pathways. To determine whether **MG‐1** engages canonical autophagy‐regulatory mechanisms, we analyzed the phosphorylation status of key signaling components within these pathways.

Under basal conditions, treatment of SH‐SY5Y cells with **MG‐1** at 50 µm resulted in a pronounced increase in AMPK phosphorylation, accompanied by enhanced phosphorylation of its downstream substrate ULK1 at the activating sites S555 and S317 (Figure 5A,B). Notably, under conditions of mitochondrial stress induced by partial membrane depolarization, comparable activation of the AMPK–ULK1 axis was observed at a lower concentration of **MG‐1** (25 µm), indicating that mitochondrial damage sensitizes cells to **MG‐1**‐mediated autophagy signaling (Figure 5A,B).

**FIGURE 5:**
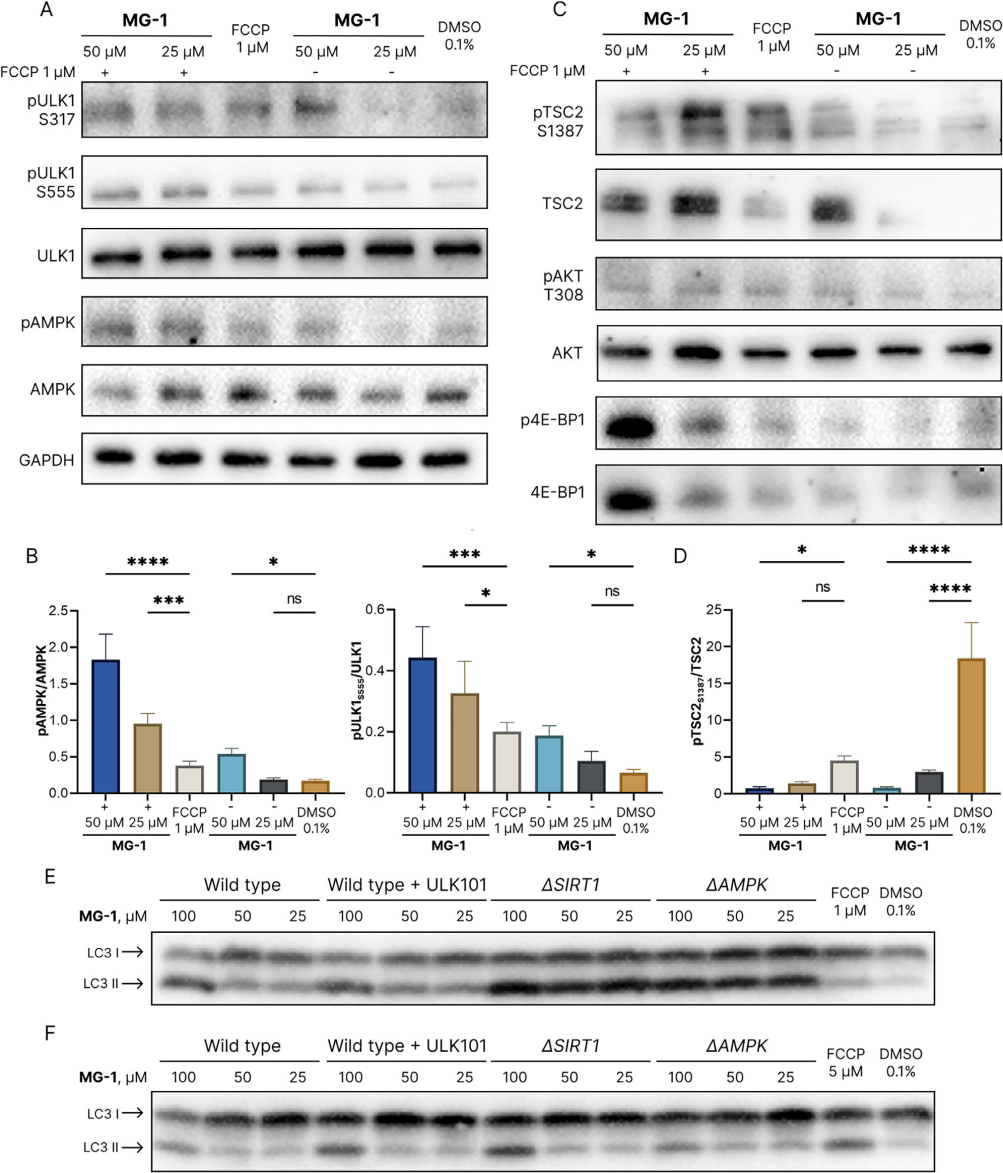
**MG‐1** modulates AMPK/ULK1‐associated autophagy signaling. (A) Western blotting of the AMPK/ULK1‐related components of the autophagy regulatory cascade. The specificity of the antibodies used is shown on the left side of the panel. Compounds added to SH‐SY5Y cell culture are indicated above the lanes. The GAPDH panel shown in Figure 5A serves as a representative loading control for the same set of experimental samples analyzed in Figure 5C. (B) Bar graphs indicating changes in phosphorylation levels of AMPK (left) and ULK (right) in SH‐SY5Y cells after MG‐1 treatment, normalized to GAPDH as a loading control. Data represent the mean ± SD (*n* = 3). The results of *t*‐tests are indicated on the graph: ^*^—*p*‐value < 0.05, ^***^ – *p*‐value < 0.001, ^****^ – *p*‐value < 0.0001. (C) Western blotting of the AKT/mTOR‐related components of the autophagy regulatory cascade. The specificity of the antibodies used is shown on the left side of the panel. Compounds added to SH‐SY5Y cell culture are indicated above the lanes. (D) Bar graphs indicating changes in phosphorylation level of TSC2 in SH‐SY5Y cells after **MG‐1** treatment. Data represent the mean ± SD (*n* = 3). The results of *t*‐tests are indicated on the graph: ^*^—*p*‐value < 0.05, ^****^ – *p*‐value < 0.0001. (E, F) Immunoblotting of the key marker of autophagy (LC3) after cell treatment with **MG‐1** in combination with FCCP 1 µm (E) or alone (F). Cells with inactivated *AMPK* or *SIRT1* genes, or cells that had undergone additional treatment with the ULK1 inhibitor ULK101, were used.

Analysis of signaling pathways involved in autophagy regulation revealed that **MG‐1** treatment led to a significant reduction in TSC2 phosphorylation at S1387 (Figure 5C,D). In contrast, phosphorylation levels of AKT itself, as well as mTOR activity as assessed by phosphorylation of 4E‐BP1, remained largely unchanged (Figure 5C). This pattern suggests that **MG‐1** does not induce a global suppression of the AKT‐mTOR axis but instead modulates specific regulatory nodes associated with autophagy control.

Notably, although **MG‐1** induced phosphorylation of AMPK and ULK1, its ability to activate autophagy was resistant to genetic inactivation of AMPK and its upstream activator SIRT1, as well as to pharmacological inhibition of ULK1 using the selective antagonist ULK101 (Figure 5E,F).

Taken together, these data indicate that **MG‐1** engages canonical autophagy‐associated signaling pathways without functioning as a direct activator or inhibitor of individual kinases. This mode of action is consistent with the behavior expected for a chimeric molecule that promotes autophagy downstream of mitochondrial damage rather than enforcing constitutive pathway activation.

### 2.5 MG‐1 Promotes Autophagic Clearance of Damaged Mitochondria Under Conditions of Mitochondrial Stress

Given the chimeric design of **MG‐1** and the pronounced enhancement of its activity under conditions of mitochondrial membrane depolarization, we next examined whether **MG‐1** facilitates mitophagy.

Using fluorescent co‐staining of mitochondria and lysosomes, together with tandem fluorescent mitochondrial reporters, we observed that co‐treatment with **MG‐1** and a subtoxic concentration of FCCP (1 µm) resulted in pronounced mitochondrial fragmentation followed by mitochondrial clearance in three independent cell lines‐SH‐SY5Y neuroblastoma cells, HEK293T cells, and SV40‐transformed MRC‐5 fibroblasts (Figure 6A,B and Figure S7A,B).

**FIGURE 6:**
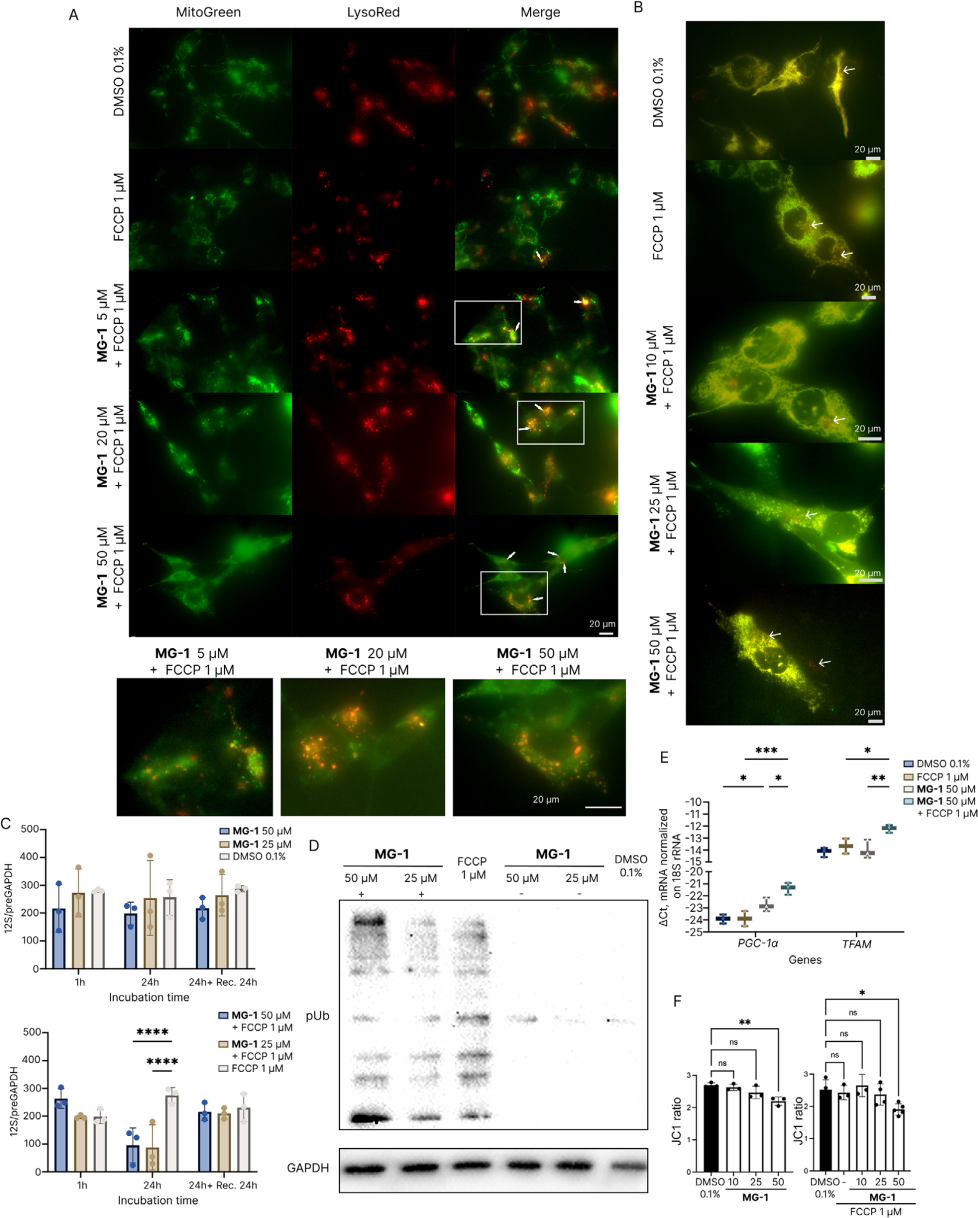
**MG‐1** is an efficient mitophagy activator. (A) Human neuroblastoma cells (SH‐SY5Y) were treated with FCCP (1 µm) and various concentrations of **MG‐1**, incubated for 24 h, and then stained with MitoTracker Green (200 nm, 15 min) and LysoTracker Red (150 nm, 15 min). The lower panel shows mitochondria in lysosomes at higher magnification. White arrows indicate mitochondria in lysosomes. (B) MRC‐5 SV40 cells transduced with the mito‐RFP‐GFP construct were treated with FCCP (1 µm) and various concentrations of **MG‐1**, followed by incubation for 24 h. White arrows indicate mitochondria in lysosomes. (C) Number of mitochondria evaluated by quantitative PCR of mtDNA (*12S* gene) vs. nuclear DNA (*GAPDH* gene) from the wild‐type SH‐SY5Y cells after treatment with **MG‐1** alone (up) and in combination with FCCP 1 µm (down) for 1, 24 h, or 24 h followed by recovery (rec.). Data represent the mean ± SD (*n* = 3). The results of the t‐test are indicated on the graph: ^***^ *p*‐value < 0.001. (D) Western blotting of the key marker of mitophagy, phospho‐ubiquitin, after treatment of cells with **MG‐1** alone or with **MG‐1** in combination with FCCP 1 µm. (E) mRNA levels of TFAM and PGC‐1α normalized to 18S RNA (ΔCt). Data represent the mean ± SD (*n* = 3). The results of the t‐test are indicated on the graph: ^*^ *p*‐value < 0.05, ^**^ *p*‐value < 0.01, ^***^ *p*‐value < 0.001. (F) Effect of various concentrations of **MG‐1** on the membrane potential of mitochondria in SH‐SY5Y cells in the presence of 1 µm FCCP (right panel) or without FCCP (left panel). Cells were incubated with the specified compounds for 1 h, then washed and stained with the ratiometric dye JC1 (1 µg/mL for 30 min). Data represent the mean ± SD (*n* = 3).

In the neuroblastoma human cell line, FCCP alone induced moderate mitochondrial fragmentation after 24 h of incubation (Figure 6A). Under these conditions, **MG‐1** markedly potentiated mitochondrial fragmentation, with detectable effects observed at concentrations as low as 5 µm. At higher concentrations (20–50 µm), a substantial reduction in mitochondrial content was evident, accompanied by increased colocalization of mitochondrial markers with lysosomal compartments, consistent with enhanced autophagic degradation.

Comparable effects were observed in SV40‐transformed MRC‐5 fibroblasts (Figure 6B) and HEK293T cells (Figure S7A,B) expressing tandem fluorescent mitochondrial reporters (mCherry‐GFP‐Fis1 or TOM20MTS‐mCherry‐EGFP). In these systems, **MG‐1** treatment in the presence of FCCP promoted mitochondrial fragmentation followed by efficient clearance of mitochondrial material. The accumulation of red‐only puncta further indicated delivery of mitochondrial fragments to acidic degradative compartments.

Ultrastructural analysis by transmission electron microscopy supported these findings, revealing an increased number of autolysosomes containing folded membranous structures and fragmented mitochondria in cells co‐treated with **MG‐1** and FCCP (Figure 4B and Figure S7C). Consistent with enhanced mitochondrial turnover, quantitative PCR analysis and immunoblotting demonstrated a significant reduction in mitochondrial DNA and protein levels following 24 h of co‐incubation with **MG‐1** and FCCP, whereas **MG‐1** or FCCP alone produced only minor effects (Figure 6C and Figure S8).

Further support for **MG‐1**–induced mitophagy was provided by an increase in phospho‐ubiquitination levels following treatment (Figure 6D). In line with the previous observations, the mitophagy‐stimulating properties of **MG‐1** were markedly enhanced under conditions of mitochondrial stress.

Importantly, MG‐1 treatment not only promoted the efficient clearance of damaged mitochondria but also stimulated mitochondrial biogenesis, as evidenced by increased *TFAM* and *PGC‐1α* expression (Figure 6E). This was accompanied by a complete recovery of mitochondrial DNA and protein levels after the removal of FCCP and **MG‐1** (Figure 6C and Figure S8).

Importantly, **MG‐1** did not induce substantial mitochondrial depolarization on its own at concentrations relevant for autophagy induction. JC‐1 staining revealed that a measurable decrease in mitochondrial membrane potential occurred only at the highest concentration tested (50 µm, Figure 6F). In contrast, mitochondrial clearance and autophagy‐associated mitochondrial degradation were evident at substantially lower concentrations (≥ 5 µm) when mitochondrial stress was externally imposed by FCCP. To assess mitotoxicity, we cultured MG‐1‐treated cells in galactose‐containing medium, which increases cellular reliance on mitochondrial oxidative metabolism (Figure S2E,F). Only the highest concentrations of **MG‐1** (CC_50_ 75 µm), exceeding those needed to activate selective mitophagy of damaged mitochondria, appeared to decrease cell viability in the galactose medium.

Taken together, these results demonstrate that **MG‐1** enhances the autophagic removal of dysfunctional mitochondria under conditions of mitochondrial impairment and promotes mitochondrial biogenesis. Rather than directly inducing mitochondrial damage or engaging a single dedicated mitophagy pathway, **MG‐1** appears to lower the threshold for autophagic degradation of damaged organelles. This behavior is consistent with a chimeric mode of action in which mitochondrial stress serves as a permissive enabling factor for efficient coupling of damaged mitochondria to the autophagic machinery.

### 2.6 MG‐1 Exhibits Low Toxicity to Cells and Animals Within the Active Concentration Range

The toxicity of **MG‐1** was first evaluated in four distinct cell lines (Va13, SH‐SY5Y, HEK293 and Jurkat) across six concentrations: 0.1, 0.4, 2, 10, 50, and 100 µm (Figure 7A). The CC_50_ was determined to be 66.65 µm ± 8.1 for Jurkat and >100 µm for SH‐SY5Y, HEK293, and Va13. In the most sensitive cellular assays (Figure 7A), **MG‐1** exhibited measurable biological activity at concentrations as low as 5 µm, corresponding to a 12‐ to > 20‐fold separation between the active concentration range and cytotoxicity thresholds. This concentration window indicates that **MG‐1** exerts its cellular effects at doses that are well tolerated in vitro.

**FIGURE 7:**
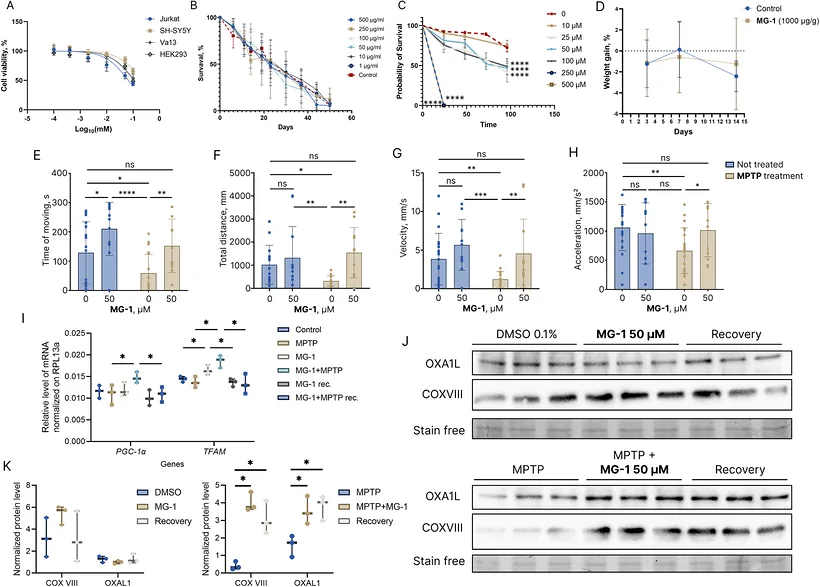
Investigation of toxicity rates of **MG‐1**. (A) Viability of human neuroblastoma (SH‐SY5Y), human lung fibroblasts (VA‐13), Human embryonic kidney (HEK293), and human T lymphocyte (Jurkat) cells incubated with **MG‐1** for 24 h. MTT assay; data are the mean ± SD. (B) *Caenorhabditis elegans* lifespan under the influence of **MG‐1**. The results are expressed as the mean ± standard error of the mean (SEM) from five independent replicates. (C) The dynamics of the survival of *Danio rerio* fry exposed to **MG‐1**. ^****^—*p* < 0.0001 by the Mantel‐Cox survival curve test. (D) Dynamics of body weight gain in ICR mice after a single administration of **MG‐1** at a dose of 1000 µg/g body weight or vehicle—corn oil. (E–H) Investigation of **MG‐1** efficiency in the MPTP model of Parkinson's disease in *Danio rerio*. Values of fry time of moving (E), velocity (F), total distance (G), and acceleration (H) under treatment with **MG‐1** and MPTP in combination with **MG‐1**. Data are presented as mean ± standard deviation. Two‐way ANOVA (factors: MPTP, **MG‐1**). ^*^—*p* < 0.05, ^**^—*p* < 0.01, ^***^—*p* < 0.001, ^****^—*p* < 0.0001. (I) mRNA levels of TFAM and PGC‐1α normalized to 18S RNA (ΔCt). Data represent mean ± SD (*n* = 3). The results of the Mann‐Whitney test are indicated on the graph: ^*^ – *p*‐value = 0.05. (J) Immunoblotting with antibodies against OXAL1 and COX VIII from Danio rerio embryos treated with MG‐1 under normal conditions or in the MPTP model of Parkinson's disease. (K) Bar graphs representing relative values of OXAL1 and COX VIII normalized to total protein loading (stain free). Data represents mean ± SD (*n* = 3). The results of Mann‐Whitney test are indicated on the graph: ^*^ – *p*‐value = 0.05.

To assess organism‐level toxicity, **MG‐1** was next evaluated in *Caenorhabditis elegans*, a widely used model for systemic toxicity and stress responses. Across the entire tested concentration range (1–500 µg/mL), **MG‐1** did not adversely affect nematode survival or lifespan (Figure 7B), indicating no overt toxicity in this invertebrate model.

In contrast, assessment in *Danio rerio* embryos and early larval stages revealed dose‐dependent toxicity. At concentrations of 200 and 500 µm, pronounced developmental toxicity and lethality were observed, while intermediate concentrations resulted in partial viability loss, yielding an estimated LD_50_ in the range of 50–100 µm (Figure 7C). It should be noted that early zebrafish developmental stages represent a particularly sensitive vertebrate system, and toxic effects in this model often occur at concentrations substantially lower than those tolerated by adult organisms.

Finally, tolerability of **MG‐1** was assessed in mice following single oral administration at a dose of 1000 µg/g body weight. Due to the very low solubility of MG‐1 in water, corn oil was used as a vehicle. No signs of acute distress, behavioral abnormalities, or deviations in body weight gain were observed during the monitoring period when compared with control animals (Figure 7D). Post‐mortem examination revealed no detectable changes in gross organ morphology or organ weight in **MG‐1**‐treated mice relative to controls (Figure S9).

Collectively, these data indicate that **MG‐1** exhibits a moderate toxicity profile. Although high concentrations are detrimental in sensitive developmental models, **MG‐1** is well tolerated at concentrations relevant to its biological activity in cellular systems and does not induce overt acute toxicity in adult organisms following single‐dose administration. This safety profile supports the suitability of **MG‐1** for further pharmacological investigation.

### 2.7 MG‐1 Improves Locomotor Performance in a *Danio rerio* Model of MPTP‐Induced Mitochondrial Dysfunction

To evaluate the functional consequences of **MG‐1** activity in vivo under conditions of mitochondrial impairment, we employed an MPTP‐induced model of Parkinson's disease‐like syndrome in *Danio rerio*, a well‐established system for studying mitochondrial toxicity and dopaminergic dysfunction [48]. Importantly, *D. rerio* possesses a MitoNEET ortholog (Figure S10), providing a relevant molecular target for **MG‐1** in this system. We therefore investigated whether **MG‐1** could ameliorate behavioral deficits associated with mitochondrial damage and promote mitochondrial biogenesis.

Exposure of *Danio rerio* fry to MPTP at a concentration of 5 µg/mL resulted in a pronounced reduction of locomotor activity, as assessed by the open field test. Specifically, MPTP‐treated animals exhibited significant decreases in total distance traveled, average velocity, acceleration, and overall movement time compared with control fry (Figure 7E–H), consistent with impaired motor function.

Notably, co‐treatment with **MG‐1** at 50 µm significantly attenuated MPTP‐induced locomotor deficits. Under these conditions, all measured behavioral parameters were restored to levels comparable to those observed in the control group (Figure 7E–H).

Importantly, administration of **MG‐1** alone did not produce significant changes in total distance traveled, swimming velocity, or acceleration relative to untreated controls. A modest increase in total movement time (approximately 1.5‐fold) was observed; this change was not accompanied by alterations in speed or acceleration (Figure 7E–H), indicating that **MG‐1** does not act as a general stimulant of locomotor activity.

To further investigate the molecular effects of **MG‐1** under basal and mitochondrial stress conditions, we analyzed the expression of key regulators of mitochondrial biogenesis. **MG‐1** significantly increased *PGC‐1α* expression only under conditions of MPTP‐induced mitochondrial stress (Figure 7I). Specifically, *PGC‐1α* expression was significantly higher in the **MG‐1** + MPTP group than in the MPTP group and returned to basal levels after compound withdrawal. In contrast, *TFAM* expression was significantly increased both in the **MG‐1**‐treated group and in the **MG‐1** + MPTP group, with a tendency toward stronger induction in MPTP‐treated fish. *TFAM* expression also returned to baseline following **MG‐1** withdrawal.

To determine whether these transcriptional changes were followed by restoration of mitochondrial protein abundance, we analyzed the mitochondrial inner membrane proteins OXA1L and COX VIII (Figure 7J,K). No significant differences were observed between the Control, **MG‐1**, and **MG‐1** recovery groups. In contrast, co‐treatment with **MG‐1** significantly restored OXA1L and COX VIII levels compared with the MPTP group. Notably, this restoration persisted in the corresponding recovery group despite normalization of *PGC‐1α* and *TFAM* expression. The transient induction of mitochondrial biogenesis markers, followed by the persistent restoration of mitochondrial protein levels, is consistent with an adaptive response aimed at restoring mitochondrial homeostasis following mitochondrial stress.

Taken together, these findings indicate that **MG‐1** does not enhance locomotion under physiological conditions but can restore motor function in *Danio rerio* subjected to mitochondrial toxic stress. This context‐dependent behavioral effect is consistent with a model in which **MG‐1** modulates cellular responses to mitochondrial dysfunction rather than exerting nonspecific neurostimulatory activity. The concomitant activation of mitochondrial biogenesis and restoration of mitochondrial protein levels provide a potential molecular basis for the functional improvement observed following **MG‐1** treatment under conditions of mitochondrial stress.

## 3 Discussion

In parallel with growing interest in pharmacological modulation of autophagy, bifunctional small‐molecule chimeras have emerged as a promising strategy for selective intracellular degradation. Most reported systems, including AUTACs and ATTECs, have been developed to target individual proteins by coupling a protein‐binding ligand to motifs that recruit the autophagic machinery, most commonly LC3 or ubiquitin‐dependent receptors. By contrast, applications of this concept to whole organelles remain limited. In particular, only a small number of chimeric small molecules capable of inducing mitophagy have been described, and these typically lack selectivity toward functionally impaired mitochondria, often promoting mitochondrial degradation in a largely state‐independent manner [23, 24]. Moreover, existing approaches frequently rely on global ubiquitination or constitutive recruitment of autophagosomes, raising concerns about disruption of mitochondrial homeostasis. These limitations underscore a significant gap in current strategies and highlight the need for mitophagy modulators that are both organelle‐directed and responsive to mitochondrial damage.

Dysregulation of autophagy has been causally linked to a broad spectrum of human pathologies, including neurodegenerative disorders, lysosomal storage diseases, and metabolic and autoimmune conditions [49, 50, 51, 52]. In the nervous system, where post‐mitotic neurons are particularly vulnerable to the accumulation of dysfunctional organelles, impaired autophagic clearance represents a central pathogenic mechanism underlying diseases such as Parkinson's disease, Alzheimer's disease, and amyotrophic lateral sclerosis.

Aging is accompanied by a progressive decline in autophagic capacity [53], leading to mitochondrial dysfunction and the accumulation of senescent cellular phenotypes [54]. This age‐associated erosion of quality‐control mechanisms is especially detrimental in neurons, where even modest impairments in mitochondrial turnover can precipitate energy failure, oxidative stress, and neurodegeneration. Accordingly, restoration of efficient mitochondrial quality control, and mitophagy in particular, has emerged as an attractive therapeutic strategy for neurodegenerative disease intervention.

Despite extensive efforts to pharmacologically stimulate autophagy and mitophagy, most currently known activators, both natural and synthetic, operate through global perturbation of cellular metabolism or mitochondrial function and frequently act as intrinsic stressors or toxins [17, 55]. Such nonspecific activation is fundamentally incompatible with long‐term therapeutic application and underscores the need for approaches that selectively enhance autophagic clearance without compromising cellular homeostasis.

In this study, we describe the design, synthesis, and biological evaluation of a series of hybrid small molecules designed to selectively enhance the autophagic clearance of damaged mitochondria. Rather than functioning as classical enzyme inhibitors or receptor agonists, these compounds were conceived as chimera‐like molecules that integrate mitochondrial‐interacting elements with fragments capable of engaging the autophagic machinery.

A central finding of this work is that activation of autophagy by the synthesized hybrids is strongly dependent on mitochondrial stress. Across multiple cellular readouts, **MG‐1** consistently displayed minimal activity under basal conditions but robustly enhanced autophagy in the presence of partial mitochondrial depolarization. This behavior clearly distinguishes **MG‐1** from conventional autophagy inducers that act through constitutive modulation of upstream signaling pathways.

Importantly, individual structural modules corresponding to the mitochondrial‐interacting ligands or the autophagosome‐recruiting fragment were inactive when tested in isolation. This observation argues against nonspecific activity of the individual components and supports the conclusion that the hybrid molecular architecture itself is required for biological function.

Among the mitochondrial‐targeting strategies evaluated, hybrids incorporating a mitoNEET‐directed moiety exhibited the most favorable balance between autophagy‐inducing activity and cellular tolerability. In contrast, TSPO‐based hybrids displayed limited efficacy and increased cytotoxicity, leading to their exclusion from further analysis. These findings indicate that not all outer mitochondrial membrane targets are equally compatible with a chimeric autophagy‐directing strategy and highlight mitoNEET as a particularly suitable node for damage‐responsive engagement.

MitoNEET is a redox‐sensitive protein associated with the regulation of mitochondrial iron and redox homeostasis. Our pull‐down experiments supported the association of the MitoNEET‐ and LC3‐directed moieties of **MG‐1** with their respective target proteins. Moreover, depletion of MitoNEET markedly reduced **MG‐1**‐induced LC3‐II accumulation, indicating that the autophagy‐inducing activity of **MG‐1** is functionally dependent on the presence of MitoNEET. These findings provide experimental support for the involvement of MitoNEET in **MG‐1** activity, while the molecular basis of the enhanced response under conditions of mitochondrial stress remains to be established. The pronounced potentiation of **MG‐1** activity following mitochondrial depolarization may therefore reflect increased accessibility of damaged mitochondria to the autophagic machinery or other stress‐dependent changes that facilitate their recognition and degradation. Further studies will be required to determine how mitochondrial stress influences MG‐1 interactions with MitoNEET and the subsequent recruitment of mitochondria into the autophagic pathway.

At the signaling level, **MG‐1** engaged canonical autophagy‐regulatory pathways without acting as a direct inhibitor or activator of individual kinases. These observations suggest that MG‐1 does not impose autophagy through a single dominant signaling node but instead lowers the activation threshold of the autophagic machinery, thereby facilitating the recognition and clearance of damaged mitochondria.

The ability of **MG‐1** to promote autophagic clearance of dysfunctional mitochondria was supported by multiple orthogonal assays across several cell types. Notably, **MG‐1** did not function as a primary mitochondrial uncoupler. Instead, it selectively amplified the cellular response to pre‐existing mitochondrial damage, consistent with a permissive or sensitizing role rather than a damaging one.

From a translational perspective, **MG‐1** exhibited an acceptable cellular and organism‐level tolerability within its active concentration range. Importantly, **MG‐1** improved locomotor performance in *a Danio reri*o model of MPTP‐induced mitochondrial dysfunction without affecting baseline locomotion, further supporting its context‐dependent activity and arguing against nonspecific neurostimulatory effects. At the molecular level, **MG‐1** treatment under mitochondrial stress was associated with activation of a mitochondrial biogenesis program, as evidenced by increased expression of *PGC‐1α* and *TFAM*, accompanied by restoration of the mitochondrial proteins OXA1L and COX VIII. Notably, mitochondrial protein levels remained restored after **MG‐1** withdrawal, whereas *PGC‐1α* and *TFAM* expression returned to baseline, suggesting a transient adaptive response that contributes to restoration of mitochondrial homeostasis.

Taken together, these findings support a model in which **MG‐1** acts as a stress‐responsive modulator of autophagy, enhancing the clearance of dysfunctional mitochondria under conditions of mitochondrial impairment while engaging adaptive mechanisms to support restoration of mitochondrial homeostasis. The combined evidence for MitoNEET and LC3 target engagement, functional dependence on MitoNEET, stress‐enhanced autophagic activity, and restoration of mitochondrial protein abundance provides a mechanistic framework for this context‐dependent activity. Although the precise molecular events linking mitochondrial stress to enhanced **MG‐1** activity remain to be elucidated, the present study establishes a proof of concept for small‐molecule chimeras that couple mitochondrial targeting to the autophagic machinery. The modular architecture and conditional activity profile of **MG‐1**, together with its beneficial effects in an in vivo model of mitochondrial dysfunction, provide a foundation for further optimization and mechanistic refinement, with potential relevance to diseases characterized by mitochondrial dysfunction.

## 4 Conclusions

In this study, we developed a chimera‐based strategy for selective modulation of autophagy and mitophagy through functional coupling of mitochondrial‐interacting and autophagosome‐engaging modules. Using this approach, we synthesized a focused library of hybrid molecules and identified **MG‐1** as a lead compound exhibiting damage‐dependent autophagy‐ and mitophagy‐enhancing activity.

**MG‐1** displayed minimal activity under basal conditions but robustly amplified autophagic flux and mitochondrial clearance in the presence of mitochondrial stress, distinguishing it from conventional autophagy inducers that act through constitutive pathway activation. Mechanistically, **MG‐1** engaged canonical autophagy‐regulatory pathways without functioning as a direct inhibitor or activator of individual kinases and did not function as a primary mitochondrial uncoupler. Pull‐down experiments confirmed the association of the MitoNEET‐ and LC3‐directed moieties with their respective target proteins, and MitoNEET depletion reduced MG‐1‐induced autophagy, providing experimental support for MitoNEET's involvement in its mechanism of action. Instead, MG‐1 lowered the activation threshold of the autophagic machinery, thereby selectively promoting the clearance of dysfunctional mitochondria.

Among the evaluated mitochondrial‐targeting strategies, incorporation of a mitoNEET‐directed moiety provided the most favorable balance between activity and tolerability. **MG‐1** exhibited acceptable cellular and organism‐level tolerability within its active concentration range and improved locomotor performance in a *Danio rerio* model of MPTP‐induced mitochondrial dysfunction without affecting baseline behavior. In this model, **MG‐1** treatment under mitochondrial stress was also associated with activation of a mitochondrial biogenesis program and restoration of mitochondrial protein abundance, consistent with an adaptive response supporting mitochondrial homeostasis.

Collectively, these findings establish a proof of concept for small‐molecule, damage‐gated chimeras that enhance mitochondrial quality control by selectively coupling damaged mitochondria to the autophagic machinery. By combining stress‐dependent mitochondrial clearance with adaptive restoration of the mitochondrial protein pool, **MG‐1** promotes a coordinated response to mitochondrial dysfunction rather than directly inducing mitochondrial damage. This strategy provides a promising foundation for developing next‐generation mitophagy modulators with potential relevance to neurodegenerative disease settings.

## Author Contributions

Conceptualization, E.A.G., A.Y.R., O.A.D., P.V.S.; methodology, R.M.O., E.A.G., A.Y.R., K.G.L., M.V.B., E.A.T.; validation, E.A.G., A.Y.R., K.G.L., E.I.M., V.I.P.; formal analysis, E.A.G., A.Y.R., J.A.G.; investigation, R.M.O., E.A.G., A.Y.R., K.G.L., S.Y.S., J.A.G., M.V.B., N.V.A., O.V.F., M.O.E., E.A.T., M.S.K., V.M.P., D.K.Y., E.Y.P., E.I.M., R.Z., V.B.V., I.M.V.; data curation, R.M.O., E.A.G., A.Y.R., M.V.B., E.A.T., E.Y.P.; writing – original draft preparation, R.M.O., E.A.G., A.Y.R.; writing – review and editing, K.G.L., J.A.G, E.A.T., E.I.M., V.I.P., O.A.D., P.V.S.; visualization, A.Y.R., E.A.G.; supervision, E.A.G., A.Y.R., E.I.M., M.L.L., L.E.B., A.N.M., V.I.P., P.V.S.; project administration, E.A.G., A.Y.R., O.A.D., P.V.S.; funding acquisition, E.A.G.; All authors have read and agreed to the published version of the manuscript.

## Funding

The study was funded by the Russian Science Foundation grant (agreement No. № 25‐74‐31004).

## Ethics Statement

The animal study protocol was approved by the Institutional Ethics Committee of A.N. Belozersky Institute of Physico‐Chemical Biology at the Moscow State University (protocol #3 on February 12, 2018), Ethics Committee of Institute of Mitoengineering, M.V. Lomonosov Moscow State University, (protocol #191 on March 1, 2024) and Committee for Control over Care and Use of Laboratory Animals of BIBCh RAS (IACUC) (protocol number 1004/24 from 04.11.2024).

## Conflicts of Interest

The authors declare no conflicts of interest.

## Supporting information




**Supporting File**: advs78570‐sup‐0001‐SuppMat.pdf.

## Data Availability

The data that supports the findings of this study are available in the supplementary material of this article.
