# Supplementary material for: A Damage‐Gated Small‐Molecule Chimera Selectively Enhances Mitophagy by Coupling Dysfunctional Mitochondria to Autophagy
Source: Adv Sci (Weinh). 2026 Oct 29:e78570. Online ahead of print. doi: 10.1002/advs.78570 (PMC13720385; doi:10.1002/advs.78570)
Supplement: Supplementary file 1 [file ADVS-9999-e78570-s001.pdf]

Supplementary Material

A Damage-Gated Small-Molecule Chimera Selectively Enhances Mitophagy by Coupling Dysfunctional Mitochondria to Autophagy

Ratislav M. Ozhiganov[1,2+], Ekaterina A. Guseva[1,2+], Alexander Yu. Rudenko[1,2+], Konstantin G. Lyamzaev[1], Sofya Y. Sokolskaya[3], Julia A. Golubeva[1,2], Maria V. Belopolskaya[4], Nadezda V. Andrianova [1], Olga V. Fadeeva[4], Elena O.Morgun[6], Elena A. Tukhovskaya[7], Marina S. Kazakova[7], Vladimir M. Pavlov[7], Daria K. Yagoda[8], Elena I. Marusich[9], Rose-Lys Zaranaina[10], Valeriya B. Vays[1], Irina M. Vangeli[1], Egor Y. Plotnikov[1], Maxim L. Lovat[4,5], Lora E. Bakeeva[1], Arkady N. Murashev[7], Vladimir I. Polshakov[2], Olga A. Dontsova[1,2,7], Peter V. Sergiev[1,2]

**equal contribution*

[1]Belozersky Institute of Physico-Chemical Biology, Lomonosov Moscow State University, Moscow, Russia

[2]Chemical Department, Lomonosov Moscow State University, Moscow, Russia

[3]Faculty of Fundamental Medicine, M.V. Lomonosov Moscow State University, Moscow, Russia

[4]Institute of Mitoengineering, Lomonosov Moscow State University, Moscow, Russia

[5]Biological Faculty, Lomonosov Moscow State University, Moscow, Russia

[6]Pirogov Russian National Research Medical University, Moscow, Russia

[7]Shemyakin-Ovchinnicov Institute of Bioorganic Chemistry (branch), Russian Academy of Sciences, Pushchino , Russia

[8]Faculty of bioengineering and bioinformatics, Lomonosov Moscow State University, Moscow, Russia

[9]Moscow Center for Advanced Studies, Moscow, Russia

[10]Peoples' friendship university of Russia named after Patrice Lumumba, Moscow, Russia

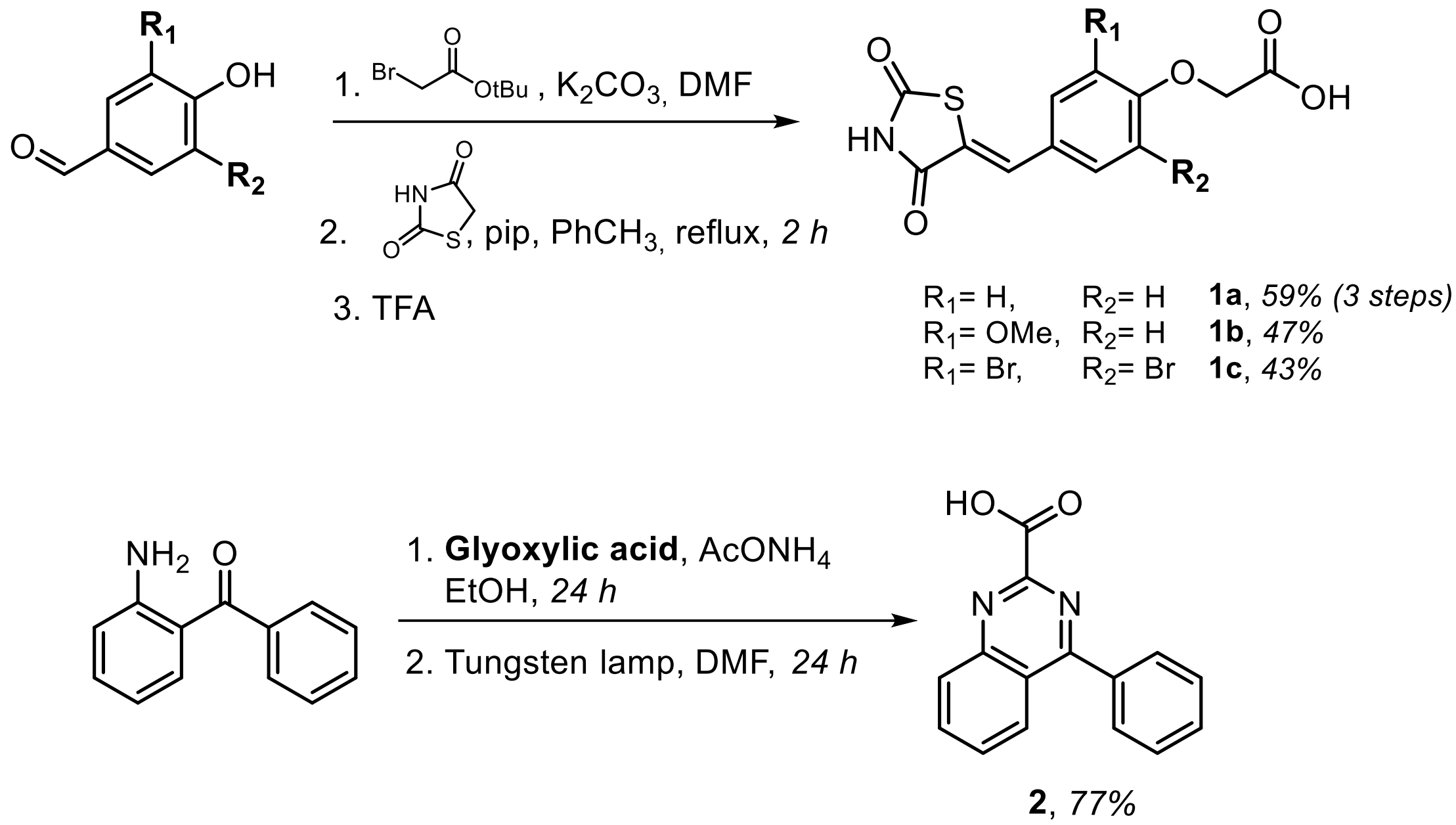

Supplementary Figure S1. Synthesis of benzylidene-thiazolidinedione derivatives 1a–c. Synthesis of 4-phenylquinazoline-2-carboxylic acid (2).

A

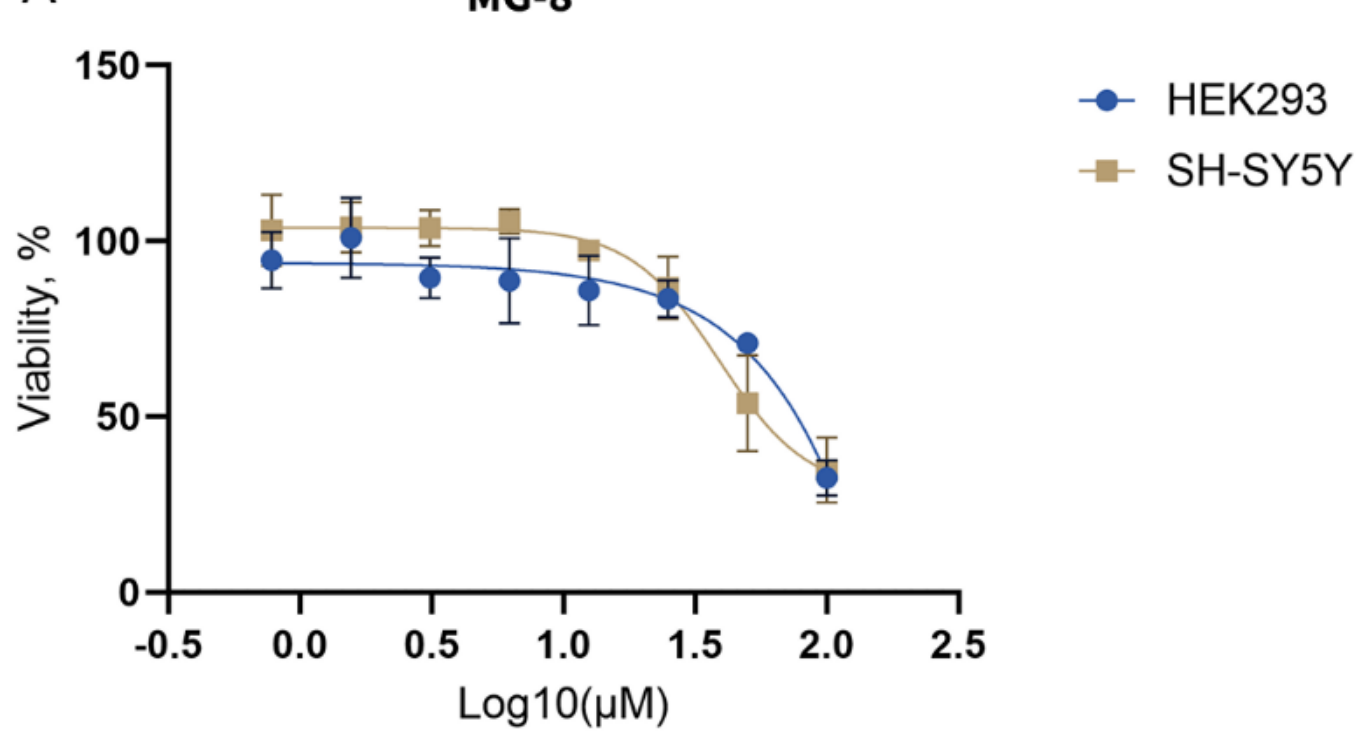

B

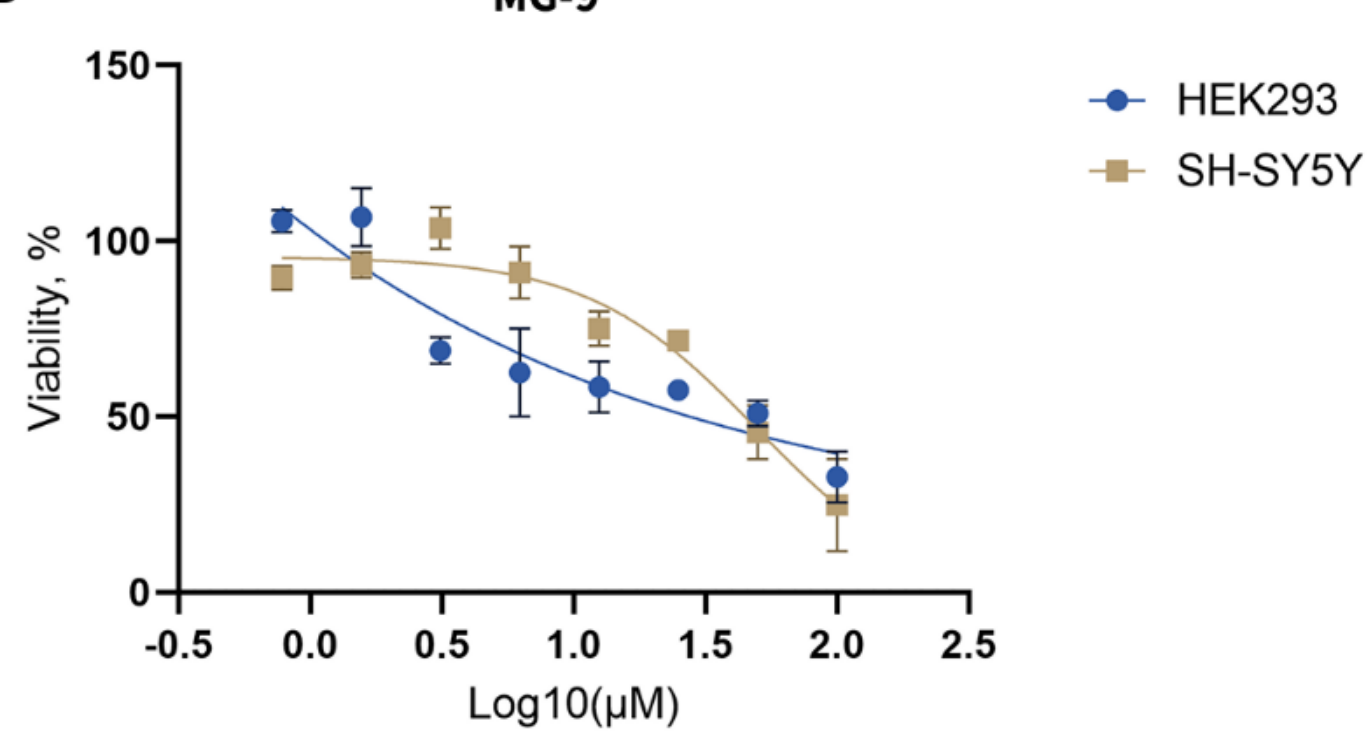

C

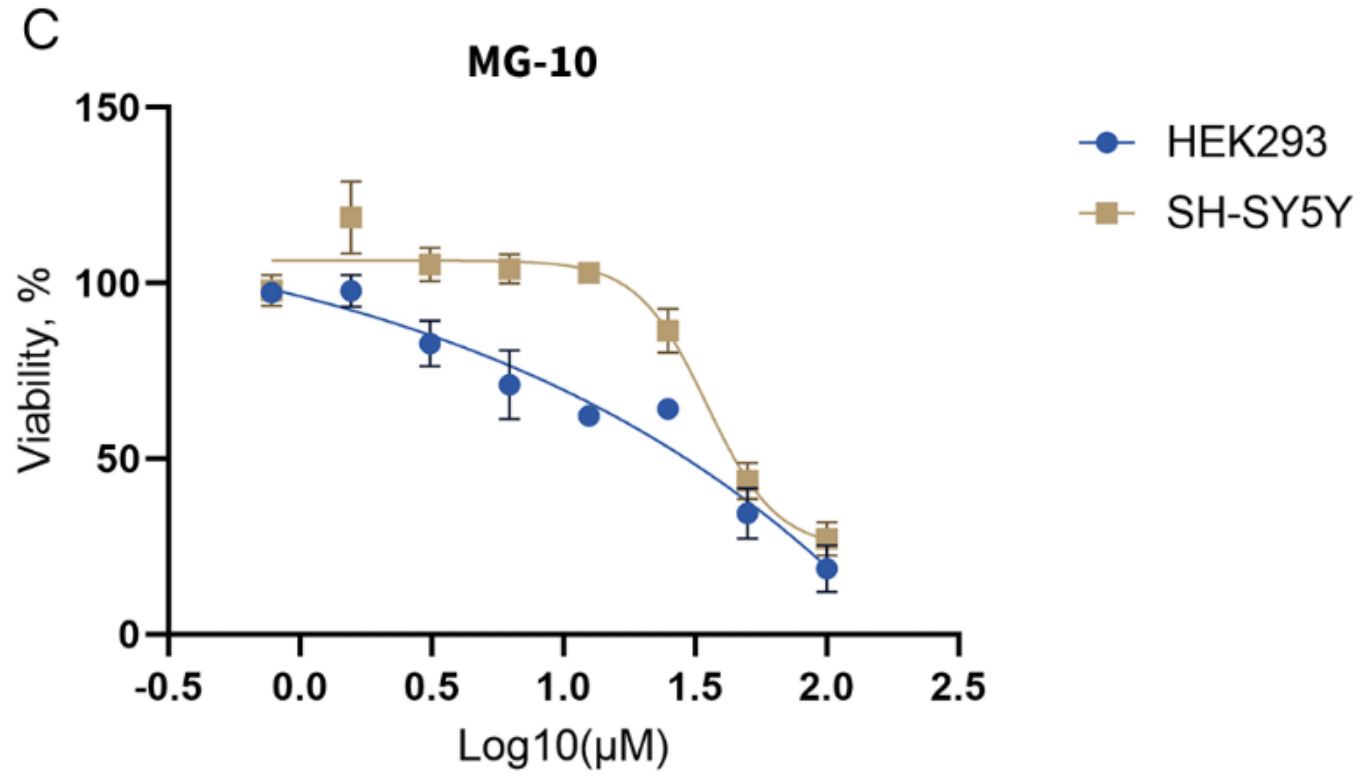

D

	CC_{50}, [μM]	
	**SH-SY5Y**	**HEK293**
**MG-1**	>100	>100
**MG-8**	39.3±5.8	76.7±11.3
**MG-9**	35.2±3.4	24.2±2.3
**MG-10**	60.1±6.5	24.5±5.1

E

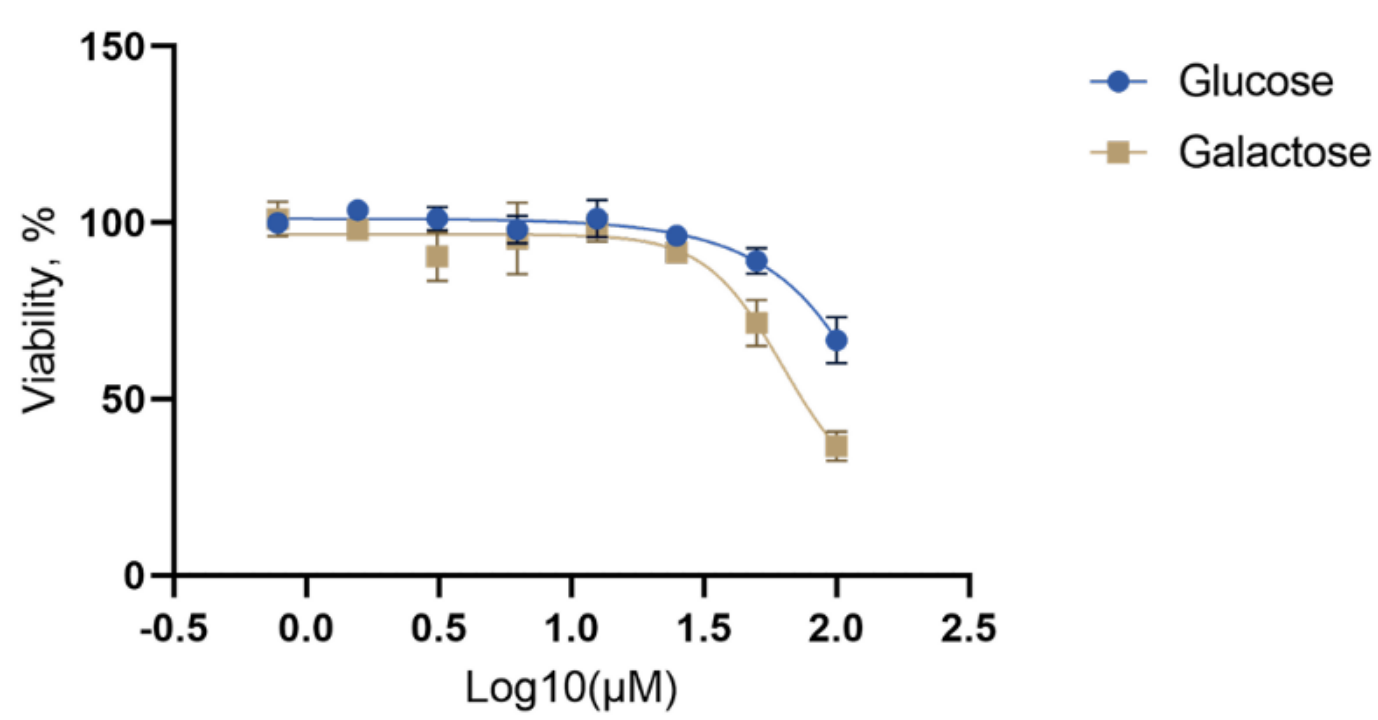

F

	CC_{50}, [μM]	
	**Glucose**	**Galactose**
**MG-1**	>100	74.7±12.1

Supplementary Figure S2. Evaluation of cytotoxicity of TSPO-based hybrids. Viability of human neuroblastoma (SH-SY5Y) and Human embryonic kidney (HEK293) incubated with **MG-8** (**A**), **MG-9** (**B**) or **MG-10** (**C**) for 24 hours. MTT assay, data are the mean ± SD. **D.** Table indicating CC_{50} values for compounds on different cell lines. **E.** Viability of human neuroblastoma (SH-SY5Y) and Human embryonic kidney (HEK293) cells treated with **MG-1** and cultured in glucose- or galactose-containing medium, as assessed by MTT assay. **F.** CC_{50} values for MG-1 under the indicated culture conditions.

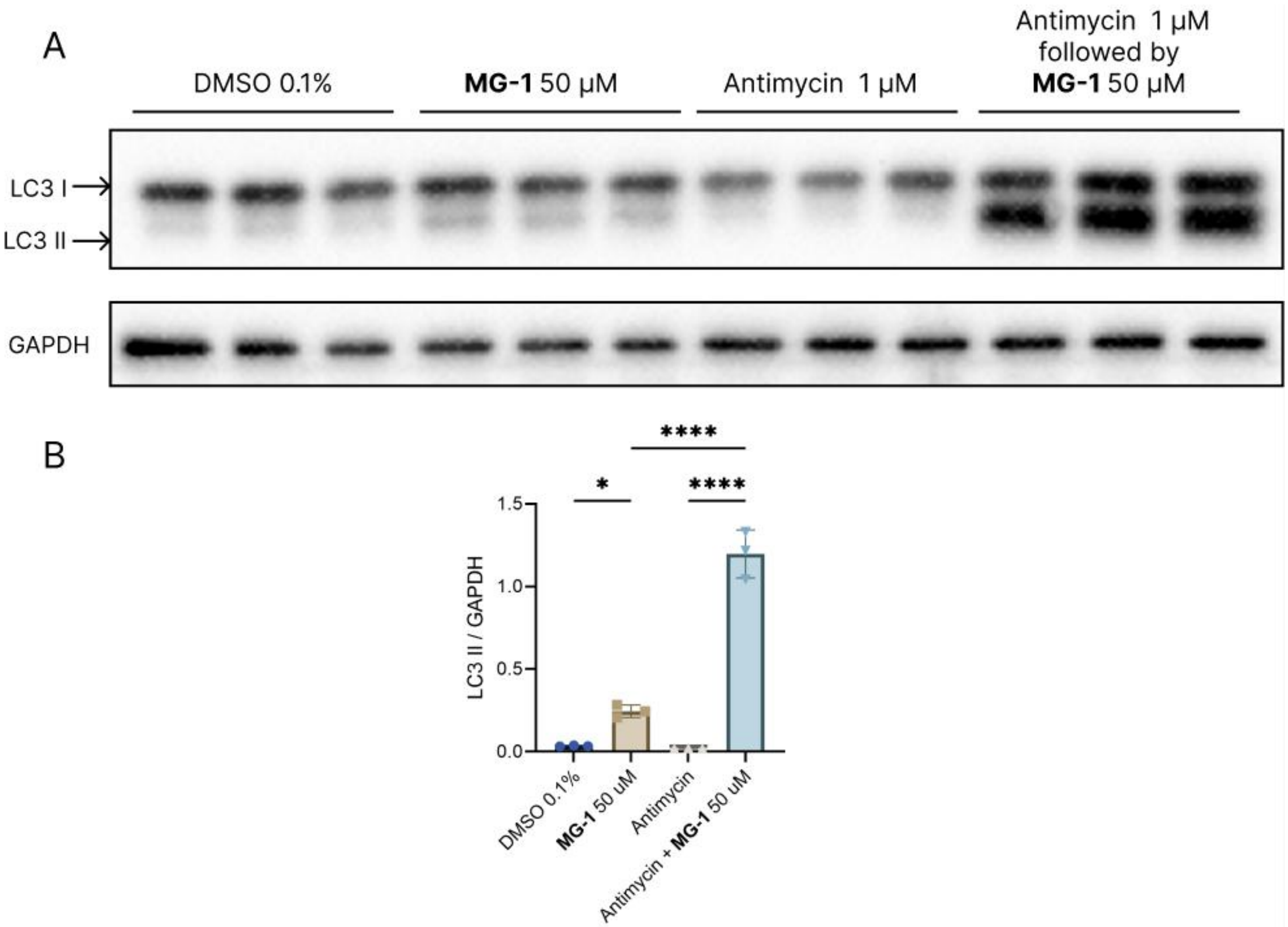

Supplementary Figure S3. Investigation of **MG-1** activity in oxidative stress conditions. A. Immunoblotting on LC3 in normal conditions and in oxidative stress, which was modeled by addition of 1 µM of antimycin for 4 hours. B. Bar graph showing the LC3-II level normalized on GAPDH. The results of T-tests are indicated on the graph: * – p-value < 0.05, **** – p-value < 0.0001.

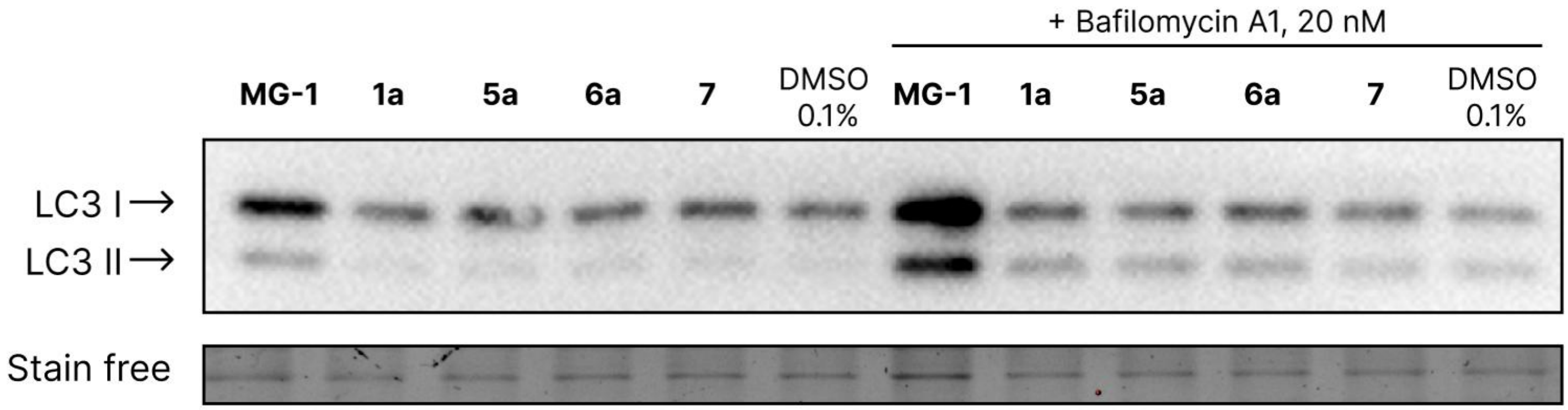

Supplementary Figure S4. Investigation of activation of autophagy flux by **MG-1** and its moieties. A. Immunoblotting on LC3 in normal conditions and in the presence of 20 nM of bafilomycin A1 (2 h). B. Bar graph showing the LC3-II level normalized on stain free. The results of Mann-Whitney tests are indicated on the graph: * – p-value = 0.05.

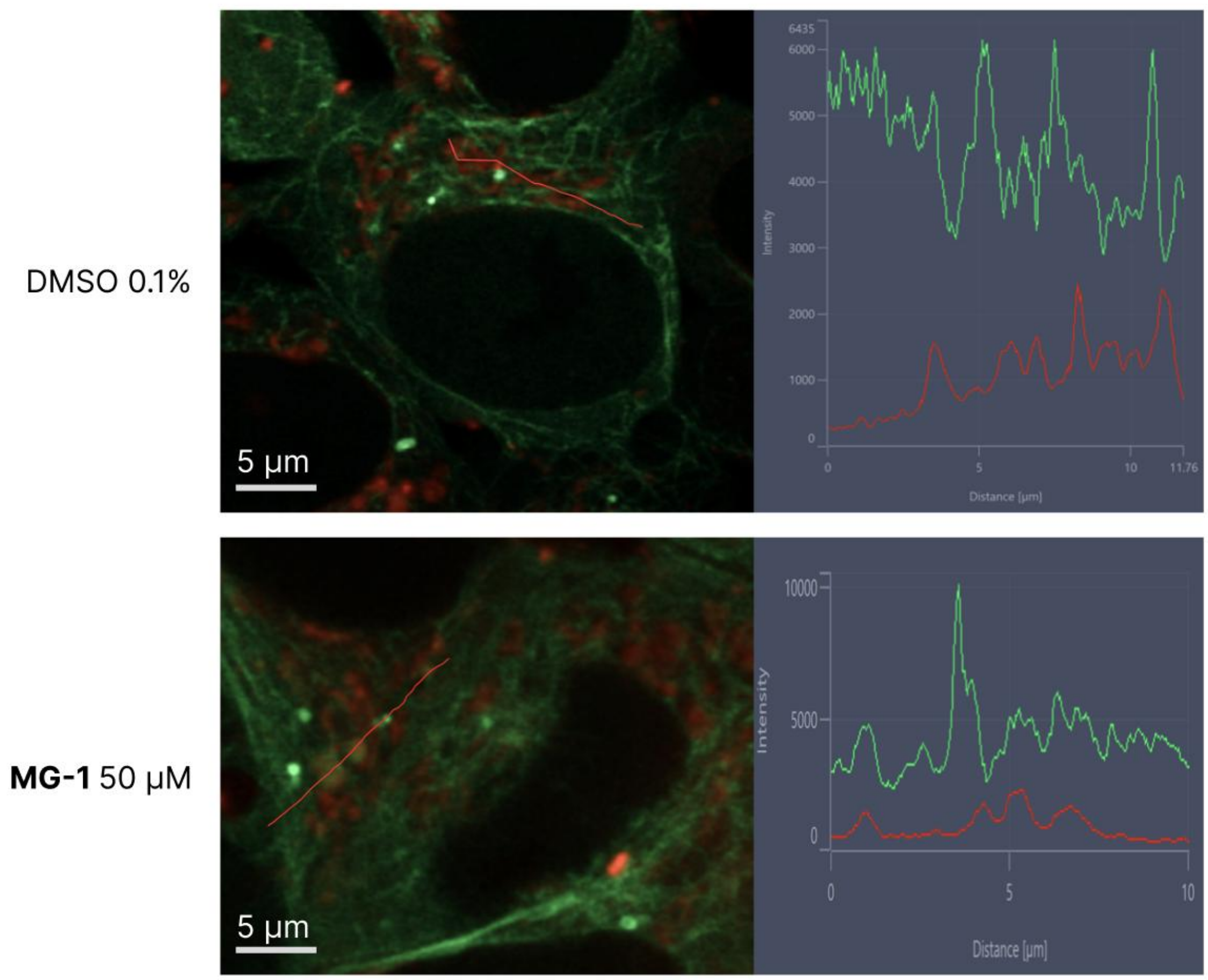

Supplementary Figure S5. Microphotographs of SH-SY5Y expressing LC3-GFP and stained with LumiTracker® Mito Red FM treated with MG-1 and control cells (left) and graph of green and far red fluorescence intensities across the line (right).

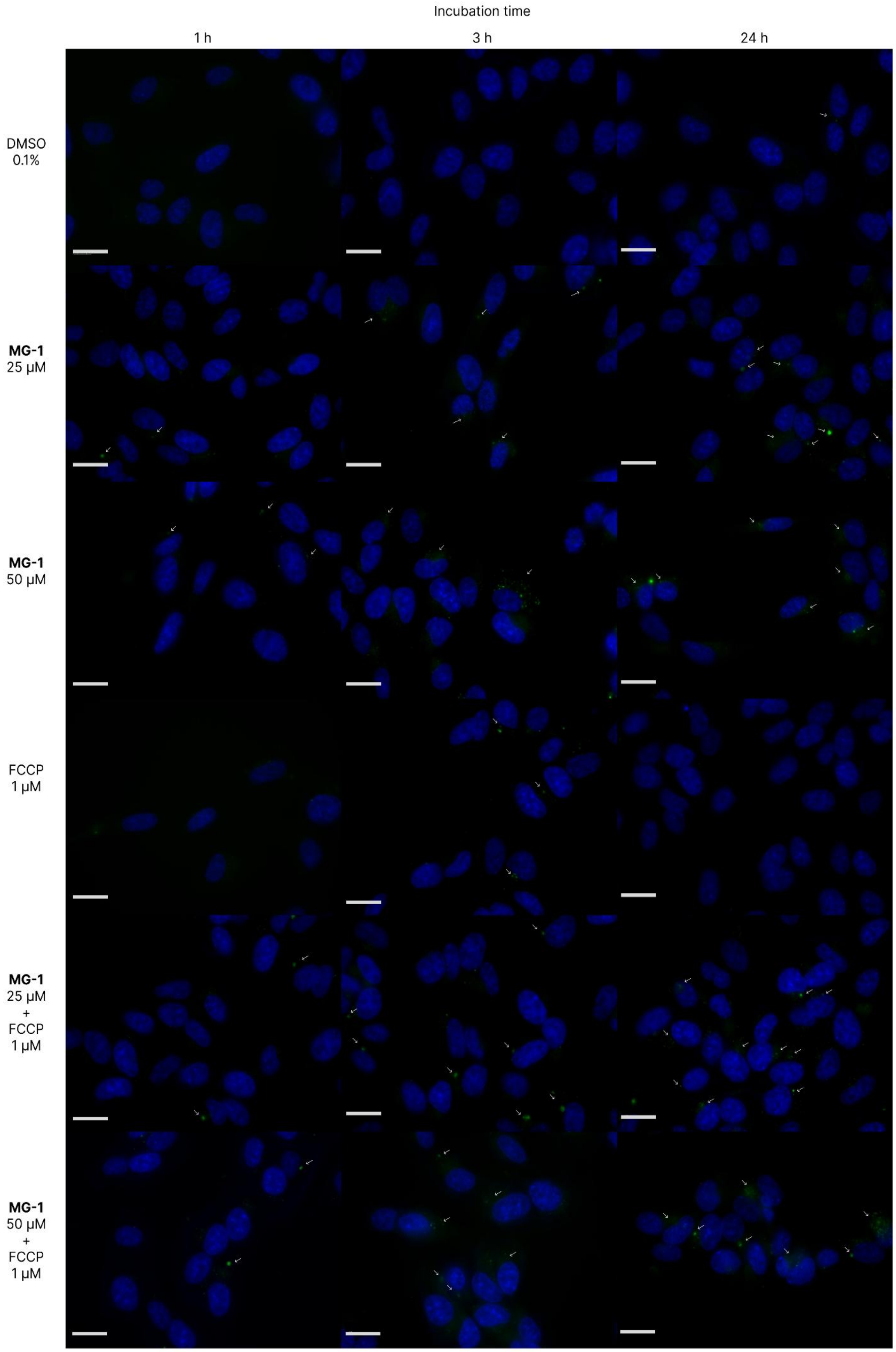

Supplementary Figure S6. Accumulation of lysosomes in wild type human neuroblastoma cell line SH-SY5Y upon 1, 3 or 24 hour treatment with **MG-1** alone or with **MG-1** in combination with FCCP 1 µM or with control compounds. White bars indicate 20 µm. White arrows mark lysosomes. Lysosomes were stained with Lysotracker (green), nuclei were stained with Hoechst (blue).

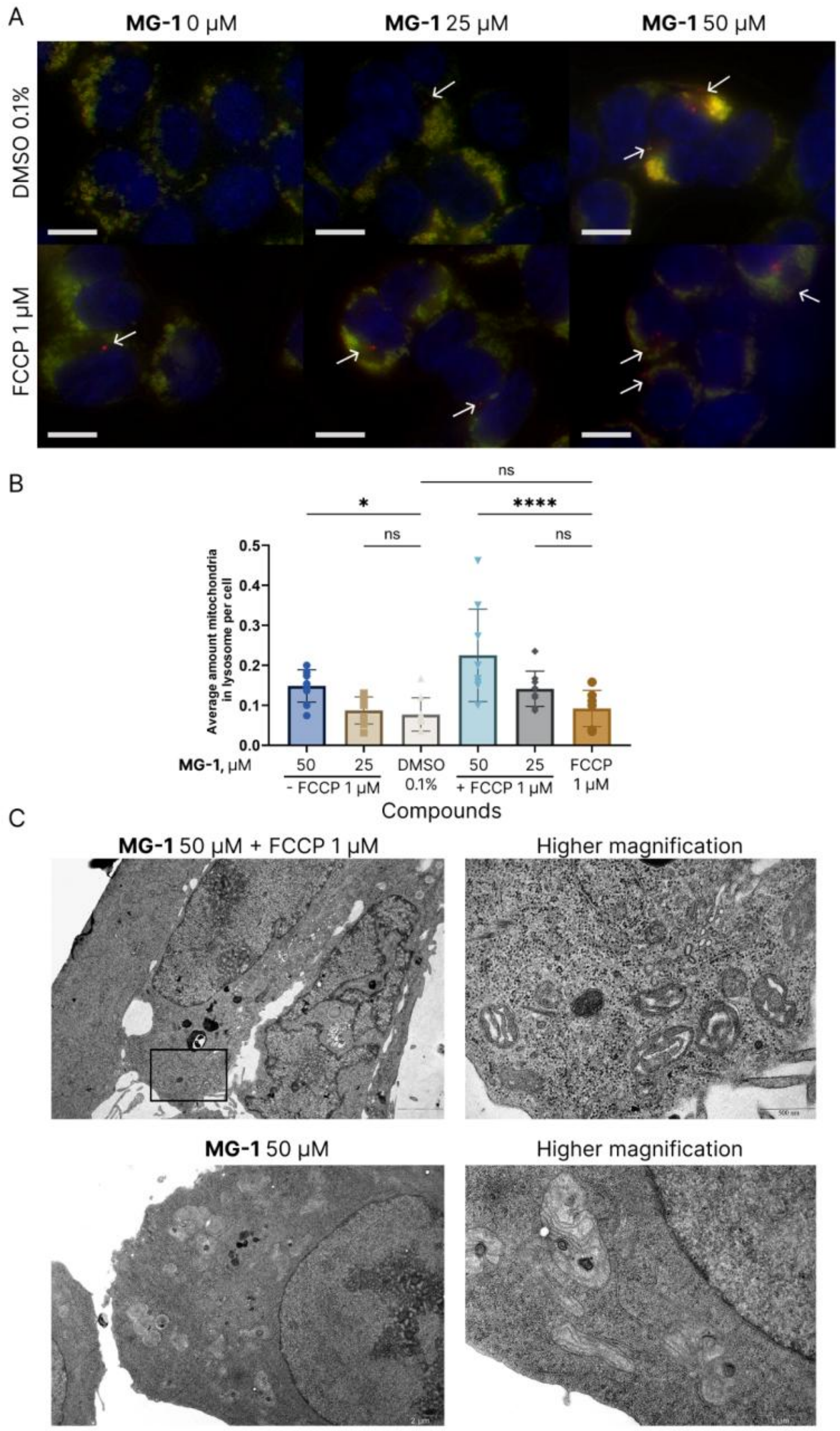

Supplementary Figure S7. MG-1 induce mitophagy in various cell lines. **A.** Mitophagy induced by **MG-1** in wild type HEK293T expressing TOM20MTS-mCherry-EGFP-Tet-On. White bars indicate 10 μm. White arrows mark mitochondria in lysosomes. Nuclei were stained with Hoechst (blue). Cells were treated with doxycycline (2 ug/mL) 24h before the experiment. **B.** Bar graph indicating average amount of cells HEK293T expressing TOM20MTS-mCherry-EGFP-Tet-On in which was induced mitophagy (n=9). The results of t-test are indicated on the graph: * – p-value < 0.05, **** – p-value < 0.0001. **C.** Electron microscopy of N2a cells after treatment with **MG-1** alone or in combination with FCCP 1 μM.

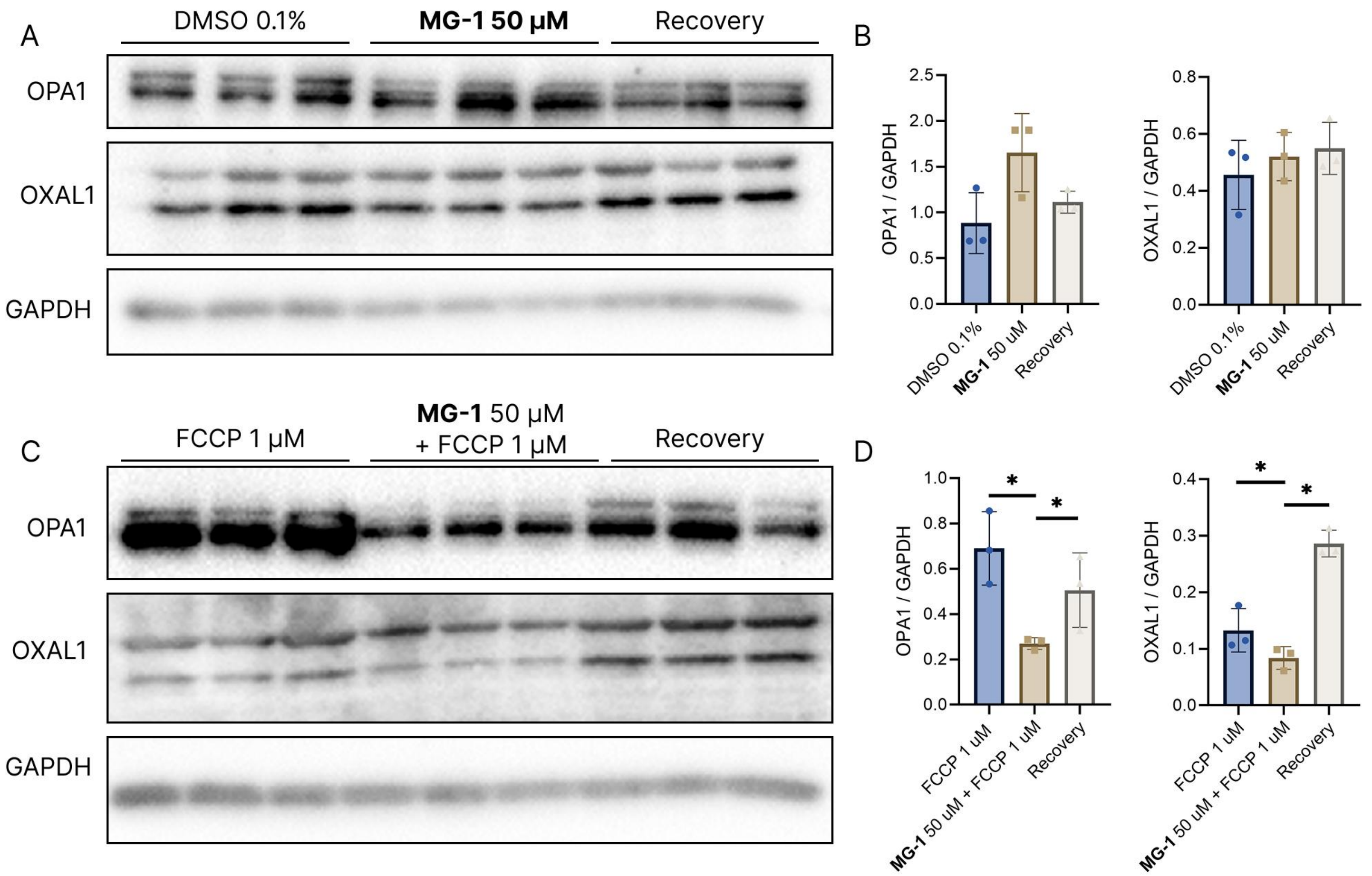

Supplementary Figure S8. Evaluation of levels of mitochondrial proteins upon treatment with **MG-1.** Immunoblotting with antibodies against OPA1 and OXAL1 from the wild type SH-SY5Y cells after treatment with MG-1 alone (**A**) and in combination with FCCP 1 μM (**C**) for 1, 24 hours or and 24 hours followed by recovery (rec.). **B, D**. Bar graphs representing relative values of OPA1 and OXAL1 normalized on GAPDH. Data represents mean ± SD (n=3). The results of Mann-Whitney test are indicated on the graph: * – p-value = 0.05.

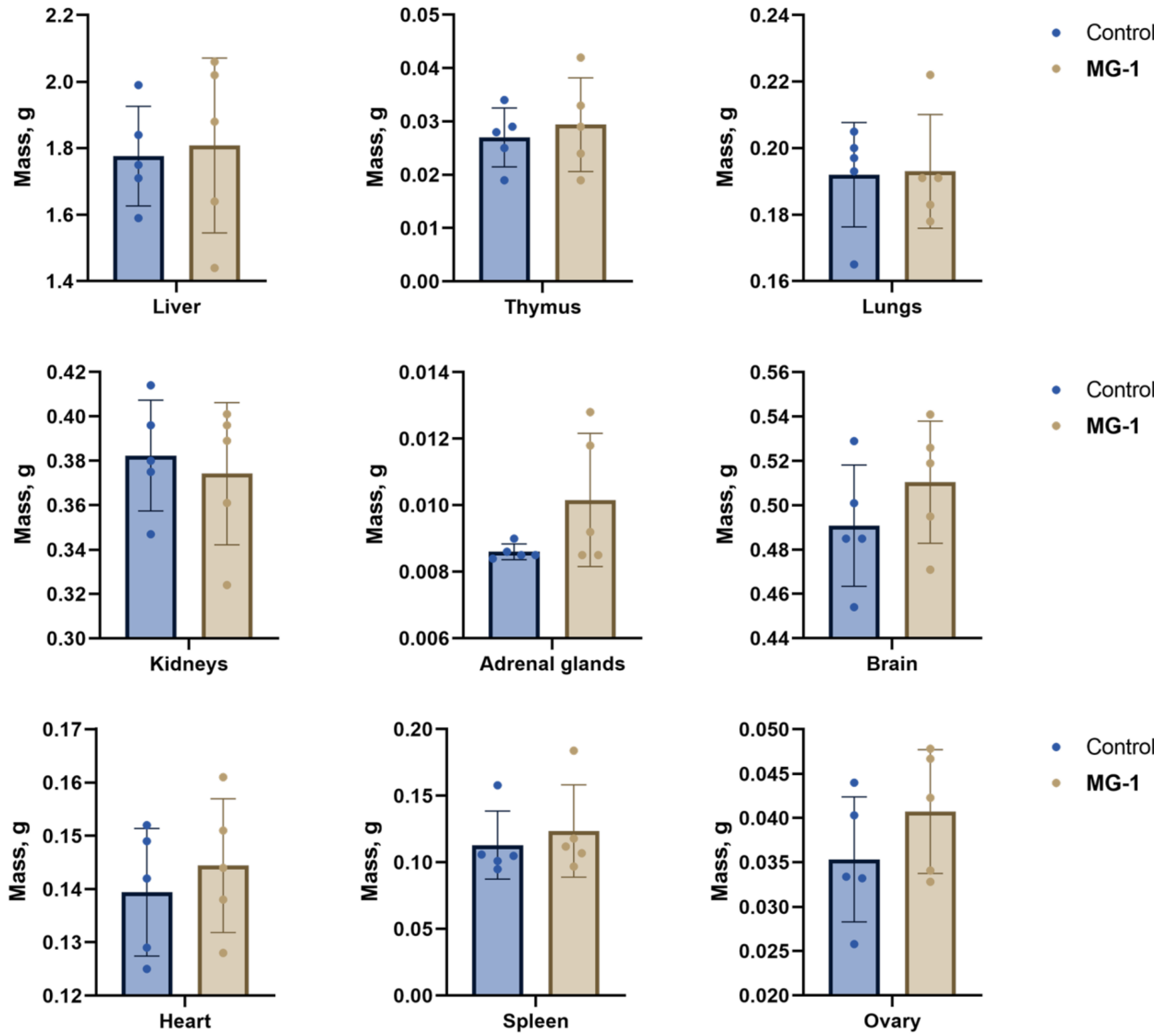

Supplementary Figure 9. Organ weights of mice 14 days after a single administration of **MG-1** at a dose of 1000 µg/g or vehicle (corn oil).

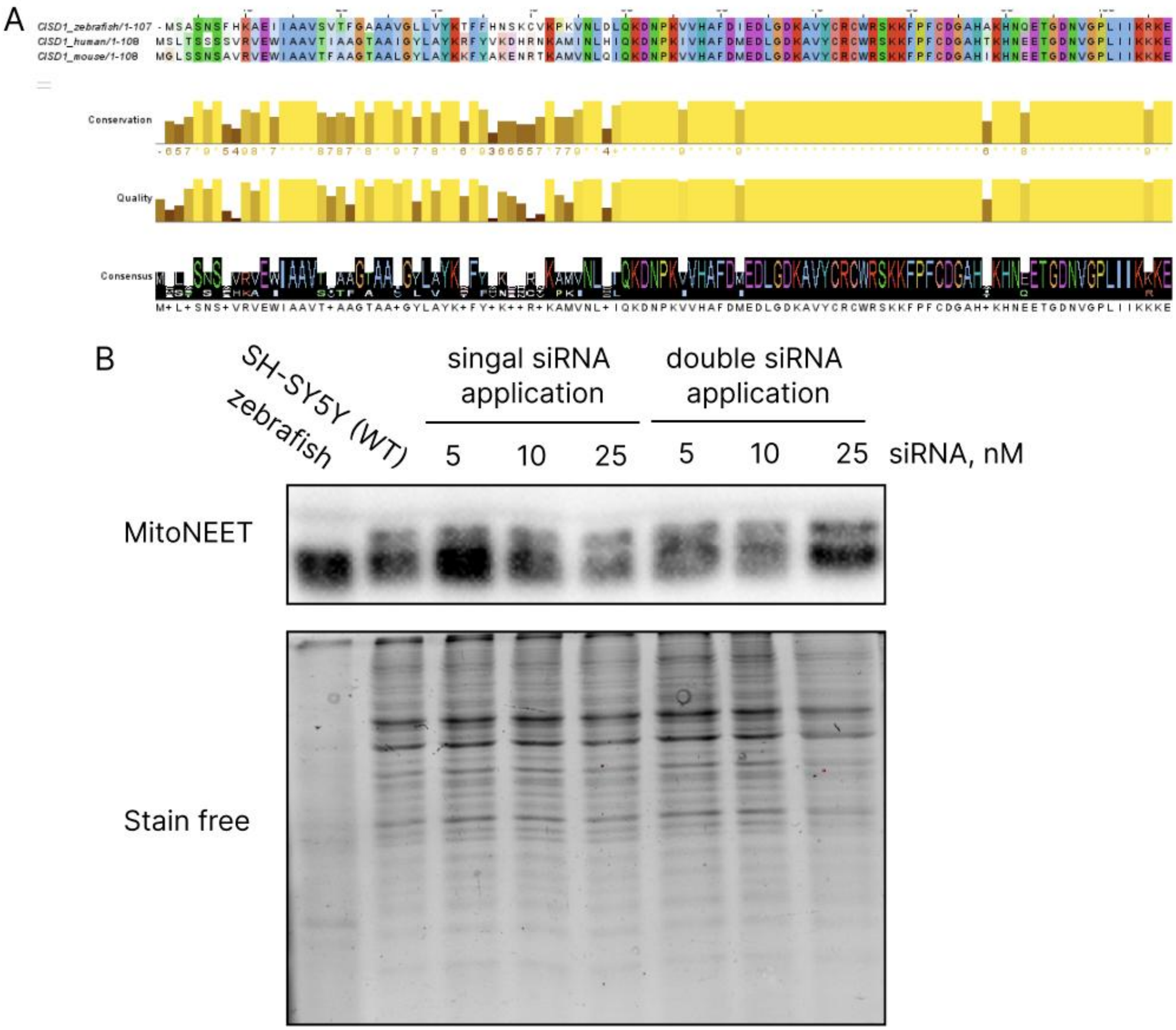

Supplementary Figure S10. MitoNEET is expressed in *Danio rerio* embryos. **A.** Multiple alignment of protein sequences of MitoNEET (CISD1) orthologs from human (NP_060934.1), mouse (NP_598768.1) and zebrafish (NP_956899.1). **B.** Immunoblotting with an anti-MitoNEET antibody and corresponding stain-free images of zebrafish embryos and human neuroblastoma (SH-SY5Y) cells treated with MitoNEET siRNAs or control cells.

Materials and methods

Generation and maintenance of mammalian cell lines

The human neuroblastoma (SH-SY5Y) and lung fibroblast (VA-13) cell lines were cultured in Dulbecco's modified Eagle medium (DMEM; ServiceBio, China), while the human T lymphocyte (Jurkat) cell line was cultured in RPMI medium (Gibco, USA). All cells were maintained at 37°C in a humidified atmosphere containing 5% CO_2. Both media were supplemented with 10% (v/v) fetal bovine serum (FBS), 2 mM HiGlutaXL (HiMedia, PA, USA), and 1× penicillin–streptomycin (HiMedia, PA, USA).

To assess the ability of **MG-1** to induce autophagy, we used an SH-SY5Y cell line stably expressing the fluorescent reporter system GFP-LC3-RFP[1–3]. For evaluation of mitophagy induction, we employed a monoclonal HEK293 cell line expressing the fluorescent reporter system TOM20MTS-mCherry-EGFP-Tet-On[4].

The reporter-expressing cell lines were generated by lentiviral transduction. Lentivirus was produced by transfecting HEK293T cells with either pMXs GFP-LC3-RFP (Addgene #117413, [3]) or TOM20MTS-mCherry-EGFP-Tet-On (Addgene #109016,[4]), together with the packaging plasmids pCMV-VSV-G (Addgene #8454) and psPAX2 (Addgene #12260), using Lipofectamine 3000 (Thermo Fisher Scientific, USA). Wild-type SH-SY5Y and HEK293T cells were then infected with the lentivirus, and stable transformants were isolated by fluorescence-activated cell sorting (BD FACSAria III), resulting in monoclonal cell lines.

To investigate the mechanism of action of **MG-1**, we used an SH-SY5Y cell line with inactivated *AMPK* and *SIRT1* genes, as previously described[1,2].

The activity of **MG-1** was further assessed in combination with the ULK1 inhibitor ULK101, which was added at a concentration of 10 µM ([5]; HY-114490, MedChem Express, USA), to test for activation of autophagy through ULK1-independent pathways.

For MitoNEET knockdown, SH-SY5Y cells were seeded in 24-well plates. ON-TARGETplus Human CISD1 siRNA (Horizon, UK) was prepared according to the manufacturer's instructions. The siRNA mixture was transfected into cells using Lipofectamine RNAiMAX (Invitrogen, USA) according to the manufacturer's recommendations, at a final duplex concentration ranging from 5 to 25 nM. Twenty-four hours after transfection, the knockdown cells were treated with the test compounds for an additional 24 h and subsequently subjected to immunoblotting analysis.

Autophagy assays

For autophagy activity screening, human neuroblastoma (SH-SY5Y) cells were seeded in 96-well plates (CCP-96H, ServiceBio, China) in DMEM medium (ServiceBio, China) at a density of 40,000 cells per well. After 24 h of incubation, cells were treated with the test compounds, either alone or in combination with a low concentration of FCCP (1 µM). Controls included 5 µM FCCP and 0.1% DMSO. Following a 24 h incubation, cells were processed for immunoblotting using anti-LC3 antibodies.

To induce mitochondrial oxidative stress, cells were treated with antimycin A (1 µM) for 4 h. The medium was then replaced with fresh medium containing either MG-1 or DMSO as a vehicle control.

For analysis of autophagic flux, cells were treated with the test compounds for 22 h, after which bafilomycin A1 (20 nM) was added for an additional 2 h without removing the test compounds.

To analyze the mechanism of autophagy induction, SH-SY5Y cells were seeded in 24-well plates (CCP-24H, ServiceBio, China) in DMEM medium at a density of 200,000 cells per well. After 24 h of incubation, cells were treated with test and control compounds. Following an additional 24 h incubation, cells were collected and processed for immunoblotting with antibodies against proteins involved in the autophagy induction cascade.

Immunoblotting

Immunoblotting of cell lysates and fish embryos was performed as previously described[6]. Briefly, SH-SY5Y cells or fish embryos were lysed in RIPA buffer (150 mM sodium chloride, 50 mM Tris-HCl, pH 8.0, 0.5% Nonidet P-40, 1% sodium deoxycholate, 0.5% SDS) supplemented with a protease inhibitor cocktail (Thermo Fisher Scientific, USA). Lysates were separated on 10–15% SDS-PAGE gels and transferred onto PVDF membranes (0.45 μm, Thermo Fisher Scientific, USA) using wet transfer. Membranes were blocked for 1 h at room temperature in TBST (10 mM Tris-HCl, pH 7.5, 150 mM NaCl, 0.1% Tween-20) containing 5% bovine serum albumin (BSA, Proliant Biologicals, USA).

Primary antibodies were diluted 1:1000 in TBST with 5% BSA. The following antibodies were used: Tuberin/TSC2 (3612, Cell Signaling Tech., USA), phospho-Tuberin/TSC2 (Ser1387) (5584, Cell Signaling Tech., USA), ULK1 (8054, Cell Signaling Tech., USA), phospho-ULK1 (Ser555) (5869, Cell Signaling Tech., USA), phospho-ULK1 (Ser317) (89267, Cell Signaling Tech., USA), AKT (9272, Cell Signaling Tech., USA), phospho-Akt (Thr308) (9275, Cell Signaling Tech., USA), AMPK (2532, Cell Signaling Tech., USA), phospho-AMPK (T172) (2535, Cell Signaling Tech., USA), 4E-BP1 (9644, Cell Signaling Tech., USA), phospho-4E-BP1 (Thr37/46) (236B4, Cell Signaling Tech., USA) , LC3 (4108, Cell Signaling Tech., USA), phospho-Ubiquitin (Ser65) (E2J6T, Cell Signaling Tech., USA), p62 (ab56416, Abcam, USA), Parkin (#2132, Cell Signaling Tech., USA), OPA1 (80471, Cell Signaling Tech., USA), OXA1L (66128-1-Ig, Proteintech, Germany), COX VIII (4850, Cell Signaling Tech., USA), MitoNEET (16006-1-AP, Proteintech, USA), VDAC (4661, Cell Signaling Tech., USA), GAPDH (1:3000, ab8245, Abcam, USA) were used as a loading control.

Secondary HRP-conjugated anti-rabbit (1706515, Biorad, USA) and anti-mouse (7076, Cell signaling, USA) antibodies were used at a 1:5000 dilution.

Pull-down assay

Mouse liver lysates were used to analyze the interactions of individual **MG-1** moieties with LC3 and MitoNEET. Whole mouse liver tissue was homogenized in 3 mL of lysis buffer containing 50 mM Tris-HCl (pH 7.5), 100 mM NaCl, and 0.5% NP-40, supplemented with a protease inhibitor cocktail (Thermo Fisher Scientific, USA). Sepharose beads containing the respective **MG-1** moiety, linker, or no compound (negative control) were incubated with 5 mg of liver lysate for MitoNEET analysis or 10 mg of liver lysate for LC3 analysis for 1 h at 4°C with constant mixing. The beads were subsequently washed three times with lysis buffer. Bound proteins were eluted by boiling the beads in 1× Laemmli sample buffer and analyzed by immunoblotting.

Fluorescent microscopy

To evaluate the activity of **MG-1** the SH-SY5Y expressing GFP-LC3-RFP reporter system was employed as previously described[1,2]. Briefly, reporter cells were seeded in 96-well plates (655866, Greiner Bio-One, Germany) in DMEM medium (ServiceBio, China) at a density of 20,000 cells per well. After 24 h of incubation, cells were treated with

MG-1 or **MG-1** in combination with FCCP (1 μM) or controls (5 μM FCCP and 0.1% DMSO). Following a further 24 h incubation, four micrographs per well were acquired at 10× magnification using a CELENA® X High Content Imaging System (Logos Biosystems, South Korea), provided by the Moscow State University Development Program. Image analysis was performed using CELENA X Cell Analyzer software to quantify the average RFP and GFP fluorescence intensity in individual cells. At least 500 cells were analyzed for each condition. Statistical significance of differences in RFP/GFP ratios between conditions was assessed using ANOVA.

For analysis of mitochondrial–LC3 colocalization following MG-1 treatment, cells expressing the pMXs GFP-LC3-RFP reporter system were seeded onto glass-bottom dishes (35 mm; ibidi GmbH, Germany) at a density of 1 × 10^5 cells per dish. After 24 h of incubation, cells were treated with MG-1 for an additional 24 h and subsequently stained with LumiTracker® Mito Red FM (Lumiprobe, Russia) according to the manufacturer's instructions. Micrographs were acquired using a Zeiss LSM900 confocal microscope equipped with an Airyscan 2 detector and a 63×/1.4 Plan-Apochromat objective. Image analysis was performed using ZEN (Blue Edition) 3.5 and Fiji 20250514.1117[7].

To evaluate the ability of **MG-1** to induce mitophagy was used HEK293T expressing TOM20MTS-mCherry-EGFP-Tet-On reporter system. Cells were treated with doxycycline (2 ug/mL, Terno Fisher Scientific, USA) 24 h before experiment. The seeding and treatment procedures were the same as written above. Microphotographs were acquired at 60× magnification using a CELENA® X High Content Imaging System (Logos Biosystems, South Korea). Image analysis was performed using Fiji 20250514.1117[7]

SH-SY5Y or MRC-5/SV40 fibroblasts stably expressing the tandem fluorescent reporter mCherry-GFP-Fis1 were seeded into 35-mm glass-bottom dishes (SPL Life Science, #100350) at a density of 1 × 10^5 cells per dish for confocal microscopy. After 24 h incubation with various concentrations of **MG-1**, primary human dermal fibroblasts were washed twice with PBS and stained with MitoTracker Green (200 nM, 15 min) and LysoTracker Red (150 nM, 15 min). Fluorescence of the mCherry-GFP-Fis1 tandem reporter in MRC-5/SV40 cells was analyzed without additional staining. Images were acquired using an Olympus IX83 fluorescence microscope (Tokyo, Japan)

Flow cytometry

Mitochondrial membrane potential (ΔΨm) changes in primary human dermal fibroblasts (Research Facility "Biobank," Research Centre for Medical Genetics, Moscow, Russia) were assessed using the potentiometric dye JC-1 (Lumiprobe, Moscow, Russia) and analyzed with an Amnis FlowSight® imaging flow cytometer (Luminex Corporation, Seattle, WA, USA). Cells were incubated with JC-1 at a final concentration of 1 μg/mL for 30 min at 37 °C, washed twice with warm culture medium, and immediately analyzed. The mitochondrial membrane potential was quantified as the ratio of red (aggregated JC-1; excitation 560 nm, emission 595 nm) to green fluorescence (monomeric JC-1; excitation 488–505 nm, emission 530–560 nm).

A decrease in the red/green fluorescence ratio, calculated using the IDEAS software (Luminex Corporation, Seattle, WA, USA), reflected mitochondrial depolarization.

DNA and RNA extraction, RT PCR

Assessment of the mitochondrial-to-nuclear DNA ratio was done as previously described[6]. DNA was extracted from 10^6 cells with LumiSpin® UNI DNA Isolation Spin Kit for Any Sample (Lumiprobe, Russia).

For the measurement of TFAM and PGC-1α levels total RNA was extracted from *Danio rerio* embryos or cell culture treated with **MG-1** in different conditions. RNA purification with RNA extract reagent (Evrogen)was followed by cDNA synthesis with a Maxima First Strand cDNA Synthesis Kit for RT-qPCR (Thermofisher) with a random hexamer primer.

Quantitative PCR gene amplifications were performed using SYBR® Green PCR master mix (ThermoFisher Scientific, Waltham, MA, USA) in the CFX384 Touch Real-Time PCR System. qPCR was conducted using primer sets: 1) preGAPDH 5'- CCACCAACTGCTTAGCACC-3', 5'-CTCCCCACCTTGAAAGGAAAT-3', and 12S 5'-AAACCCCGATCAACCTCACC-3', 5'-TTTACGTGGGTACTTgCgCT-3'; 2) human ([8]): *PGC-1a* (CACACTTAAGGTGCGTTCAATAGTC and CCAAACCAACAACTTTATCTCTTCC), *TFAM* (AGCTCATGGACTTCTGCCAGCA and CCTGCCTCCATAATATAAGGAAACAAGAGT), *18S* (TACCACATCCAAGGAAGGCAGCA and CTGCAGCAACTTTAATATACGCTAT); 3) *Danio rerio* ([9]): *Pgc-1α* (ACCAACCATCTTgCCACTTC and ATTACTCAGCCTGGGCCTTT), *Tfam* (GCGAAAGATTGCCCAGCAGT and TTGTCGTTTTTCCTCCGCAAA), *Rpl13* (TCTGGAGGACTGTAAGAGGTATGC and AGACGCACAATCTTGAGAGCAG). The amount of mitochondrial DNA and relative mRNAs was calculated by the 2-ΔΔCT method and normalized to the nuclear DNA (mtDNA), *18S* (human factors of biogenesis) or *Rpl13* (*Danio rerio* factors of biogenesis).

Electron microscopy

Mouse neuroblastoma cells (N2a) were grown on glass slides in a standard cell medium. Cells were exposed to the **MG-1** or **MG-1** in combination with FCCP (1 μM) or to the controls (1 μM FCCP or 0.1% DMSO). After 24 hours incubation the culture medium was removed, and the slides containing cells were fixed with 3% glutaraldehyde (Sigma Aldrich, St. Louis, MO, USA) in phosphate buffer with pH 7.4 for 2 hours at 4°C, followed by post-fixation with 1% osmium tetroxide in buffer with pH 7.4 for 1.5 hours. The cells were then dehydrated in alcohol solutions with increasing concentrations of alcohol (the 70% alcohol solution was saturated with uranyl acetate (Serva, Germany) to enhance the contrast of the samples). The material was poured into Epon-812 epoxy resin. Serial ultra-thin sections were made using a diamond knife on a Leica ULTRACUT UCT ultramicrotome (Germany). The study was acquired using a JEM-1400 electron microscope (Jeol, Japan).

***In vitro* cytotoxicity assay**

Human neuroblastoma SH-SY5Y cells, Human embryonic kidney HEK293, immortalized human lung fibroblasts VA-13 and human T lymphocyte Jurkat were seeded at a density of 20,000 cells per 100 μl DMEM/F12 medium per well in 96-well plates. The maximum **MG-1, MG-8, MG-9, MG-10** concentration used was 100 μM, and the final DMSO concentration did not exceed 0.5% (v/v).

Cell viability was assessed using the MTT assay after 24 h of compound treatment. Cells were incubated with MTT reagent (0.5 g/l; Paneco LLC, Russia) for 2 h at 37 °C in a CO_2 incubator. After incubation, the medium was removed, and 140 μl of DMSO was added to each well. Plates were incubated for at least 15 min at room temperature on an orbital shaker to solubilize formazan crystals. Absorbance was measured at 545 nm using a VICTOR X5 plate reader (PerkinElmer, USA).

Cytotoxicity data were analyzed using nonlinear regression in GraphPad Prism 9, applying a four-parameter logistic model (lower limit, upper limit, slope, and CC_{50}).

Absorbance and fluorescence spectra

Absorbance spectra were measured with Specord 50 spectrophotometer (Analytik, Jena, Germany). Fluorescence spectra were measured with a CM 2203 spectrophotometer (SOLAR, Minsk, Belarus).

Lifespan assay on *C. elegans* model

The wild-type *Caenorhabditis elegans* N2 Bristol strain (The Swammerdam Institute of Life Sciences, University of Amsterdam, Netherlands) was used to assess the effects of **MG-1** compound on nematode lifespan. The *Escherichia coli* OP50 strain (Engelhard Institute of Molecular Biology, Moscow, Russia) was used as a food source.

Lifespan assays were performed using synchronized L4-stage adult nematodes. Synchronization was carried out as described previously[10]. Briefly, 120 µl of synchronized L1 larvae were dispensed into 96-well plates (TPP, Switzerland). After 48 h, 15 µl of 5-fluoro-2′-deoxyuridine (FUDR; Merck, Germany) was added to each well to inhibit progeny production. After a further 24 h, 15 µl of **MG-1** solution was added to obtain final concentrations of 500, 250, 100, 50, 10, and 1 µg/ml.

Each concentration was tested in six replicates. Lifespan extension efficacy was evaluated relative to untreated controls. Survival data were analyzed using GraphPad Prism (GraphPad Software, LLC, USA) with Kaplan–Meier survival analysis.

Mice husbandry

The study used sexually mature healthy female mice of ICR SPF (specific microflora free) status. Animals were obtained from the Pushchino nursery of laboratory animals (Pushchino, Russia). All procedures and manipulations with mice were approved by the Committee for Control over Care and Use of Laboratory Animals of BIBCh RAS (IACUC) (protocol number 1004/24 from 04.11.2024) and were carried out in accordance with the EU Directive 2010/63/EU. After receiving the animals from the nursery, they were placed for a week of adaptation, during which their health was monitored using a clinical examination. Animals without signs of pathology were selected into experimental groups using body weight as a criterion so that it did not differ between the groups. For identification, the animals were marked on the auricles using a puncture method. During the study, the animals were kept in controlled conditions of the barrier zone (temperature 20-24 °C, relative humidity 30-55%, 12 h light cycle; 08:00-20:00- “day”, 20:00-08:00- “night”; 10-fold change in air volume in the room per hour). The animals received ad libitum food for laboratory mice and rats Velaz FORTI 1324 Maintenance Diet (Altromin Spezialfutter GmbH & Co KG, Im Seelenkamp 20, D-32791 Lage, Germany) and filtered water *ad libitum*.

Maximum tolerated dose finding on mice

The animals were divided into two groups: a control group, which was administered the vehicle - corn oil, and a group with the introduction of **MG-1**. The study was carried out in accordance with the OECD Guideline 420[11] using fixed dose procedure, according to which the initial dose for administration is 1000 µg/g body weight, administered to one female, which is observed for 48 hours. The female showed no signs of toxicity and the next four females were given a dose of 1000

μg/g body weight. The animals were observed for 14 days, recording body weight and weight gain on days 1 (before administration), 3, 7 and 14 after administration, and performing clinical examinations and health assessments on these days. On day 15 after administration, all surviving animals were euthanized and necropsied. Animals were euthanized by anesthesia with a mixture of Telazol® (tiletamine + zolazepam) + Xylazine, followed by exanguination. During necropsy, animals were examined for body cavities and organs (ovaries, lungs, kidneys, adrenal glands, liver, heart, thymus, and brain) were weighed.

Danio rerio housing and breading

Danio rerio (zebrafish) were obtained from a local supplier (AquaCrab Store, Moscow) and housed in aquaria equipped with a UV filtration system and constant aeration. Each 70 L aquarium (Russia, MSU facility) contained 70-90 individuals. Adults were maintained on a 14 h/10 h light-dark cycle, fed with TetraMin flakes (Tetra GmbH, Germany), and kept at room temperature (22-26 °C). Water temperature was maintained at 28 ± 0.8 °C using a ZebTEC Benchtop system (Tecniplast), with pH between 7.0 and 7.5 and conductivity between 530 and 610 mS. Water was changed weekly, and light intensity was maintained at 200 lux.

Sexually mature zebrafish (4-6 months old) of both sexes were used for spawning. Males and females were transferred to separate breeding tanks in a 2:1 or 1:1 ratio a few hours before the dark phase on the day prior to the experiment. At least three breeding tanks were set up in parallel. Eggs were collected from the bottom of the tanks, rinsed with water, and briefly incubated (30-40 s) in 1% methylene blue solution to prevent fungal contamination. Unfertilized or damaged eggs were excluded from further experiments. To minimize genetic variability, eggs for experimental procedures were collected from at least three parental groups, pooled, and randomly distributed among test conditions.

Acute toxicyty on embryo of *Danio rerio*

To assess the acute toxicity of autophagy inducers, *Danio rerio* embryos at the 16-cell stage were exposed to solutions of the test compounds. Each experimental group contained at least 10 randomly selected embryos (OECD Test No. 236:2013, modified). Within 90 min post-fertilization, embryos were transferred into 24-well plates (CCP-24N, ServiceBio, China) containing either 2 mL of test compound solution per well or water used for compound dilution (negative control). Test and control solutions were renewed daily.

Embryonic development was monitored over 24-120 h post-fertilization, and major abnormalities were recorded, including egg coagulation, absence of somite formation, failure of tail detachment, and lack of heartbeat.

Based on embryo viability, the following parameters were determined: maximum tolerated dose, minimum lethal concentration (100% mortality), cumulative mortality at each concentration, and LC_{50} values with 95% confidence intervals. Statistical analyses were performed using the Mantel-Cox survival test in GraphPad Prism 9 (GraphPad Software, USA).

MPTP model of Parkinson disease in *Danio rerio*

The dopaminergic system in zebrafish develops within the first 96 h after hatching[12,13]. To induce Parkinson's-like symptoms, embryos were exposed to 1-methyl-4-phenyl-1,2,3,6-tetrahydropyridine (MPTP, 5 μg/ml; Moscow State University, Russia).

To evaluate the efficacy of **MG-1**, embryos were maintained in one of the following conditions: **MG-1** alone, **MG-1** with MPTP, **MG-1** alone with recovery, **MG-1** with MPTP with recovery, MPTP alone (positive control), or water (negative control). Treatments began at 24 h post-fertilization and continued for 3 days. Recovery groups were subsequently maintained for an additional 24 hours in fresh water without MG-1 and/or MPTP. Each experimental group consisted of 10 embryos maintained in 6-well plates with 5 ml of solution per well. Water and test solutions were renewed daily. Embryos were monitored daily, and survival was recorded.

Behavioral testing was performed on day 6 of development using the Noldus EthoVision XT video tracking system (Wageningen, Netherlands) in an open-field test. Individual fry were placed in wells of a 24-well plate containing 2 ml of water. After a 10-min acclimation period, locomotor activity was recorded for 5 min. Parameters analyzed included total distance traveled, average speed, and duration of immobility.

Statistical analyses were performed using GraphPad Prism 9 (GraphPad Software, USA). Survival curves were analyzed with the Mantel–Cox test, and behavioral parameters were compared using two-way ANOVA. Differences were considered statistically significant at $p < 0.05$.

After behavioral testing embryos were collected and freeze (-80°C) for RNA and protein analysis.

MitoNEET ortholog analysis

The MitoNEET ortholog sequences from *Homo sapiens*, *Mus musculus*, and *Danio rerio* were obtained from the NCBI database under the following accession numbers: NP_060934.1, NP_598768.1, and NP_956899.1, respectively. Multiple sequence alignment was performed using Jalview (version 2.11.5.2) [14] with the MUSCLE algorithm and default parameters [15].

Synthetic Procedures

All reagents, salts and solvents were of highest purity and used as supplied by Macklin, AmBead, Aldrich and Acros. Purity of the compounds was checked by thin layer chromatography using silica-gel 60 F_{254}-coated Al plates (Merck) and spots were observed under UV light. NMR spectra were recorded on a Bruker AVANCE 600 spectrometer (600.13 MHz) at 25°C temperature in $CDCl_3$ or DMSO-d_6. The chemical shifts are reported in parts per million (ppm) using DMSO-d_6 or $CDCl_3$ as an internal standard; the coupling constants expressed in Hz. The following abbreviations were used for spin multiplicity: s = singlet, d = doublet, t = triplet, dd = double doublet, m = multiplet. All spectra were processed with MestReNova 14.2.2 (Suppl. Fig. S11-36).

All reactions were carried out under an air atmosphere unless otherwise stated. Anhydrous solvents were used as received or dried according to standard procedures. Reaction progress was monitored by TLC on silica gel. Products were purified by recrystallization or column chromatography as indicated.

Synthesis of substituted benzaldehydes (General method A)

A solution of *p*-hydroxybenzaldehyde derivative (10 mmol, 1.0 equiv) in dry DMF (5 mL) was treated with potassium carbonate (20 mmol, 2.0 equiv, fine powder) followed by *tert*-butyl bromoacetate (12 mmol, 1.2 equiv). The reaction mixture was stirred at 80 °C for 3 h. After completion, the mixture was poured into ice–water (50 mL) and stirred

for 30 min. The precipitate was collected by filtration and washed with *n*-hexane (2 × 10 mL) to remove residual *tert*-butyl bromoacetate. The product was obtained as an off-white powder.

Synthesis of benzylidene-thiazolidinedione derivatives (General method B)

Substituted benzaldehyde (10 mmol, 1.0 equiv), thiazolidinedione (10 mmol, 1.0 equiv), and piperidine (1 mmol, 0.1 equiv) were mixed in toluene (50 mL) and refluxed under Dean–Stark conditions for 6 h with continuous removal of water. The reaction mixture turned yellow during the process. After cooling to room temperature, the solid product was filtered off and washed with cold toluene. The crude yellow powder was obtained, if necessary, recrystallized from a small amount of ethanol.

Synthesis of free carboxylic acids 1a-c (General method C)

Tert-butyl esters (1 mmol) were dissolved in dry dichloromethane (10 mL), and trifluoroacetic acid (5 mL) was added dropwise. After 10 min, a precipitate began to form. The reaction mixture was stirred for 1 h and then evaporated to dryness. The residue was used in subsequent reactions without further purification.

Synthesis of isatoic anhydrides 3a-b (General method D)

Substituted isatin (60 mmol, 1.0 equiv) was dissolved in formic acid (40 mL), and hydrogen peroxide (37% w/w, 120 mmol, 2.0 equiv) was added dropwise under stirring. After ~10 min, an exothermic reaction commenced with the color changed from orange to yellow. After reaching 55 °C the flask was immediately placed in an ice bath, and an additional portion of hydrogen peroxide (37% w/w, 60 mmol, 1.0 equiv) was added. After 30 min, the precipitated product was filtered, washed with methanol (2x30 mL), and dried to afford a beige powder (Yield 3a 65% and 3b 61%).

Synthesis of N-alkyl isatoic anhydrides 4a-c (General method E)

Substituted isatoic anhydrides (3a,b; 40 mmol, 1.0 equiv) were dissolved in anhydrous DMF (40 mL). Sodium hydride (60% dispersion in mineral oil, 50 mmol, 1.2 equiv) was added portionwise. After hydrogen evolution ceased (~5 min), alkyl bromide (50 mmol, 1.2 equiv) was added. The reaction mixture was stirred at room temperature for 1 h and then poured into water (200 mL). The precipitate was stirred for 30 min, filtered, and washed with water (50 mL) and *n*-hexane (2 × 50 mL) to give N-alkyl isatoic anhydrides 4a–c as beige powders.

Synthesis of methyl esters 5a-c (General method F)

Dimethyl malonate (60 mmol, 2.0 equiv) was dissolved in dry DMF (40 mL), and sodium hydride (60% dispersion in mineral oil, 66 mmol, 2.2 equiv) was added carefully. After hydrogen evolution ceased, N-alkyl isatoic anhydride (4a-c; 30 mmol, 1.0 equiv) was added. The reaction mixture was stirred at 80 °C for 2 h, cooled, and poured into ice-water (200 mL). The solution was neutralized with 1M HCl to pH 7, affording a beige precipitate. The solid was filtered, washed with *n*-hexane (2 × 50 mL), and dried to give methyl esters 5a-c as beige powders.

Synthesis of amines 6a-c (General method G)

Methyl esters 5a-c (3 mmol, 1.0 equiv) and linker amine (3 mmol, 1.0 equiv) were mixed in toluene (20 mL) and refluxed for 5 h under stirring. The reaction mixture darkened during the process. After solvent removal under reduced pressure, the residue was dissolved in dichloromethane (10 mL), treated with 4 N HCl in dioxane (10 mL), and stirred for 30 min. The solvent was evaporated, and the residue was purified by column chromatography (CH_2Cl_2/MeOH, 0-15%) to afford hydrochlorides of amines 6a-c as yellow glassy solids.

Peptide coupling to give MG1-10 derivatives (General method H)

Hydrochlorides of amines 6a-c (2 mmol, 1.0 equiv), carboxylic acids 1a-c,2 (2 mmol, 1.0 equiv), and DIPEA (6 mmol, 3.0 equiv) were dissolved in dry DMF (5 mL). HATU (2.2 mmol, 1.1 equiv) was added, resulting in a yellow coloration. After stirring for 1 h, the mixture was diluted with water (50 mL), and the aqueous phase was decanted. The residue was dissolved in dichloromethane and purified by column chromatography (CH_2Cl_2/MeOH, 0-4%) to afford the final products MG1-10.

Preparation of Ligand-Immobilized Sepharose samples

CNBr-activated Sepharose 4 Fast Flow was prepared and subsequently coupled with an amine-containing ligand according to a modified procedure based on the manufacturer's protocol. All operations involving the activated matrix prior to ligand coupling were performed at 5–10 °C to minimize hydrolysis of the reactive groups.

A 4% cross-linked agarose (Sepharose 4 Fast Flow) was washed with distilled water and suction dried under reduced pressure. The resulting powder (1.0 mL) was suspended in 1.0 mL of cold potassium phosphate buffer A (3.33 M K_3PO_4, 1.56 M KH_2PO_4) and diluted with distilled water to a total volume of 2.0 mL. A solution of cyanogen bromide (100 mg/mL, 400 µL, 40 mg CNBr) was added portionwise over 2 min. The suspension was gently mixed at 5–10 °C for exactly 10 min, with the reaction time measured from the beginning of CNBr addition. The activated medium was then rapidly washed on a sintered filter with cold distilled water until the filtrate reached neutral pH. The activated Sepharose was used for ligand coupling in the same day.

The ligand was dissolved at a concentration of 10 µmol/mL in coupling buffer B (0.1 M $NaHCO_3$ and 0.5 M NaCl, pH 8.3). When required to dissolve the ligand, DMF was added to the coupling solution at a concentration not exceeding 50% (v/v). For coupling, 1.5 mL of the ligand solution was added per 1.0 mL of activated Sepharose. In a typical experiment, the ligand hydrochloride was dissolved in DMF (500 µL), followed by addition of coupling buffer B (1.0 mL). The resulting solution was combined with the activated Sepharose and gently mixed end-over-end for 1 h at room temperature. Magnetic stirring was avoided to prevent mechanical damage to the agarose beads.

Following coupling, the medium was washed with at least five volumes of coupling buffer B to remove unbound ligand. Residual reactive groups were subsequently blocked by incubation with buffer C (0.1 M Tris-HCl, pH 8.0), for 2 h at room temperature. The coupled medium was then subjected to three cycles of alternating-pH washing. Each cycle consisted of washing with at least five bed volumes of buffer D (0.1 M acetate buffer,

pH 4.0, 0.5 M NaCl), followed by washing with at least five bed volumes of buffer E (0.1 M Tris-HCl, pH 8.0, 0.5 M NaCl).

After suction drying under reduced pressure the resulting ligand-functionalized Sepharose was stored as a suspension in 20% (v/v) aqueous ethanol at 4 °C until use.

tert-butyl 2-(4-formylphenoxy)acetate

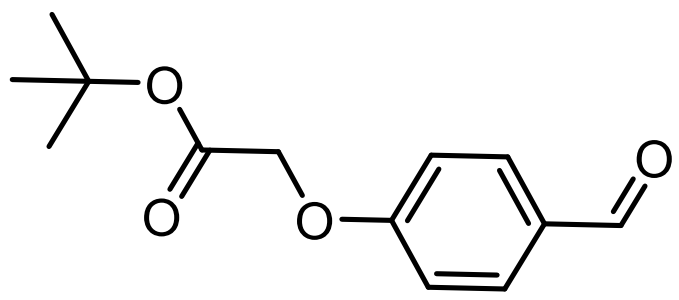

Compound was prepared according to General method A. Yield 86%

^{1}H NMR (600 MHz, $CDCl_3$) δ = 9.9 (s, 1H), 7.9 – 7.8 (m, 2H), 7.0 – 6.9 (m, 2H), 4.6 (s, 2H), 1.5 (s, 9H).

Spectral data in according with previously described [16]

tert-butyl 2-(4-formyl-2-methoxyphenoxy)acetate

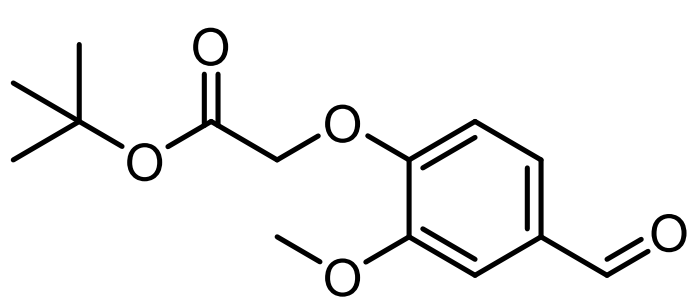

Compound was prepared according to General method A. Yield 92%

^{1}H NMR (600 MHz, $CDCl_3$) δ = 9.8 (s, 1H), 7.3 (s, 2H), 6.8 (d, *J*=8.9, 1H), 4.6 (s, 2H), 3.9 (s, 3H), 1.4 (s, 9H).

^{13}C NMR (151 MHz, $CDCl_3$) δ = 190.7, 167.0, 152.6, 149.8, 130.8, 126.0, 112.0, 109.8, 82.7, 66.0, 56.0, 27.9.

Spectral data in according with previously described [17]

tert-butyl (Z)-2-(4-((2,4-dioxothiazolidin-5-ylidene)methyl)phenoxy)acetate

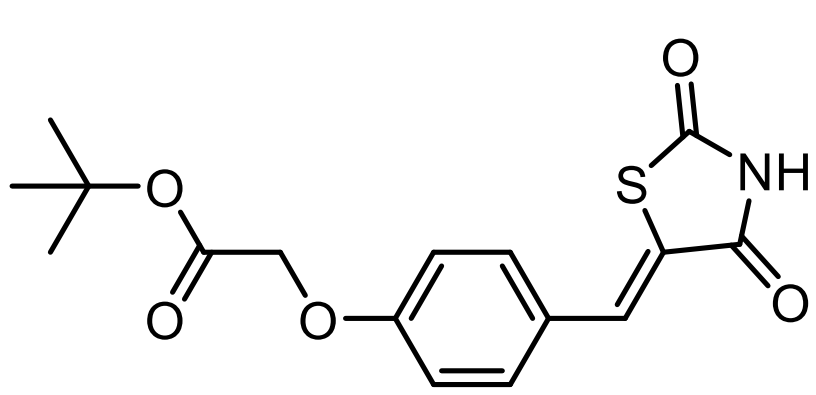

Compound was prepared according to General method B. Yield 68%

^{1}H NMR (600 MHz, $CDCl_3$) δ = 7.8 (s, 1H), 7.5 – 7.4 (m, 2H), 7.0 – 7.0 (m, 2H), 4.6 (s, 2H), 1.5 (s, 9H).

tert-butyl (Z)-2-(4-((2,4-dioxothiazolidin-5-ylidene)methyl)-2-methoxyphenoxy)acetate

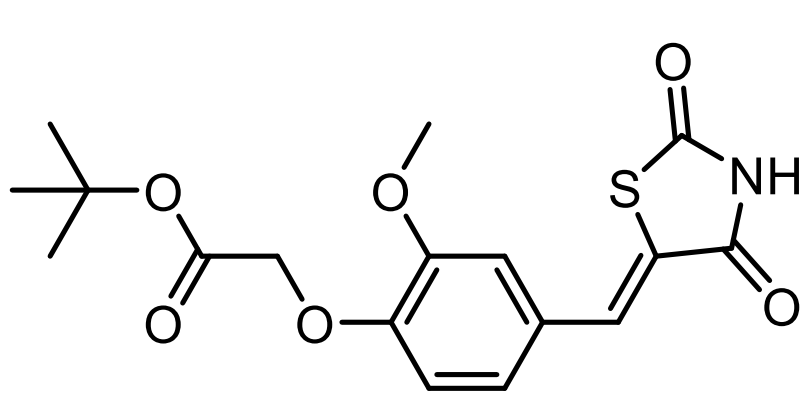

Compound was prepared according to General method B. Yield 51%

^{1}H NMR (600 MHz, DMSO) δ = 12.5 (s, 1H), 7.7 (s, 1H), 7.2 (s, 1H), 7.1 (d, *J*=8.5, 1H), 7.0 (d, *J*=8.5, 1H), 4.7 (s, 2H), 3.8 (s, 3H), 1.4 (s, 9H).

(Z)-2-(4-((2,4-dioxothiazolidin-5-ylidene)methyl)phenoxy)acetic acid (1a)

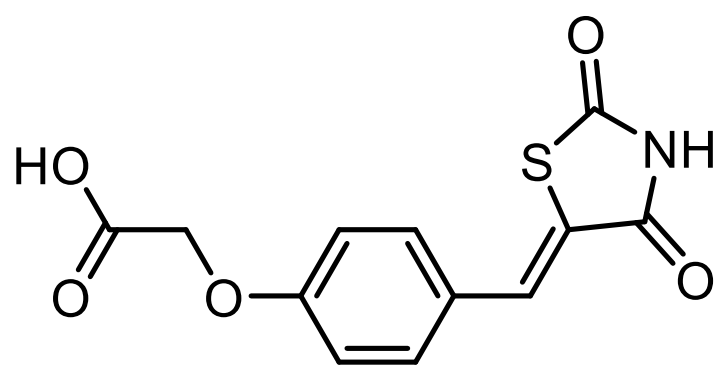

Compound was prepared according to General method C. Quantitative yield.

^{1}H NMR (600 MHz, DMSO) δ = 7.8 (s, 1H), 7.6 – 7.5 (m, 2H), 7.1 – 7.1 (m, 2H), 4.8 (s, 2H). **^{13}C NMR** (151 MHz, DMSO) δ = 169.2, 167.3, 166.8, 158.8, 131.4, 131.1, 125.4, 120.0, 114.8, 63.9.

Spectral data in according with previously described [18]

(Z)-2-(4-((2,4-dioxothiazolidin-5-ylidene)methyl)-2-methoxyphenoxy)acetic acid (1b)

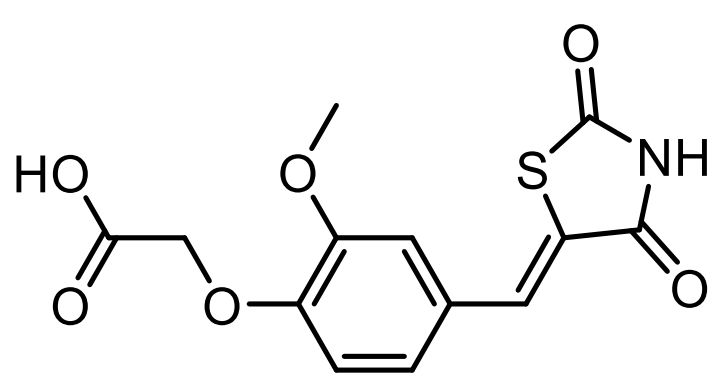

Compound was prepared according to General method C. Quantitative yield.

^{1}H NMR (600 MHz, DMSO) δ = 12.5 (s, 1H), 7.7 (s, 1H), 7.2 (d, *J*=2.1, 1H), 7.1 (dd, *J*=8.5, 2.1, 1H), 7.0 (d, *J*=8.5, 1H), 4.8 (s, 2H). **^{13}C NMR** (151 MHz, DMSO) δ = 169.8, 168.0, 167.4, 149.2, 149.0, 132.1, 126.3, 123.3, 120.8, 113.8, 113.2, 64.8, 55.7.

(Z)-2-(2,6-dibromo-4-((2,4-dioxothiazolidin-5-ylidene)methyl)phenoxy)acetic acid (1c)

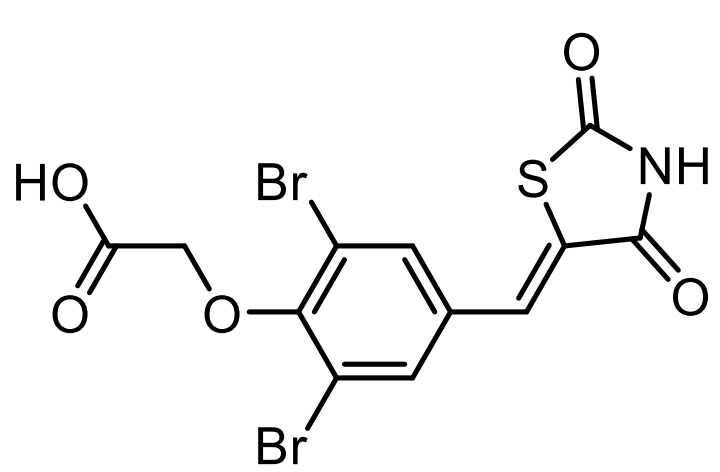

Compound was prepared according to General method C. Quantitative yield.

^{1}H NMR (600 MHz, DMSO) δ = 13.2 (s, 1H), 12.7 (s, 1H), 7.9 (s, 2H), 7.7 (s, 1H), 4.6 (s, 2H). **^{13}C NMR** (151 MHz, DMSO) δ = 168.5, 167.1, 166.9, 152.6, 133.8, 132.6, 128.3, 125.8, 118.2, 68.8.

4-phenylquinazoline-2-carboxylic acid (2)

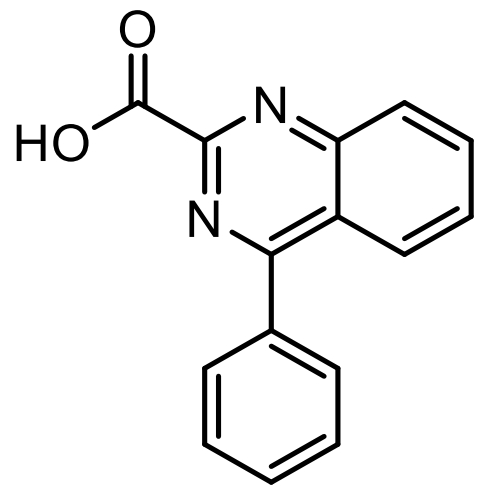

The compound was synthesized following the previously reported procedure. The obtained ^{1}H NMR spectrum was consistent with the data reported earlier [19]. Aminobenzophenone (7.0 g, 35.5 mmol, 1.0 equiv) was dissolved in ethanol (100 mL), and a 50% aqueous solution of glyoxylic acid (5.26 g, 35.5 mmol, 1.0 equiv) and ammonium acetate (8.21 g, 106.5 mmol, 3.0 equiv) were added with stirring. The reaction mixture was stirred at room temperature for 30 min and diluted with water (100 mL). The precipitate was filtered, washed with ethanol (20 mL) and diethyl ether (20 mL), affording an orange intermediate. The solid was dissolved in DMF (50 mL) and stirred in air under illumination from a tungsten lamp for 24 h. The mixture was poured into ice–water (100 mL), and the resulting beige solid was filtered and recrystallized from an acetonitrile/water (8:3, 20 mL) mixture to give the target product (6.85 g, yield: 77%).

^{1}H NMR (600 MHz, DMSO) δ = 8.2 (dd, *J*=8.5, 1.2, 1H), 8.2 – 8.1 (m, 2H), 7.9 (ddd, *J*=8.3, 6.9, 1.3, 1H), 7.8 – 7.8 (m, 2H), 7.7 – 7.6 (m, 3H). **^{13}C NMR** (151 MHz, $CDCl_3$) δ = 169.9, 162.9, 151.2, 150.3, 135.9, 135.3, 131.0, 130.3, 130.0, 129.0, 127.6, 123.5.

Spectral data in according with previously described [19]

1-ethyl-2H-benzo[d][1,3]oxazine-2,4(1H)-dione (4a)

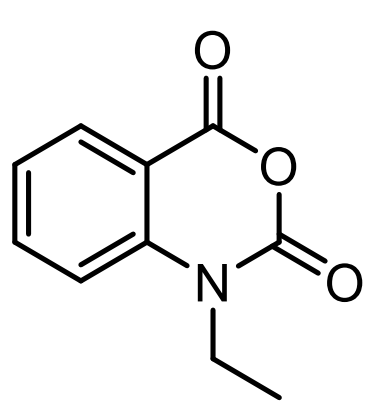

Compound was prepared according to General method E. Yield 60%

^{1}H NMR (600 MHz, $CDCl_3$) δ = 8.1 (dd, *J*=7.9, 1.7, 1H), 7.7 (ddd, *J*=8.7, 7.3, 1.7, 1H), 7.3 (t, *J*=7.6, 1H), 7.2 (d, *J*=8.4, 1H), 4.1 (q, *J*=7.2, 2H), 1.3 (t, *J*=7.2, 3H).

Spectral data in according with previously described [20]

7-chloro-1-ethyl-2H-benzo[d][1,3]oxazine-2,4(1H)-dione (4b)

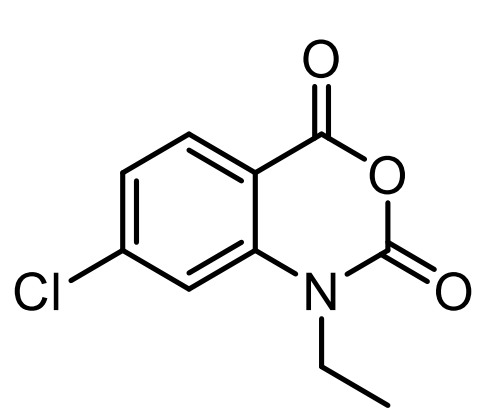

Compound was prepared according to General method E. Yield 57%

¹H NMR (600 MHz, $CDCl_3$) δ = 8.1 (d, *J*=8.4, 1H), 7.3 (d, *J*=8.4, 1H), 7.2 (s, 1H), 4.1 (q, *J*=7.2, 2H), 1.4 (t, *J*=7.2, 3H).

Spectral data in according with previously described [21]

methyl 1-ethyl-4-hydroxy-2-oxo-1,2-dihydroquinoline-3-carboxylate (5a)

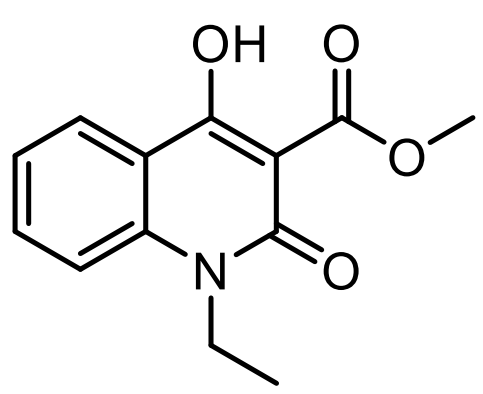

Compound was prepared according to General method F. Yield 55%

¹H NMR (600 MHz, $CDCl_3$) δ = 8.1 (dd, *J*=8.1, 1.6, 1H), 7.6 (ddd, *J*=8.7, 7.1, 1.7, 1H), 7.3 (d, *J*=8.5, 1H), 7.2 – 7.2 (m, 1H), 4.2 (q, *J*=7.1, 2H), 4.0 (s, 3H), 1.3 (t, *J*=7.2, 3H). **¹³C NMR** (151 MHz, $CDCl_3$) δ = 173.2, 171.8, 159.3, 140.6, 134.6, 126.1, 121.9, 115.1, 114.1, 97.9, 53.1, 37.4, 12.8.

Spectral data in according with previously described [20]

methyl 7-chloro-1-ethyl-2,4-dioxo-1,2,3,4-tetrahydroquinoline-3-carboxylate (5b)

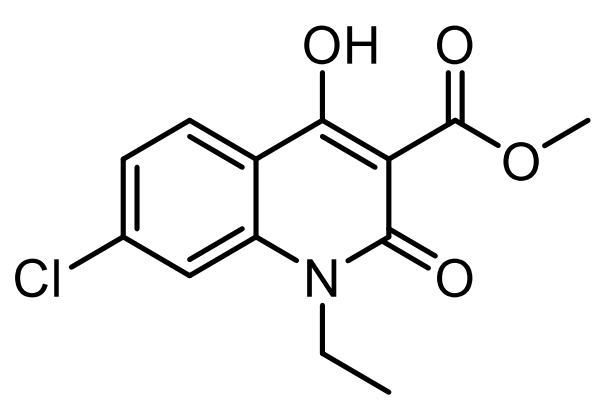

Compound was prepared according to General method F. Yield 54%

¹H NMR (600 MHz, $CDCl_3$) δ = 7.8 (d, *J*=8.6, 1H), 7.1 (s, 1H), 6.9 (d, *J*=8.6, 1H), 4.0 (q, *J*=7.2, 2H), 3.8 (s, 3H), 1.1 (t, *J*=7.1, 3H). **¹³C NMR** (151 MHz, $CDCl_3$) δ = 172.9, 171.1, 159.1, 141.2, 141.0, 127.4, 122.4, 114.0, 113.5, 97.7, 53.1, 37.6, 12.7.

methyl 1-benzyl-4-hydroxy-2-oxo-1,2-dihydroquinoline-3-carboxylate (5c)

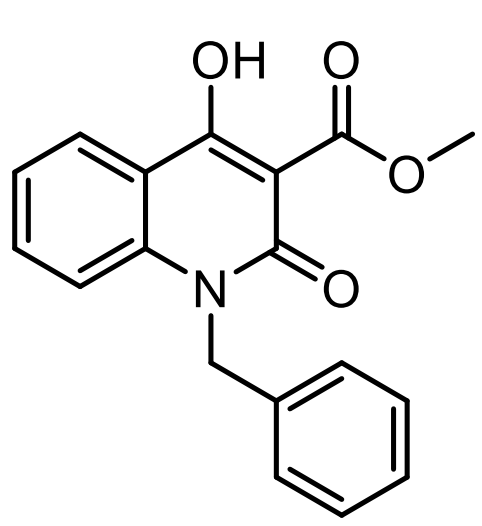

Compound was prepared according to General method F. Yield 50%

^{1}H NMR (600 MHz, DMSO) δ = 8.1 (d, *J*=7.9, 1H), 7.6 (t, *J*=7.9, 1H), 7.3 (d, *J*=8.6, 1H), 7.3 – 7.2 (m, 3H), 7.2 – 7.2 (m, 3H), 5.4 (s, 2H), 3.9 (s, 3H). **^{13}C NMR** (151 MHz, DMSO) δ = 169.5, 165.8, 158.9, 139.8, 136.9, 133.9, 128.6, 127.0, 126.5, 124.8, 122.1, 115.3, 114.7, 100.9, 52.5, 44.4.

Spectral data in according with previously described [22]

N-(3-(2-(2-(3-aminopropoxy)ethoxy)ethoxy)propyl)-1-ethyl-4-hydroxy-2-oxo-1,2-dihydroquinoline-3-carboxamide hydrochloride (6a)

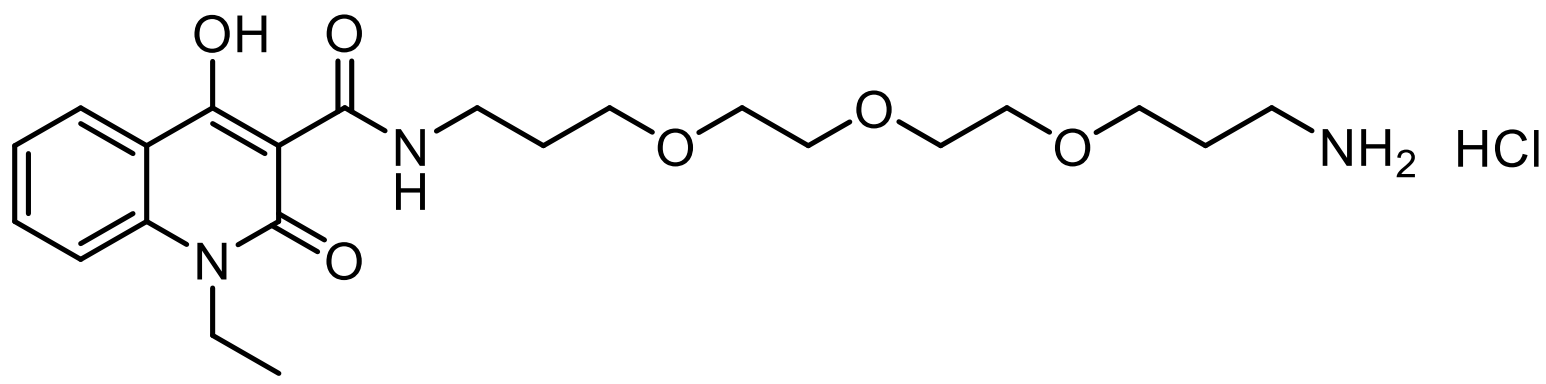

Compound was prepared according to General method G. Yield 91%

^{1}H NMR (600 MHz, $CDCl_3$) δ = 10.4 (t, *J*=5.8, 1H), 8.2 (dd, *J*=8.0, 1.6, 1H), 8.0 (bs, 3H), 7.7 (ddd, *J*=8.7, 7.1, 1.7, 1H), 7.4 (d, *J*=8.6, 1H), 7.3 (t, *J*=7.5, 1H), 4.3 (q, *J*=7.1, 2H), 3.7 (t, *J*=5.4, 2H), 3.7 – 3.6 (m, 8H), 3.6 (t, *J*=5.9, 2H), 3.5 (q, *J*=6.6, 2H), 3.2 (d, *J*=5.6, 2H), 2.0 (p, *J*=5.4, 2H), 1.9 (p, *J*=6.5, 2H), 1.3 (t, *J*=7.1, 3H). **^{13}C NMR** (151 MHz, $CDCl_3$) δ = 171.8, 171.2, 162.4, 139.0, 133.9, 125.9, 122.4, 116.6, 114.2, 96.8, 70.3, 70.1, 69.9, 68.9, 39.9, 37.3, 36.6, 29.3, 26.5, 13.0.

N-(3-(2-(2-(3-aminopropoxy)ethoxy)ethoxy)propyl)-7-chloro-1-ethyl-2,4-dioxo-1,2,3,4-tetrahydroquinoline-3-carboxamide hydrochloride (6b)

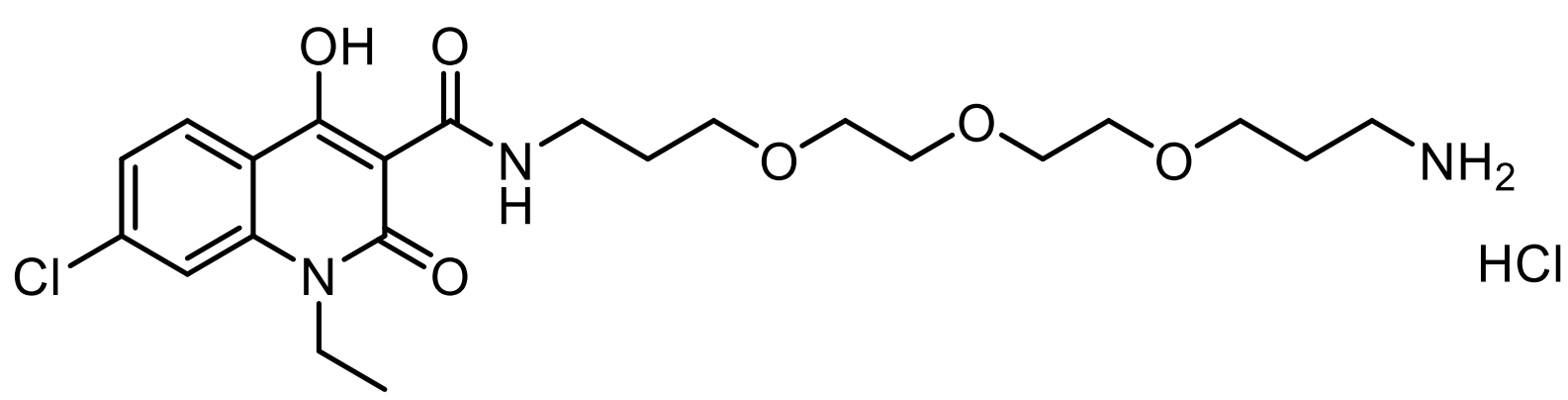

Compound was prepared according to General method G. Yield 86%

^{1}H NMR (600 MHz, $CDCl_3$) δ = 10.5 (s, 1H), 8.3 (d, *J*=8.6, 1H), 7.5 (d, *J*=1.7, 1H), 7.4 (dd, *J*=8.6, 1.8, 1H), 7.3 – 7.2 (m, 3H), 4.3 (q, *J*=7.2, 2H), 3.9 (t, *J*=5.2, 2H), 3.8 (t, *J*=5.4, 1H), 3.8 – 3.7 (m, 8H), 3.7 (t, *J*=5.8, 2H), 3.6 (q, *J*=6.9, 2H), 3.4 (h, *J*=5.8, 2H), 3.4 – 3.3 (m, 1H), 2.1 – 2.0 (m, 2H), 2.0 (p, *J*=6.4, 2H), 1.4 (t, *J*=7.2, 3H).

N-(3-(2-(2-(3-aminopropoxy)ethoxy)ethoxy)propyl)-1-benzyl-4-hydroxy-2-oxo-1,2-dihydroquinoline-3-carboxamide hydrochloride (6c)

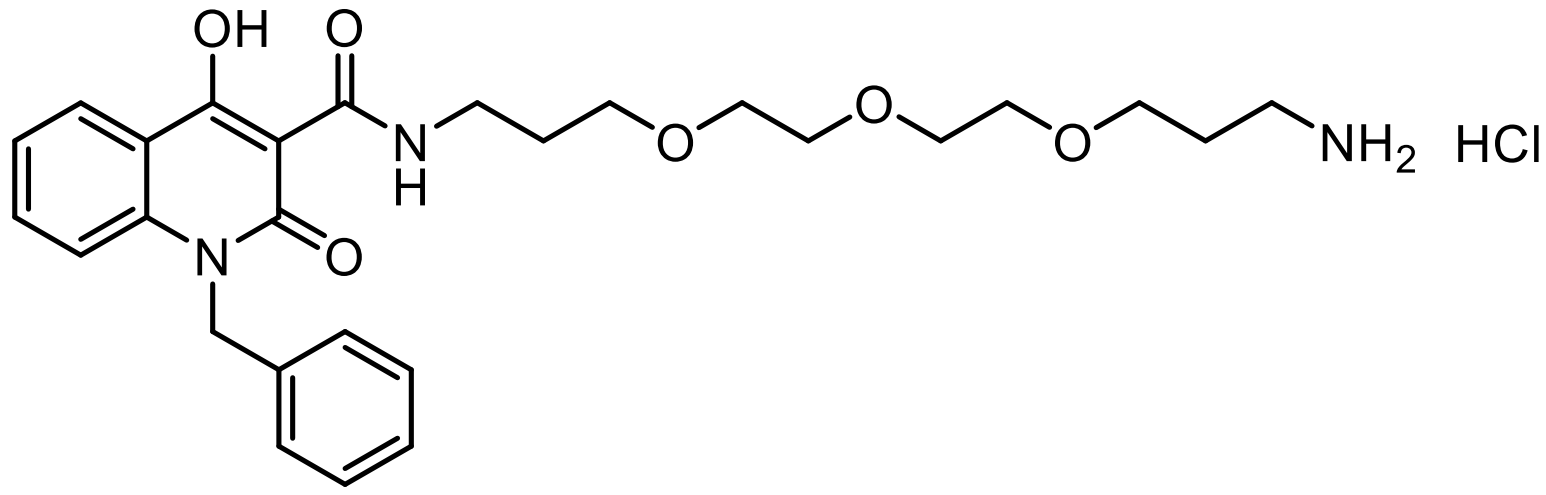

Compound was prepared according to General method G. Yield 83%

^{1}H NMR (600 MHz, $CDCl_3$) δ = 10.4 (m, 1H), 8.2 (dd, *J*=8.0, 1.6, 1H), 7.6 – 7.5 (m, 1H), 7.3 (dd, *J*=8.3, 6.8, 2H), 7.3 – 7.2 (m, 4H), 7.2 – 7.1 (m, 2H), 5.5 (s, 2H), 3.7 – 3.5 (m, 16H), 3.0 (s, 1H), 2.0 – 1.9 (m, 4H). **^{13}C NMR** (151 MHz, $CDCl_3$) δ = 172.6, 171.2, 163.0, 139.6, 136.3, 133.8, 129.1, 127.5, 126.3, 125.7, 122.6, 116.9, 115.2, 96.6, 70.5, 70.4, 70.2, 70.2, 70.1, 69.2, 45.8, 39.8, 36.9, 29.4, 8.3.

(Z)-N-(1-(4-((2,4-dioxothiazolidin-5-ylidene)methyl)phenoxy)-2-oxo-7,10,13-trioxa-3-azahexadecan-16-yl)-1-ethyl-4-hydroxy-2-oxo-1,2-dihydroquinoline-3-carboxamide (MG-1)

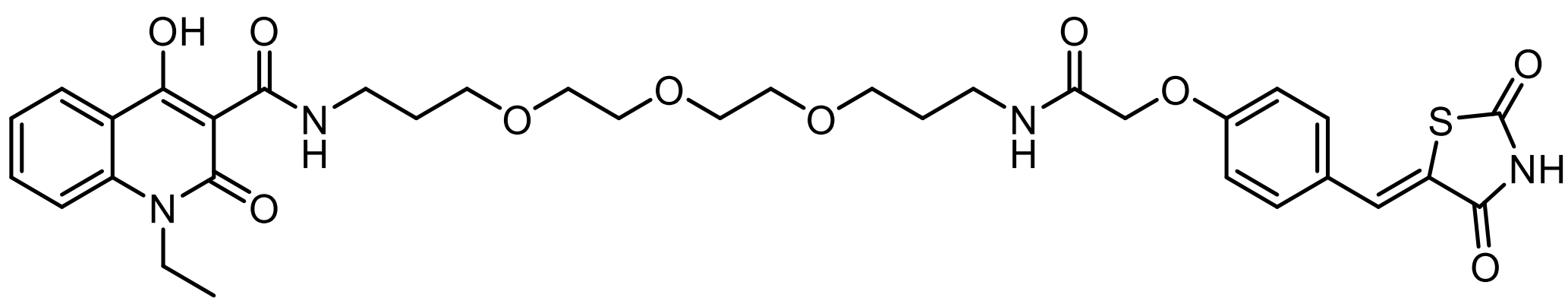

Compound was prepared according to General method H. Yield 64%

^{1}H NMR (600 MHz, $CDCl_3$) δ = 10.4 (t, *J*=5.9, 1H), 9.2 (s, 1H), 8.2 (dd, *J*=8.0, 1.6, 1H), 7.7 (s, 1H), 7.7 – 7.6 (m, 1H), 7.5 – 7.4 (m, 2H), 7.4 (d, *J*=8.6, 1H), 7.3 (t, *J*=8.1, 1H), 7.2 (t, *J*=5.7, 1H), 7.1 – 7.0 (m, 2H), 4.5 (s, 2H), 4.3 (q, *J*=7.2, 2H), 3.7 – 3.6 (m, 4H), 3.6 – 3.6 (m, 2H), 3.6 – 3.5 (m, 6H), 3.5 (q, *J*=6.8, 2H), 3.5 (q, *J*=5.8, 2H), 1.9 (p, *J*=6.5, 2H), 1.8 (p, *J*=6.0, 2H), 1.3 (t, *J*=7.1, 3H). **^{13}C NMR** (151 MHz, $CDCl_3$) δ = 171.9, 171.2, 167.8, 167.7, 167.2, 162.3, 159.1, 139.0, 133.7, 133.1, 132.4, 127.0, 125.8, 122.3, 120.9, 116.6, 115.6, 114.2, 96.8, 70.6, 70.5, 70.4, 70.3, 69.9, 69.0, 67.4, 65.9, 37.7, 37.3, 36.5, 29.4, 28.9, 13.0.

(Z)-N-(1-(4-((2,4-dioxothiazolidin-5-ylidene)methyl)-2-methoxyphenoxy)-2-oxo-7,10,13-trioxa-3-azahexadecan-16-yl)-1-ethyl-4-hydroxy-2-oxo-1,2-dihydroquinoline-3-carboxamide (MG-2)

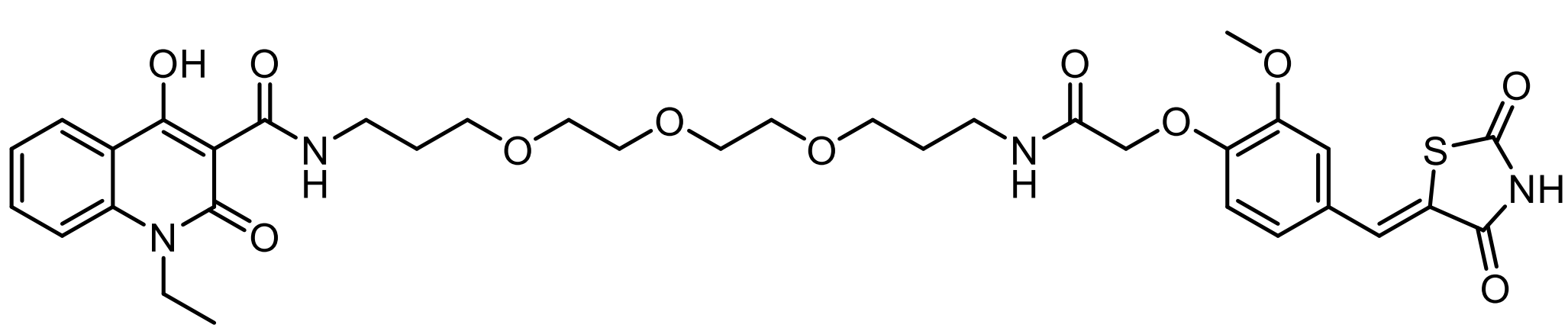

Compound was prepared according to General method H. Yield 60%

^{1}H NMR (600 MHz, DMSO) δ = 12.5 (s, 1H), 10.4 (t, *J*=5.8, 1H), 8.0 (d, *J*=8.0, 1H), 7.9 (t, *J*=5.8, 1H), 7.8 (t, *J*=8.7, 1H), 7.7 (s, 1H), 7.6 (d, *J*=8.6, 1H), 7.3 (t, *J*=7.5, 1H), 7.2 (d, *J*=2.1, 1H), 7.1 (d, *J*=8.4, 1H), 7.0 (d, *J*=8.4, 1H), 4.5 (s, 2H), 4.2 (q, *J*=7.1, 2H), 3.8 (s, 3H), 3.6 – 3.4 (m, 14H), 3.2 (q, *J*=6.5, 2H), 1.8 (p, *J*=6.5, 2H),

1.6 (p, *J*=6.7, 2H), 1.2 (t, *J*=7.0, 3H). **^{13}C NMR** (151 MHz, DMSO) δ = 171.1, 170.5, 167.9, 167.3, 167.1, 161.2, 149.2, 149.2, 138.6, 134.2, 131.9, 126.6, 124.7, 123.3, 122.2, 121.0, 115.4, 114.9, 113.9, 113.6, 95.8, 69.8, 69.7, 69.5, 68.2, 68.2, 67.8, 55.6, 36.6, 36.2, 35.9, 29.2, 28.8, 12.7.

(Z)-N-(1-(2,6-dibromo-4-((2,4-dioxothiazolidin-5-ylidene)methyl)phenoxy)-2-oxo-7,10,13-trioxa-3-azahexadecan-16-yl)-1-ethyl-4-hydroxy-2-oxo-1,2-dihydroquinoline-3-carboxamide (MG-3)

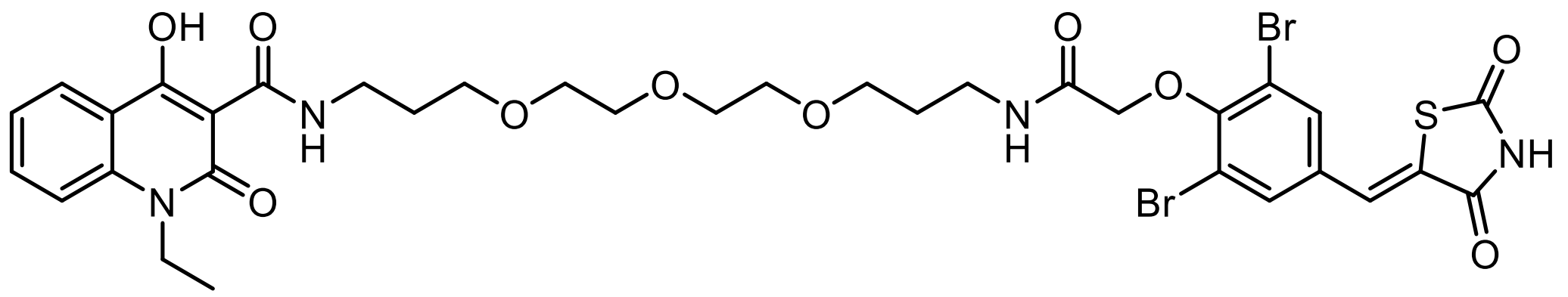

Compound was prepared according to General method H. Yield 63%

^{1}H NMR (600 MHz, DMSO) δ = 10.4 (t, *J*=5.8, 1H), 9.9 (s, 1H), 8.1 (s, 2H), 8.1 (t, *J*=5.8, 1H), 8.1 – 8.0 (m, 1H), 7.8 – 7.7 (m, 1H), 7.6 – 7.6 (m, 1H), 7.3 (q, *J*=7.8, 1H), 5.7 (s, 1H), 4.4 (s, 2H), 4.3 (q, *J*=7.1, 2H), 3.6 – 3.5 (m, 10H), 3.5 – 3.4 (m, 4H), 3.2 (q, *J*=6.6, 2H), 1.8 (p, *J*=6.4, 2H), 1.7 (p, *J*=6.6, 2H), 1.2 (q, *J*=6.9, 3H). **^{13}C NMR** (151 MHz, DMSO) δ = 189.9, 171.1, 170.5, 165.9, 161.2, 156.0, 138.6, 134.7, 134.2, 133.7, 130.5, 124.7, 122.3, 118.4, 115.4, 115.0, 95.8, 94.8, 70.9, 69.8, 69.6, 69.6, 68.4, 68.3, 68.2, 54.9, 48.6, 36.6, 36.3, 36.1, 29.1, 28.8, 12.7.

(Z)-7-chloro-N-(1-(4-((2,4-dioxothiazolidin-5-ylidene)methyl)phenoxy)-2-oxo-7,10,13-trioxa-3-azahexadecan-16-yl)-1-ethyl-4-hydroxy-2-oxo-1,2-dihydroquinoline-3-carboxamide (MG-4)

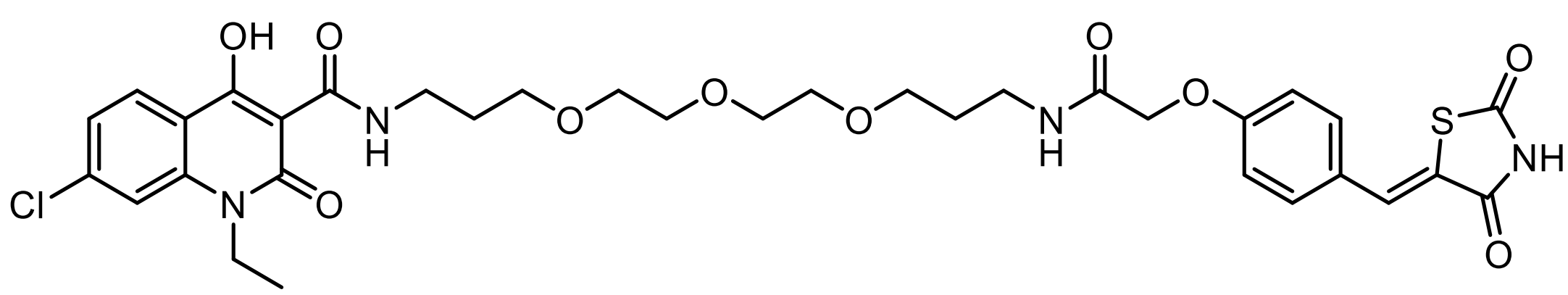

Compound was prepared according to General method H. Yield 61%

^{1}H NMR (600 MHz, $CDCl_3$) δ = 10.2 (t, *J*=5.8, 1H), 10.1 (s, 1H), 8.0 (d, *J*=8.5, 1H), 7.7 (s, 1H), 7.4 (d, *J*=8.7, 2H), 7.3 (s, 1H), 7.2 (dd, *J*=8.5, 1.7, 1H), 7.1 (t, *J*=5.7, 1H), 7.0 (d, *J*=8.6, 2H), 4.5 (s, 2H), 4.2 (q, *J*=7.1, 2H), 3.6 – 3.6 (m, 4H), 3.6 – 3.5 (m, 8H), 3.5 – 3.4 (m, 4H), 1.9 (h, *J*=6.7, 2H), 1.8 (p, *J*=6.0, 2H), 1.3 (t, *J*=7.2, 3H). **^{13}C NMR** (151 MHz, $CDCl_3$) δ = 171.6, 171.0, 167.6, 167.2, 162.8, 159.1, 140.1, 139.7, 133.0, 132.3, 127.2, 127.1, 122.8, 120.9, 115.6, 115.1, 114.1, 96.7, 70.6, 70.5, 70.4, 70.3, 70.0, 69.0, 67.5, 38.7, 37.7, 37.5, 36.6, 29.4, 29.0, 12.9.

(Z)-7-chloro-N-(1-(4-((2,4-dioxothiazolidin-5-ylidene)methyl)-2-methoxyphenoxy)-2-oxo-7,10,13-trioxa-3-azahexadecan-16-yl)-1-ethyl-4-hydroxy-2-oxo-1,2-dihydroquinoline-3-carboxamide (MG-5)

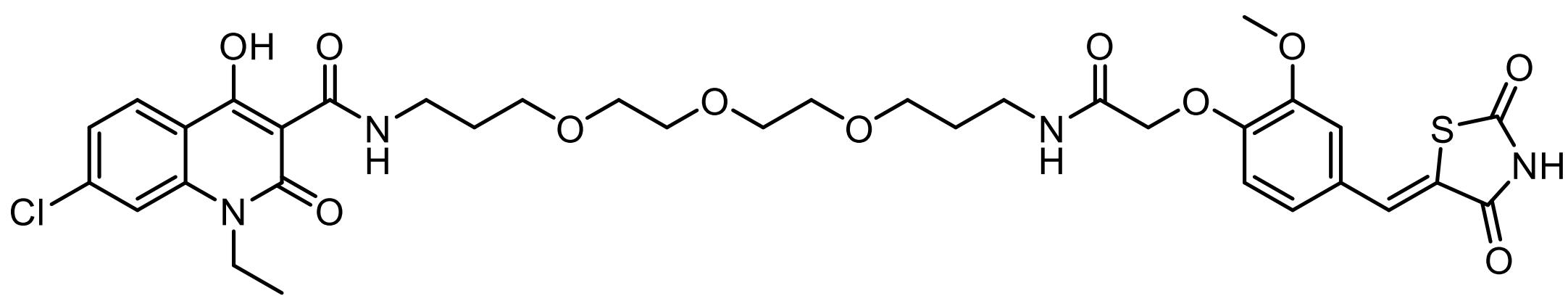

Compound was prepared according to General method H. Yield 65%

^{1}H NMR (600 MHz, $CDCl_3$) δ = 10.2 (t, *J*=5.8, 1H), 8.0 (d, *J*=8.5, 1H), 7.9 (s, 1H), 7.3 (s, 1H), 7.2 (d, *J*=8.6, 1H), 7.1 (t, *J*=5.8, 1H), 7.0 (d, *J*=8.5, 1H), 7.0 (s, 1H), 6.9 (d, *J*=8.4, 1H), 6.2 (s, 1H), 4.5 (s, 2H), 4.2 (q, *J*=7.1, 2H), 3.9 (s, 3H), 3.7 – 3.4 (m, 16H), 1.8 (p, *J*=6.4, 2H), 1.8 (p, *J*=6.3, 2H), 1.3 (t, *J*=7.1, 3H). **^{13}C NMR** (151 MHz, $CDCl_3$) δ = 171.5, 171.0, 167.6, 162.2, 160.9, 151.1, 150.3, 150.0, 140.1, 139.8, 139.4, 127.1, 126.7, 125.1, 122.8, 115.4, 115.1, 114.4, 114.1, 113.7, 96.7, 70.6, 70.5, 70.4, 70.3, 69.4, 69.0, 68.4, 37.4, 37.3, 36.7, 29.4, 29.2, 12.9.

(E)-7-chloro-N-(1-(2,6-dibromo-4-((2,4-dioxothiazolidin-5-ylidene)methyl)phenoxy)-2-oxo-7,10,13-trioxa-3-azahexadecan-16-yl)-1-ethyl-4-hydroxy-2-oxo-1,2-dihydroquinoline-3-carboxamide (MG-6)

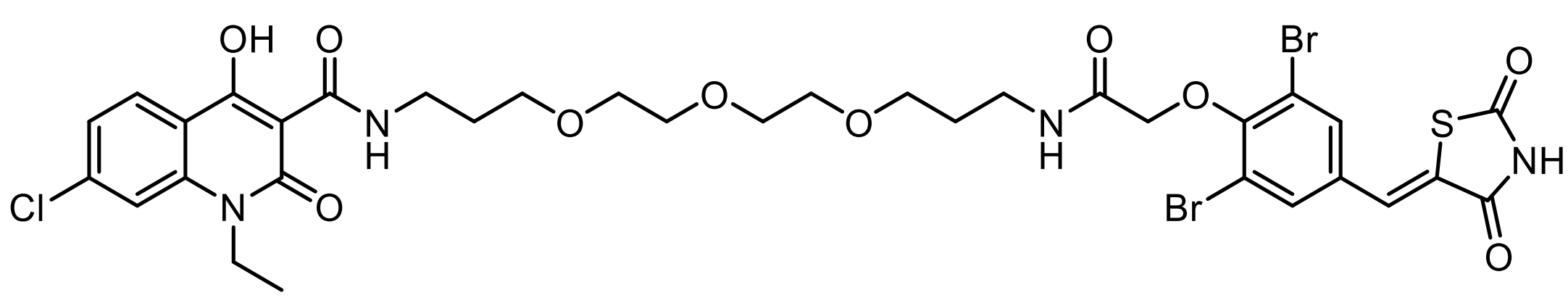

Compound was prepared according to General method H. Yield 60%

^{1}H NMR (600 MHz, $CDCl_3$) δ = 10.2 (t, *J*=5.8, 1H), 9.8 (s, 1H), 8.1 (d, *J*=8.6, 1H), 8.0 – 8.0 (m, 2H), 7.4 (t, *J*=5.8, 1H), 7.3 (s, 1H), 7.2 (d, *J*=8.6, 1H), 4.7 (s, 1H), 4.6 (s, 2H), 4.2 (q, *J*=7.1, 2H), 3.7 – 3.5 (m, 17H), 1.9 (p, *J*=6.3, 4H), 1.3 (t, *J*=7.1, 3H). **^{13}C NMR** (151 MHz, $CDCl_3$) δ = 188.4, 188.2, 171.5, 171.0, 169.1, 167.2, 156.8, 156.4, 140.1, 139.8, 134.9, 134.8, 134.0, 134.0, 127.2, 122.8, 119.1, 119.0, 115.1, 114.1, 96.7, 71.0, 70.6, 70.5, 70.4, 69.8, 69.0, 68.7, 37.7, 37.5, 36.7, 29.4, 29.1, 12.9.

(Z)-1-benzyl-N-(1-(4-((2,4-dioxothiazolidin-5-ylidene)methyl)phenoxy)-2-oxo-7,10,13-trioxa-3-azahexadecan-16-yl)-4-hydroxy-2-oxo-1,2-dihydroquinoline-3-carboxamide (MG-7)

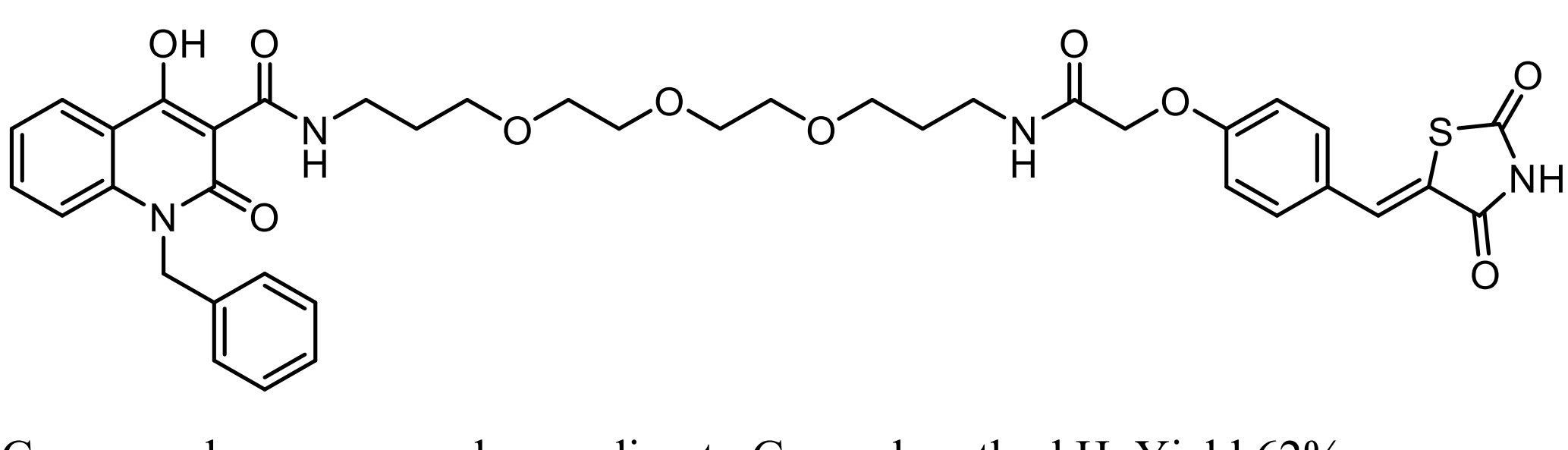

Compound was prepared according to General method H. Yield 62%

^{1}H NMR (600 MHz, DMSO) δ = 10.3 (t, *J*=5.7, 1H), 9.0 (t, *J*=5.9, 1H), 8.2 (d, *J*=8.3, 1H), 8.2 – 8.1 (m, 3H), 7.9 – 7.8 (m, 2H), 7.8 – 7.8 (m, 1H), 7.7 – 7.6 (m, 4H), 7.4 (d, *J*=8.6, 1H), 7.3 – 7.3 (m, 3H), 7.2 – 7.2 (m, 3H), 5.5 (s, 2H), 3.6 – 3.4 (m, 16H), 1.8 (m, 4H). **^{13}C NMR** (151 MHz, DMSO) δ = 171.7, 170.5, 168.2, 162.8, 161.8, 154.0, 150.4, 139.1, 136.6, 136.3, 134.8, 134.2, 130.3, 130.2, 129.5, 129.1, 128.6, 128.6, 127.1, 126.9, 126.3, 124.7, 122.6, 122.2, 115.7, 115.6, 95.7, 69.7, 69.7, 69.6, 69.6, 68.6, 68.3, 44.6, 37.1, 36.4, 29.2, 28.8.

N-(1-(1-ethyl-4-hydroxy-2-oxo-1,2-dihydroquinolin-3-yl)-1-oxo-6,9,12-trioxa-2-azapentadecan-15-yl)-4-phenylquinazoline-2-carboxamide (MG-8)

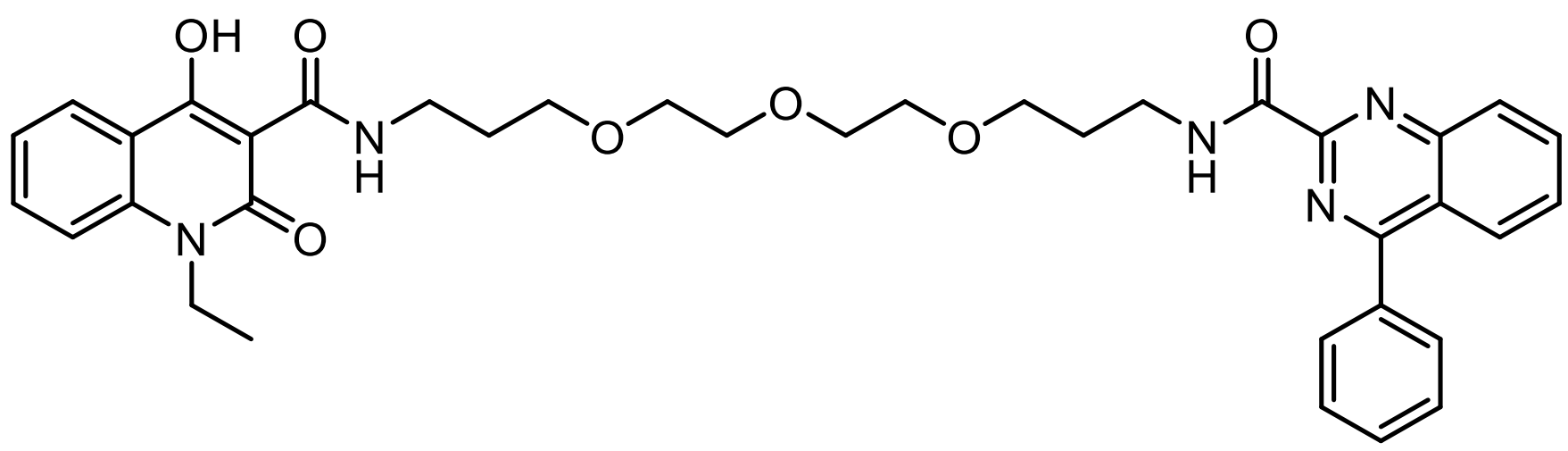

Compound was prepared according to General method H. Yield 60%

^{1}H NMR (600 MHz, $CDCl_3$) δ = 10.3 (t, *J*=5.8, 1H), 8.6 (t, *J*=5.9, 1H), 8.3 (d, *J*=8.5, 1H), 8.1 (d, *J*=8.1, 1H), 8.1 (d, *J*=8.4, 1H), 7.9 (t, *J*=8.3, 1H), 7.8 – 7.7 (m, 2H), 7.6 – 7.5 (m, 2H), 7.5 – 7.5 (m, 3H), 7.3 (s, 1H), 7.2 – 7.1 (m, 1H), 4.2 (q, *J*=7.2, 2H), 3.6 (q, *J*=6.4, 2H), 3.6 (t, *J*=6.0, 2H), 3.5 – 3.5 (m, 6H), 3.5 – 3.5 (m, 4H), 3.4 (q, *J*=6.5, 2H), 1.9 (p, *J*=6.3, 2H), 1.9 – 1.8 (m, 2H), 1.2 (t, *J*=7.2, 3H). **^{13}C NMR** (151 MHz, $CDCl_3$) δ = 171.9, 171.2, 168.8, 163.1, 162.3, 153.5, 151.5, 139.0, 136.8, 134.3, 133.7, 130.4, 130.3, 130.2, 129.1, 128.7, 127.2, 125.8, 123.1, 122.2, 116.7, 114.1, 96.8, 70.7, 70.6, 70.5, 70.4, 69.8, 69.0, 38.1, 37.2, 36.6, 29.5, 29.4, 29.4, 13.0.

N-(1-(7-chloro-1-ethyl-2,4-dioxo-1,2,3,4-tetrahydroquinolin-3-yl)-1-oxo-6,9,12-trioxa-2-azapentadecan-15-yl)-4-phenylquinazoline-2-carboxamide (MG-9)

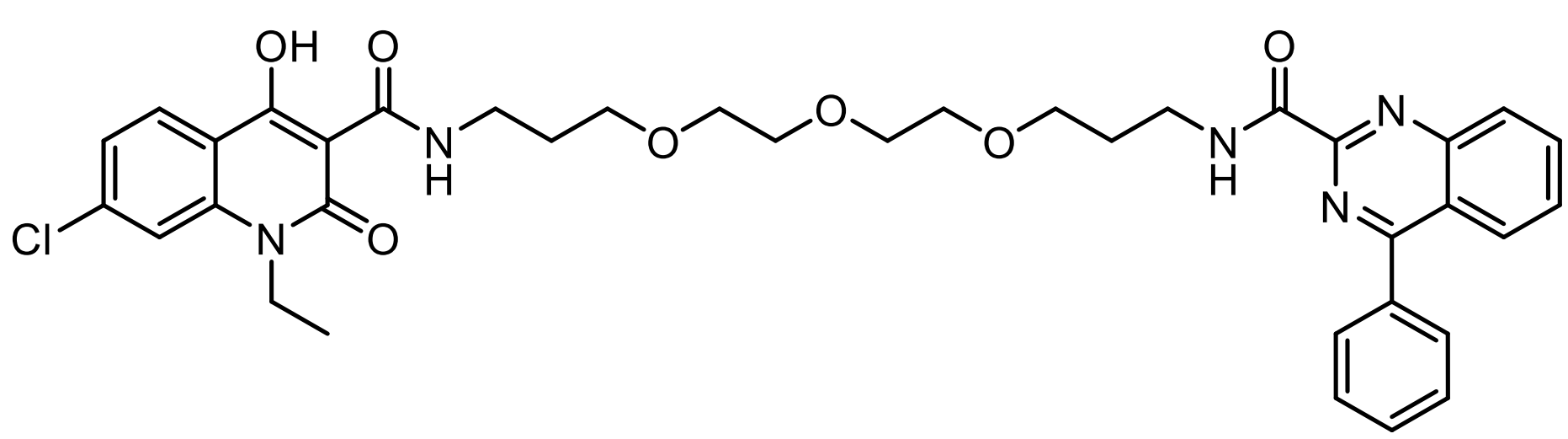

Compound was prepared according to General method H. Yield 56%

^{1}H NMR (600 MHz, $CDCl_3$) δ = 10.2 (t, *J*=5.8, 1H), 8.7 (t, *J*=5.9, 1H), 8.3 (d, *J*=8.4, 1H), 8.1 (dd, *J*=8.4, 1.3, 1H), 8.0 (d, *J*=8.5, 1H), 7.9 (t, *J*=8.3, 1H), 7.8 – 7.7 (m, 2H), 7.6 (dd, *J*=8.4, 6.9, 1H), 7.6 – 7.5 (m, 3H), 7.3 (s, 1H), 7.2 (dd, *J*=8.6, 1.8, 1H), 4.2 (q, *J*=7.2, 2H), 3.6 (q, *J*=6.4, 2H), 3.6 (t, *J*=5.9, 2H), 3.6 – 3.6 (m, 6H), 3.6 – 3.5 (m, 4H), 3.5 (q, *J*=6.5, 2H), 1.9 (p, *J*=6.2, 2H), 1.9 (p, *J*=6.4, 2H), 1.3 (t, *J*=7.2, 3H). **^{13}C NMR** (151 MHz, $CDCl_3$) δ = 171.4, 170.9, 168.7, 163.1, 153.3, 151.3, 140.0, 139.7, 136.7, 134.3, 130.4, 130.1, 130.0, 129.1, 128.6, 127.1, 127.0, 123.0, 122.6, 115.0, 114.0, 96.6, 70.5, 70.5, 70.4, 70.3, 69.7, 68.9, 38.6, 38.0, 37.3, 36.6, 29.3, 12.8.

N-(1-(1-benzyl-4-hydroxy-2-oxo-1,2-dihydroquinolin-3-yl)-1-oxo-6,9,12-trioxa-2-azapentadecan-15-yl)-4-phenylquinazoline-2-carboxamide (MG-10)

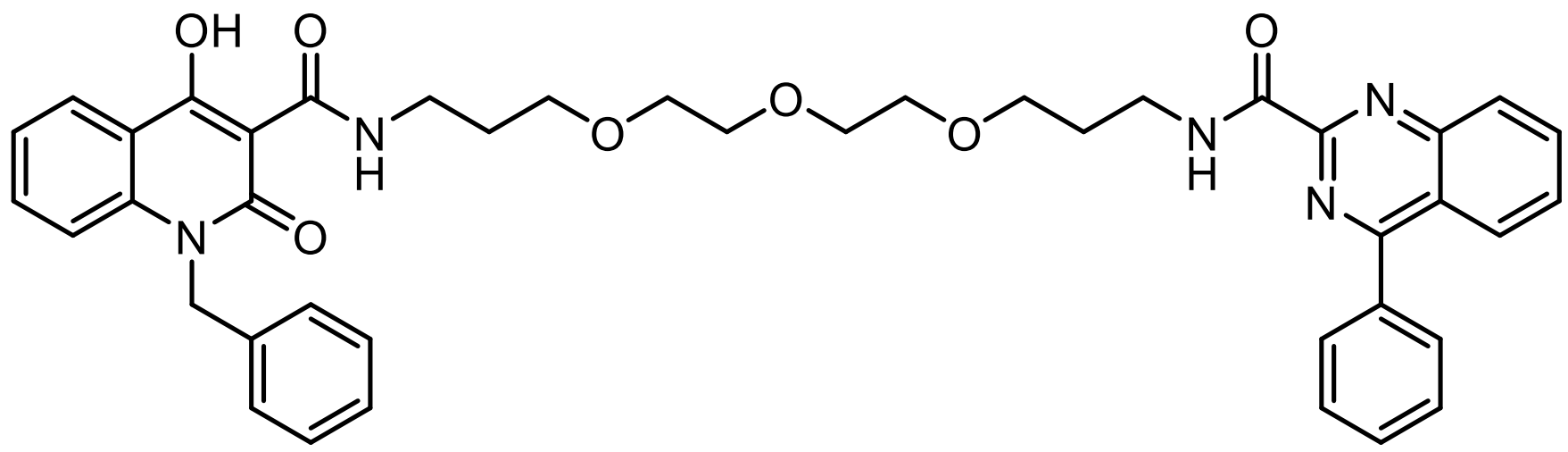

Compound was prepared according to General method H. Yield 57%

^{1}H NMR (600 MHz, DMSO) δ = 10.3 (t, *J*=5.7, 1H), 9.0 (t, *J*=5.9, 1H), 8.2 (d, *J*=8.3, 1H), 8.2 – 8.1 (m, 3H), 7.9 – 7.8 (m, 2H), 7.8 – 7.8 (m, 1H), 7.7 – 7.6 (m, 4H), 7.4 (d, *J*=8.6, 1H), 7.3 – 7.3 (m, 3H), 7.2 – 7.2 (m, 3H), 5.5 (s, 2H), 3.6 – 3.4 (m, 16H), 1.8 (m, 4H). **^{13}C NMR** (151 MHz, DMSO) δ = 171.7, 170.5, 168.2, 162.8, 161.8, 154.0, 150.4, 139.1, 136.6, 136.3, 134.8, 134.2, 130.3, 130.2, 129.5, 129.1, 128.6, 128.6, 127.1, 126.9, 126.3, 124.7, 122.6, 122.2, 115.7, 115.6, 95.7, 69.7, 69.7, 69.6, 69.6, 68.6, 68.3, 44.6, 37.1, 36.4, 29.2, 28.8.

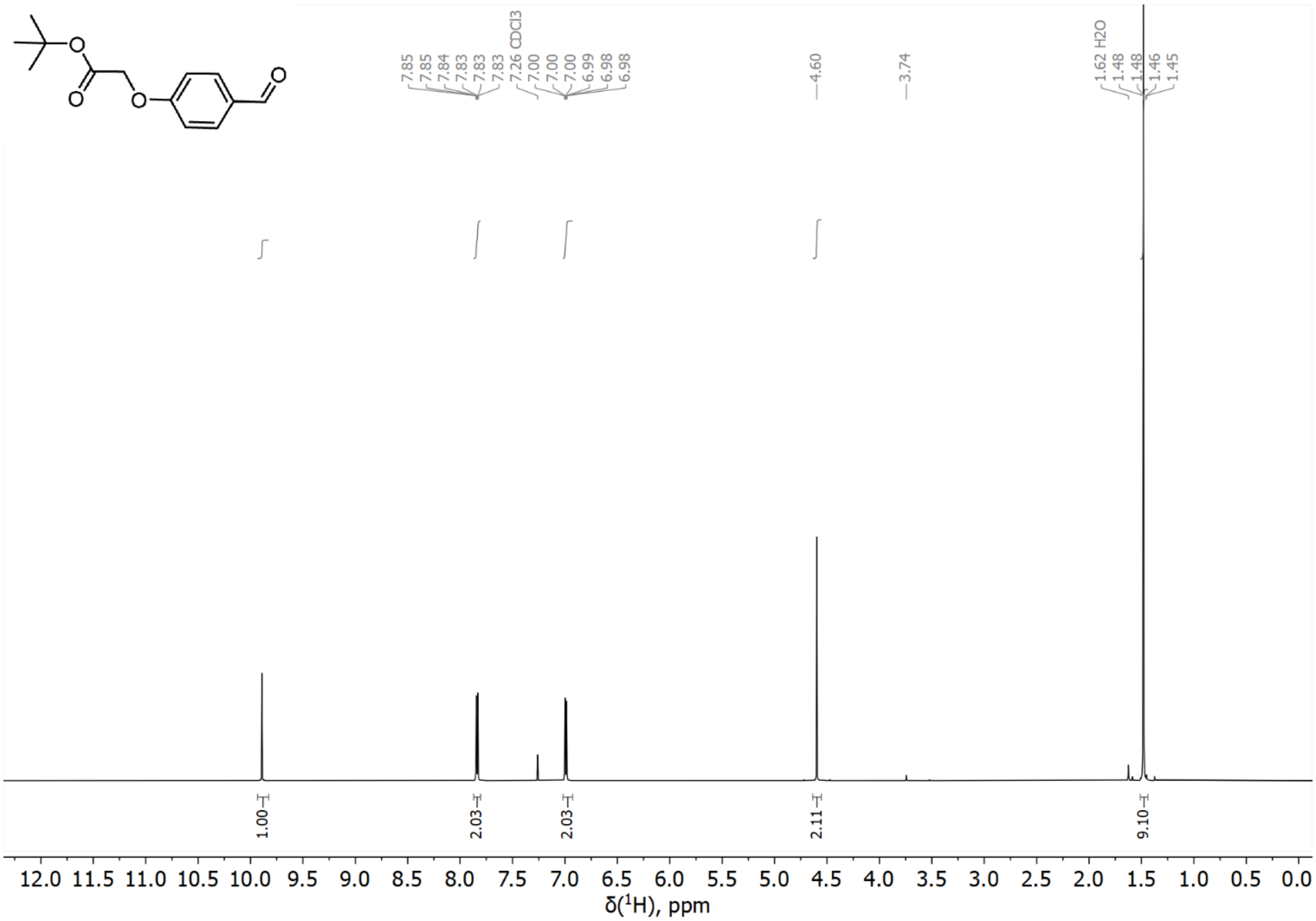

Supplementary Figure S11. ^{1}H and ^{13}C NMR spectrum of **tert-butyl 2-(4-formylphenoxy)acetate**

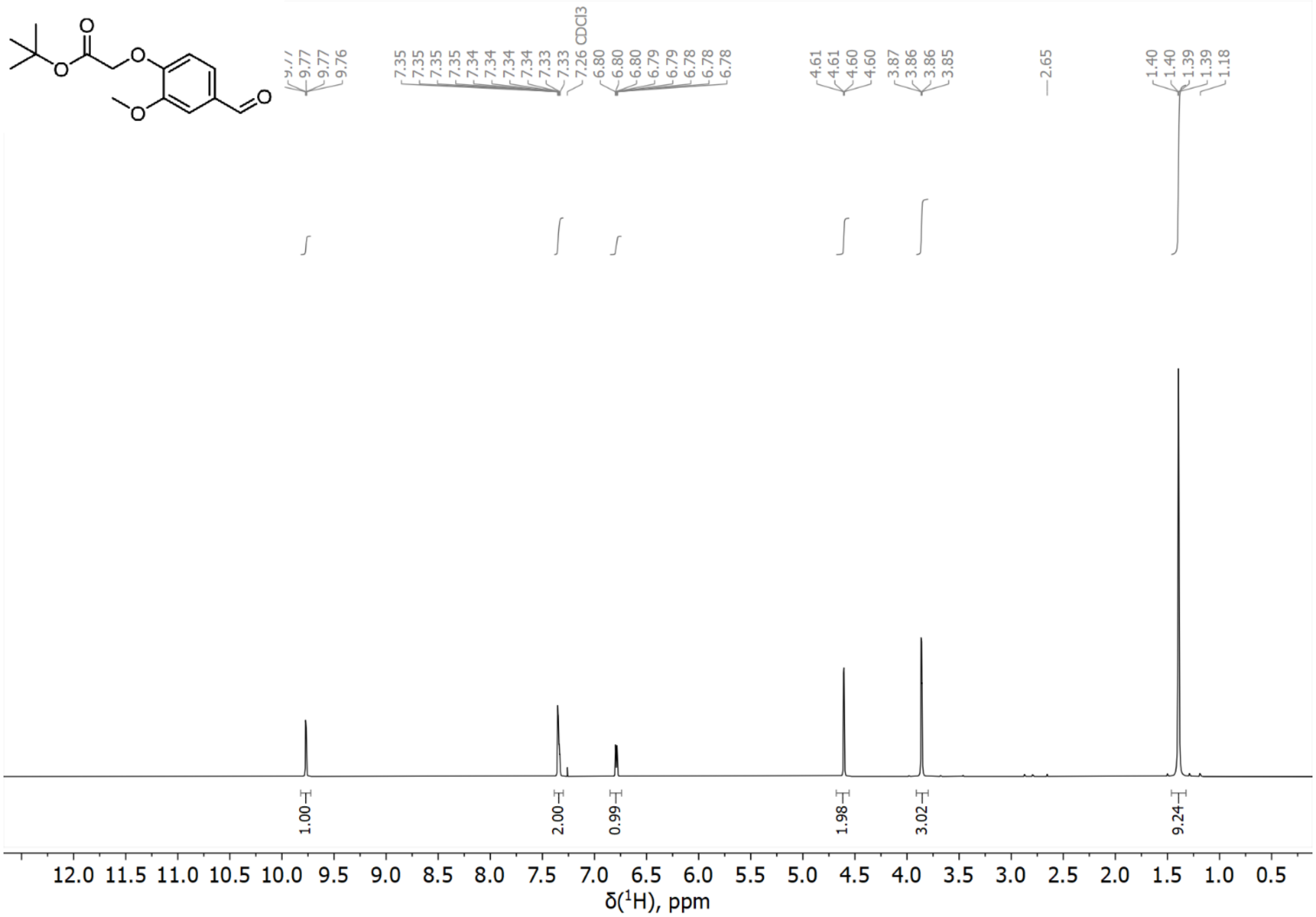

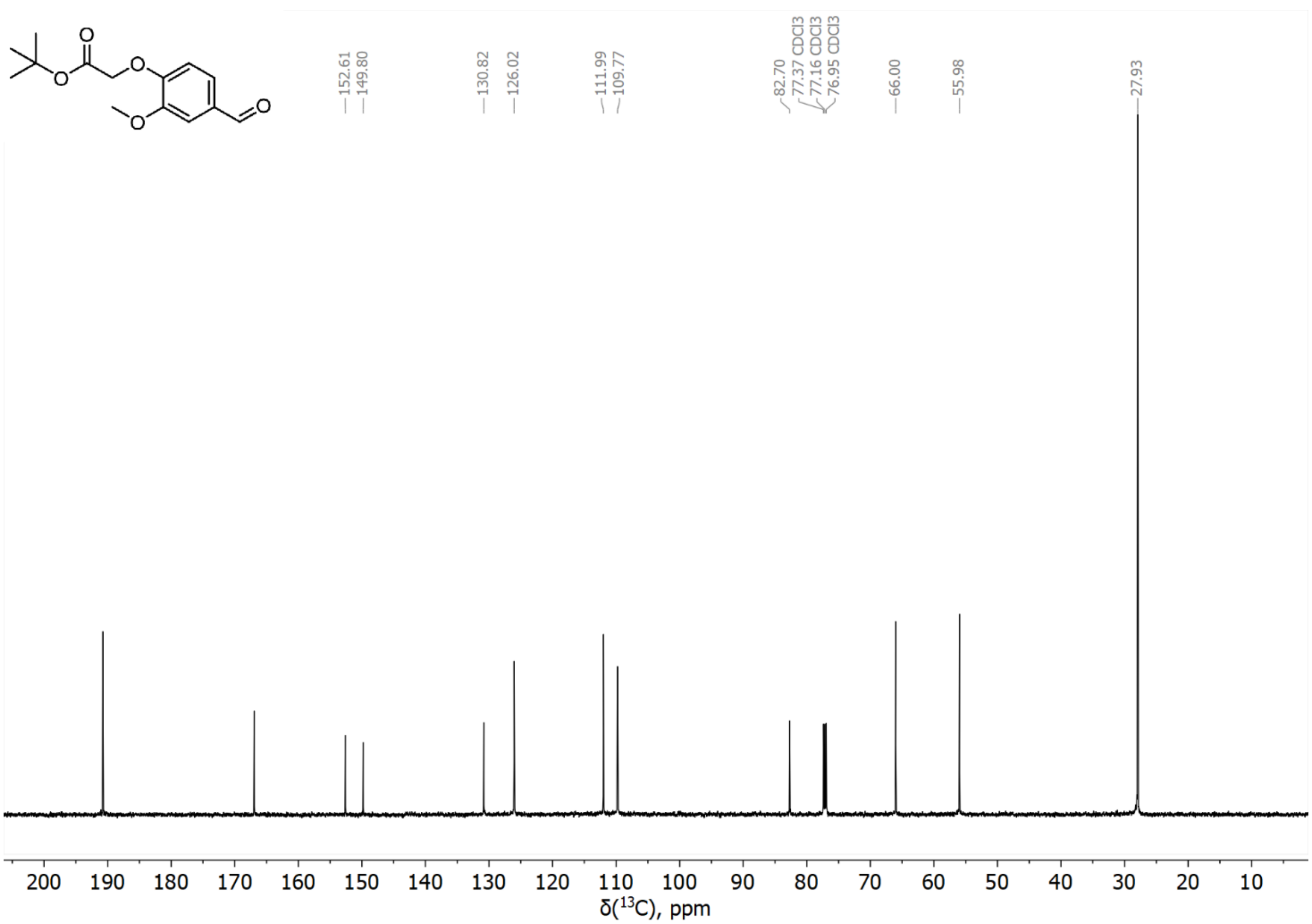

Supplementary Figure S12. ^{1}H and ^{13}C NMR spectrum of **tert-butyl 2-(4-formyl-2-methoxyphenoxy)acetate**

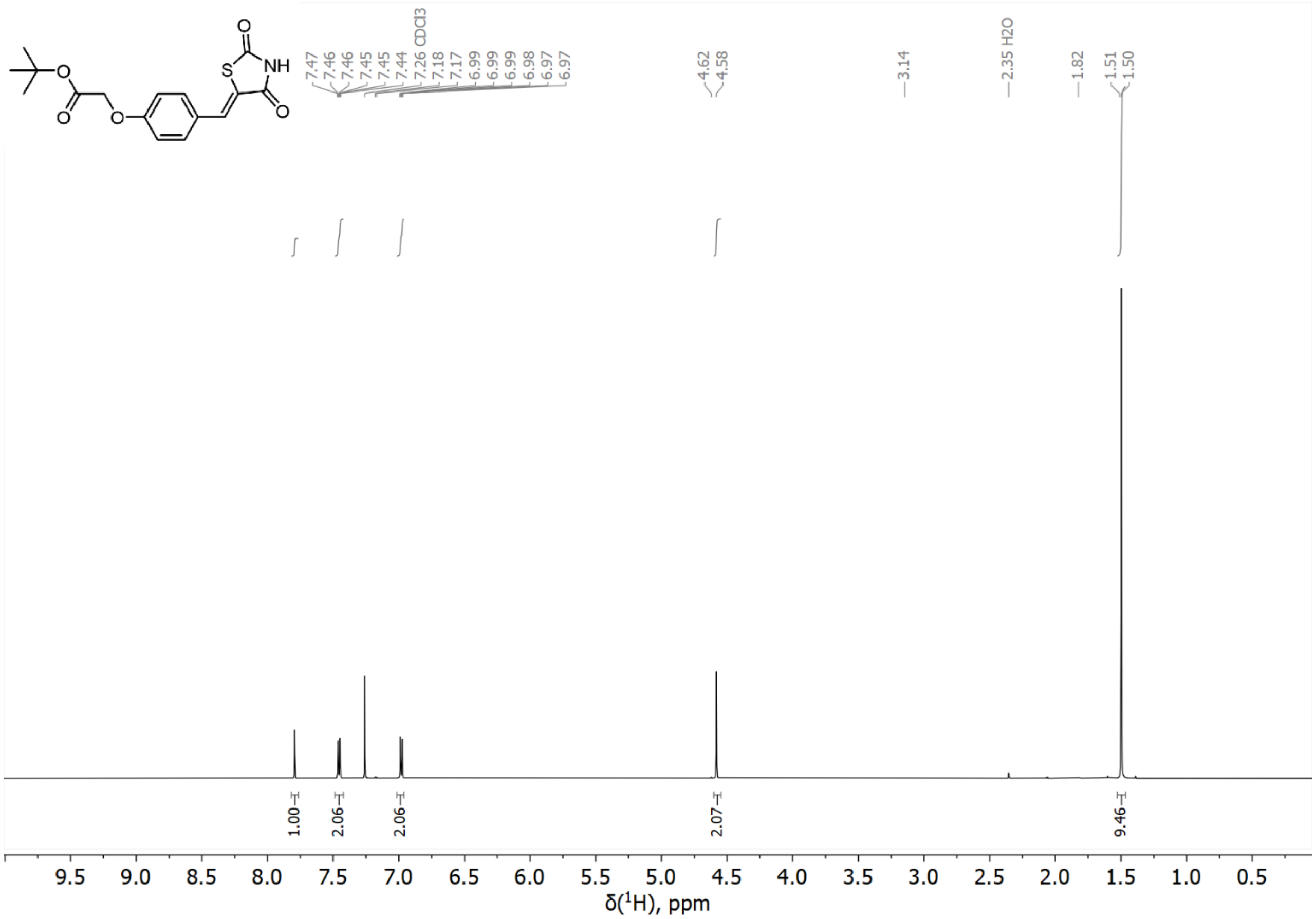

Supplementary Figure S13. ^{1}H and ^{13}C NMR spectrum of **tert-butyl (Z)-2-(4-((2,4-dioxothiazolidin-5-ylidene)methyl)phenoxy)acetate**

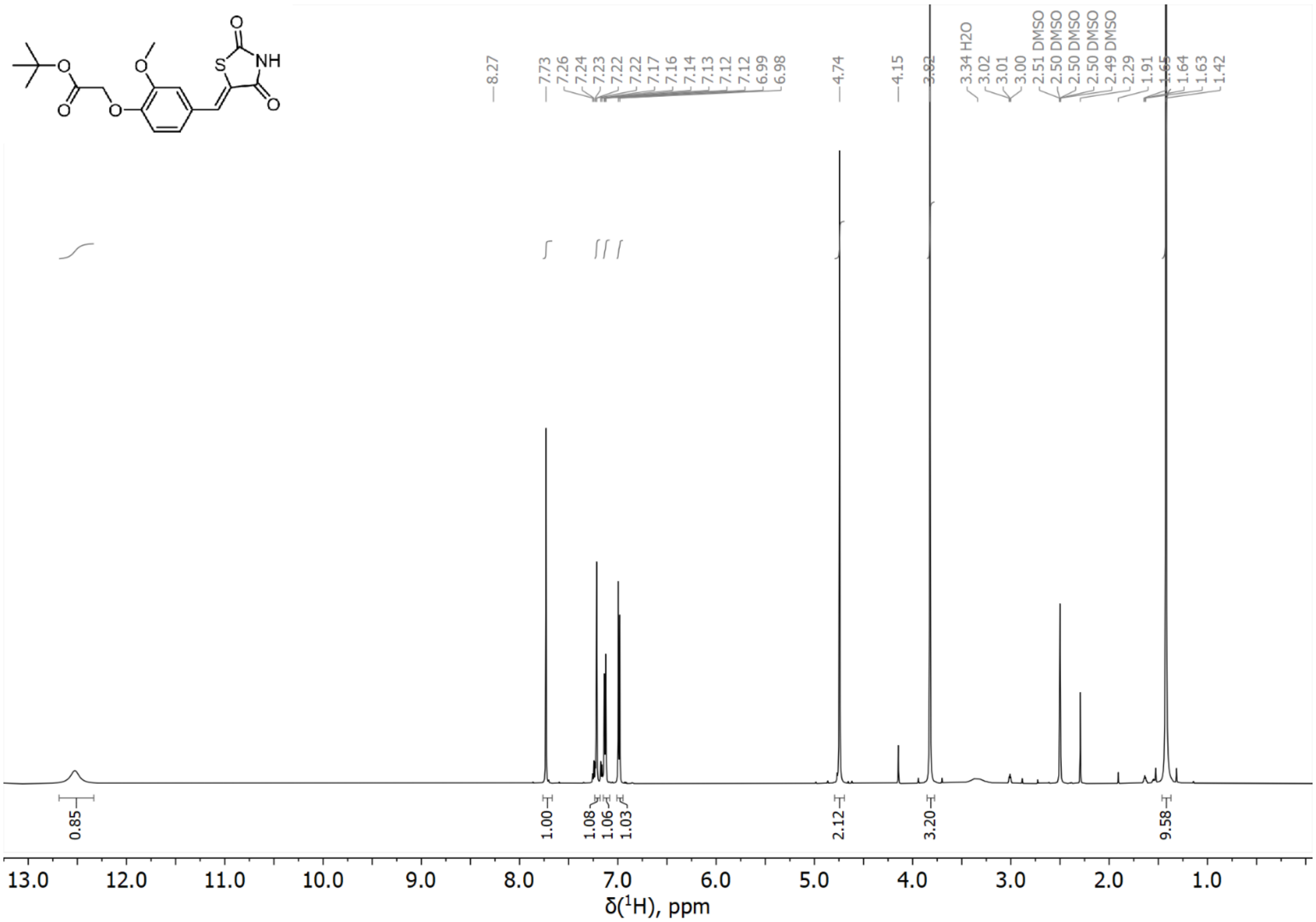

Supplementary Figure S14. ^{1}H and ^{13}C NMR spectrum of **tert-butyl (Z)-2-(4-((2,4-dioxothiazolidin-5-ylidene)methyl)-2-methoxyphenoxy)acetate**

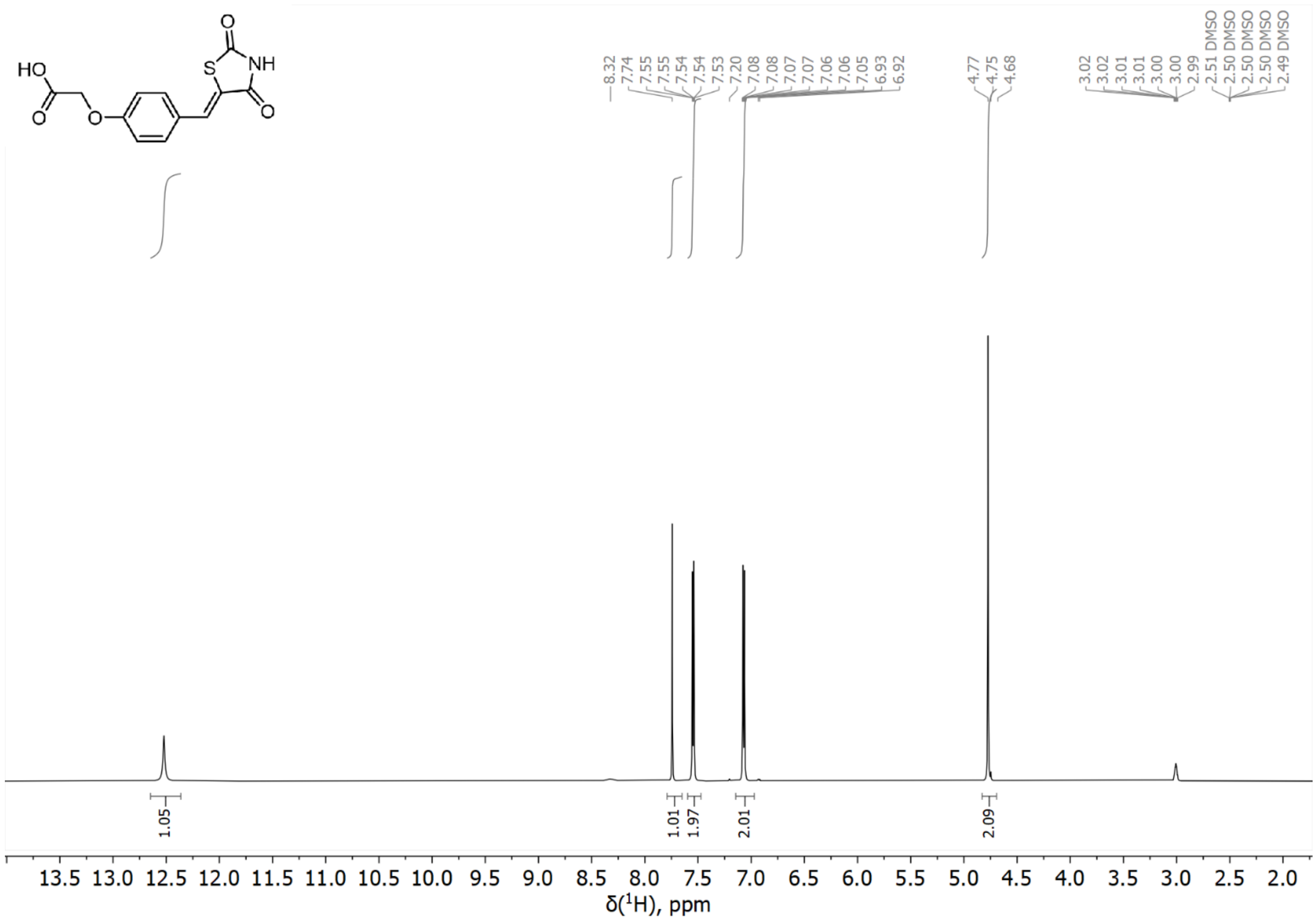

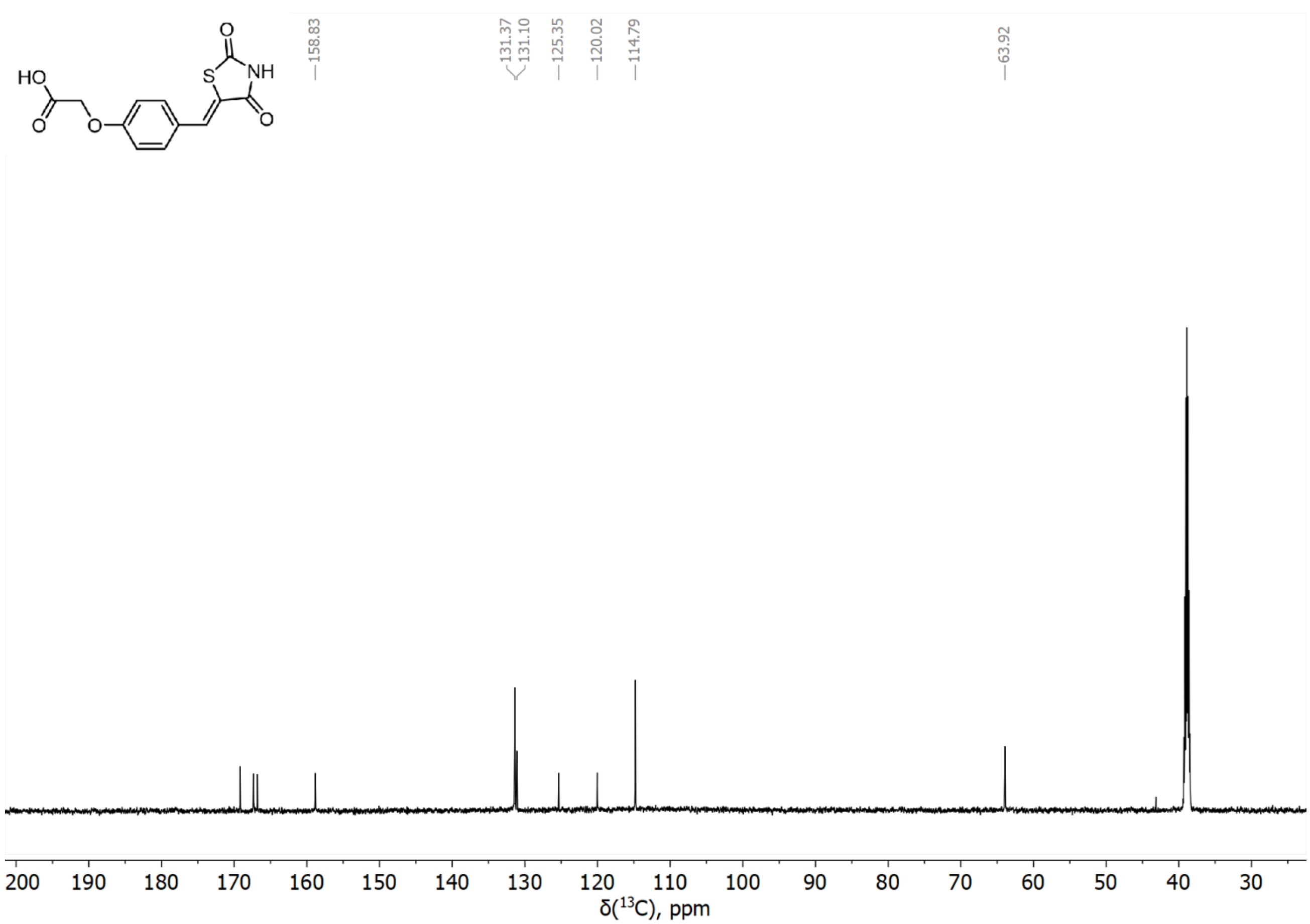

Supplementary Figure S15. ^{1}H and ^{13}C NMR spectrum of **1a**

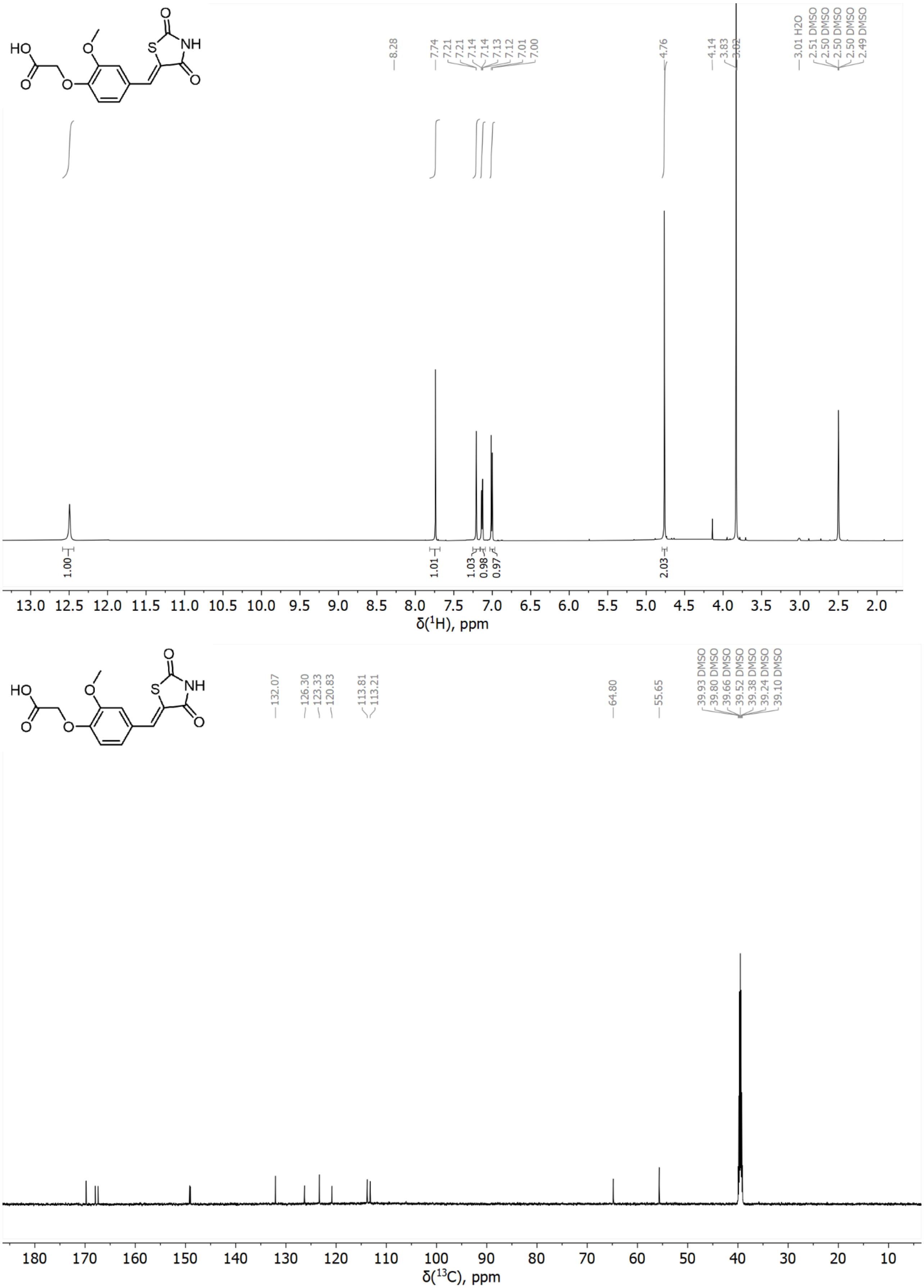

Supplementary Figure S16. ^{1}H and ^{13}C NMR spectrum of **1b**

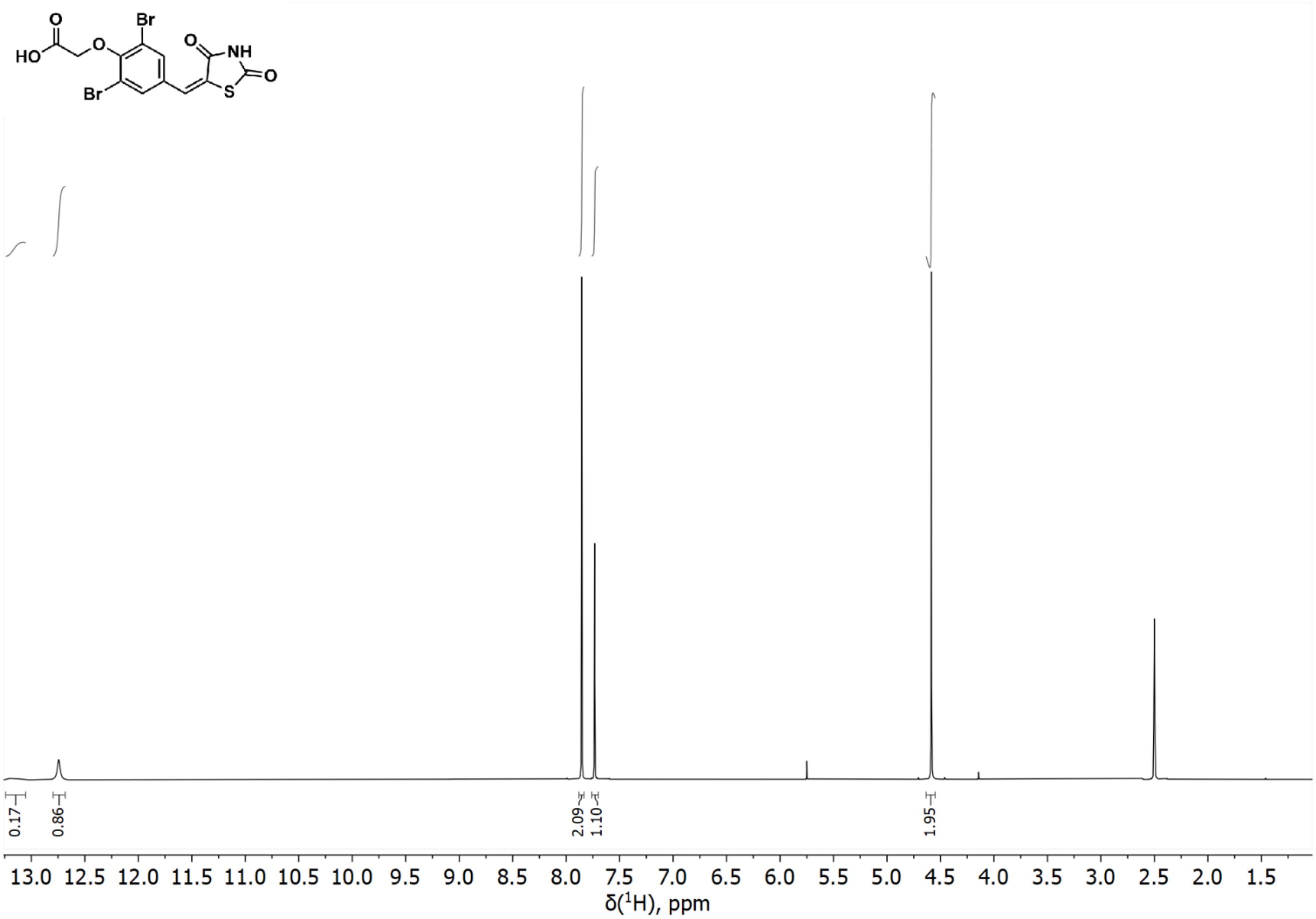

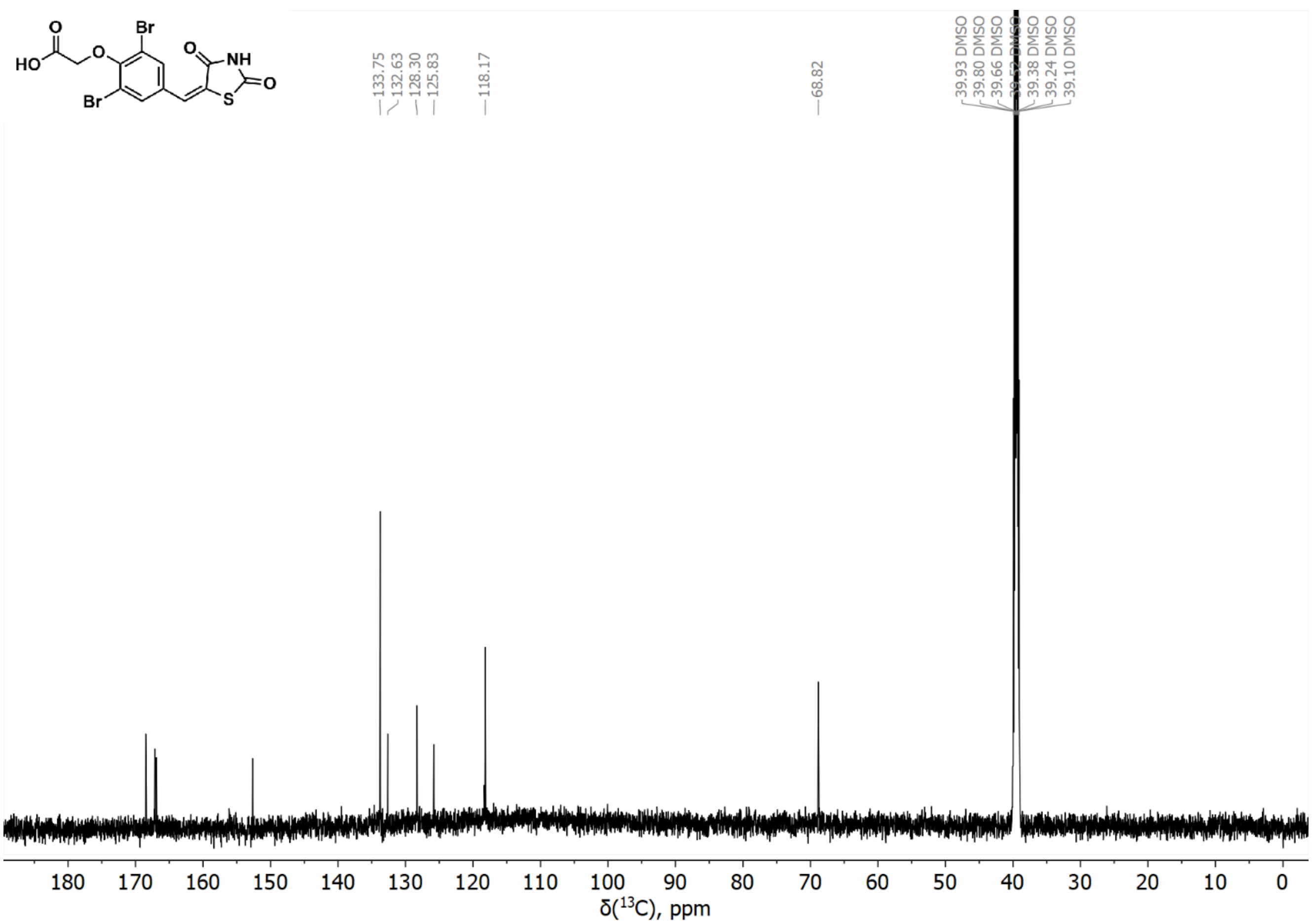

Supplementary Figure S17. ^{1}H and ^{13}C NMR spectrum of **1c**

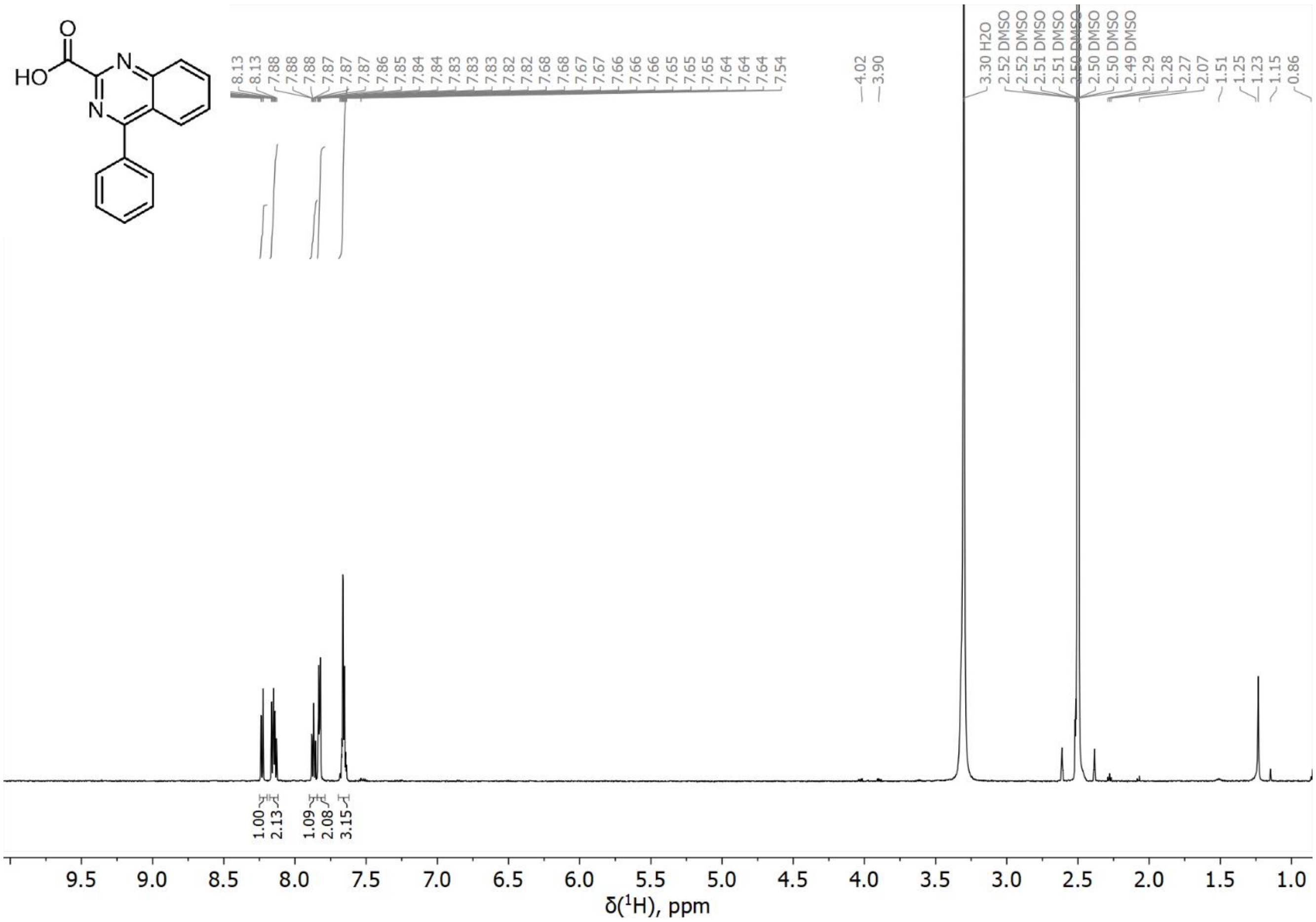

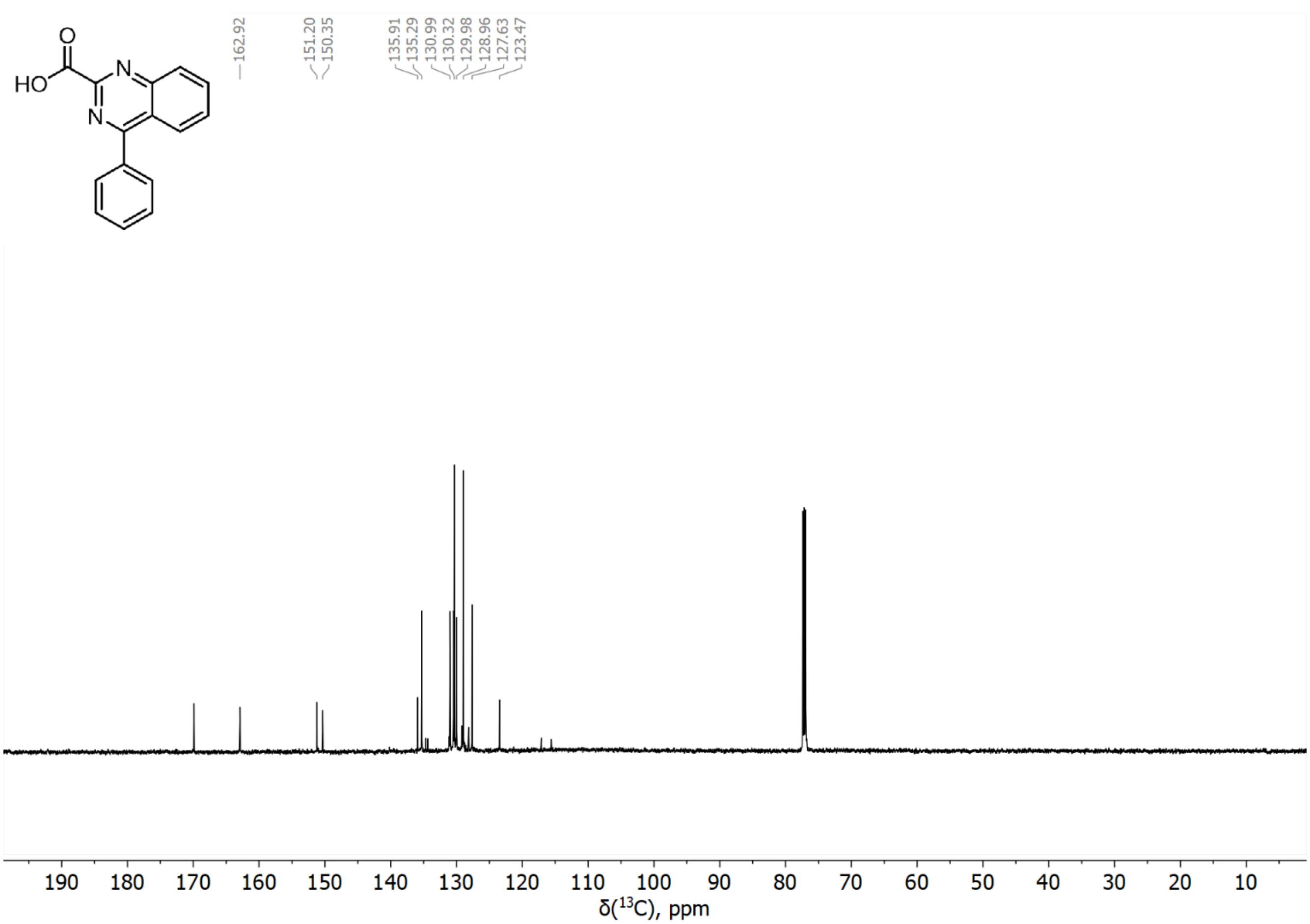

Supplementary Figure S18. ^{1}H and ^{13}C NMR spectrum of **2**

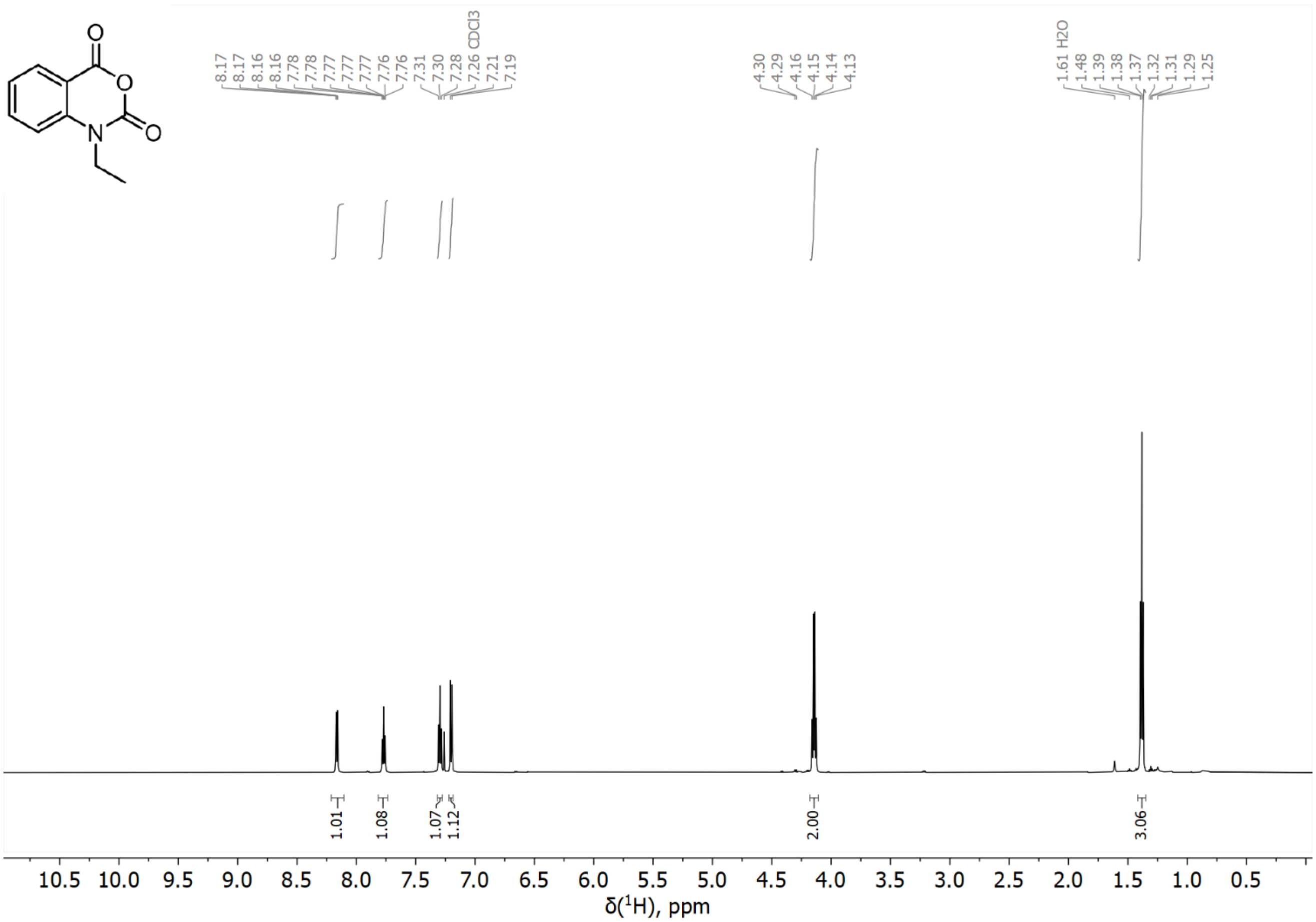

Supplementary Figure S19. ^{1}H and ^{13}C NMR spectrum of **4a**

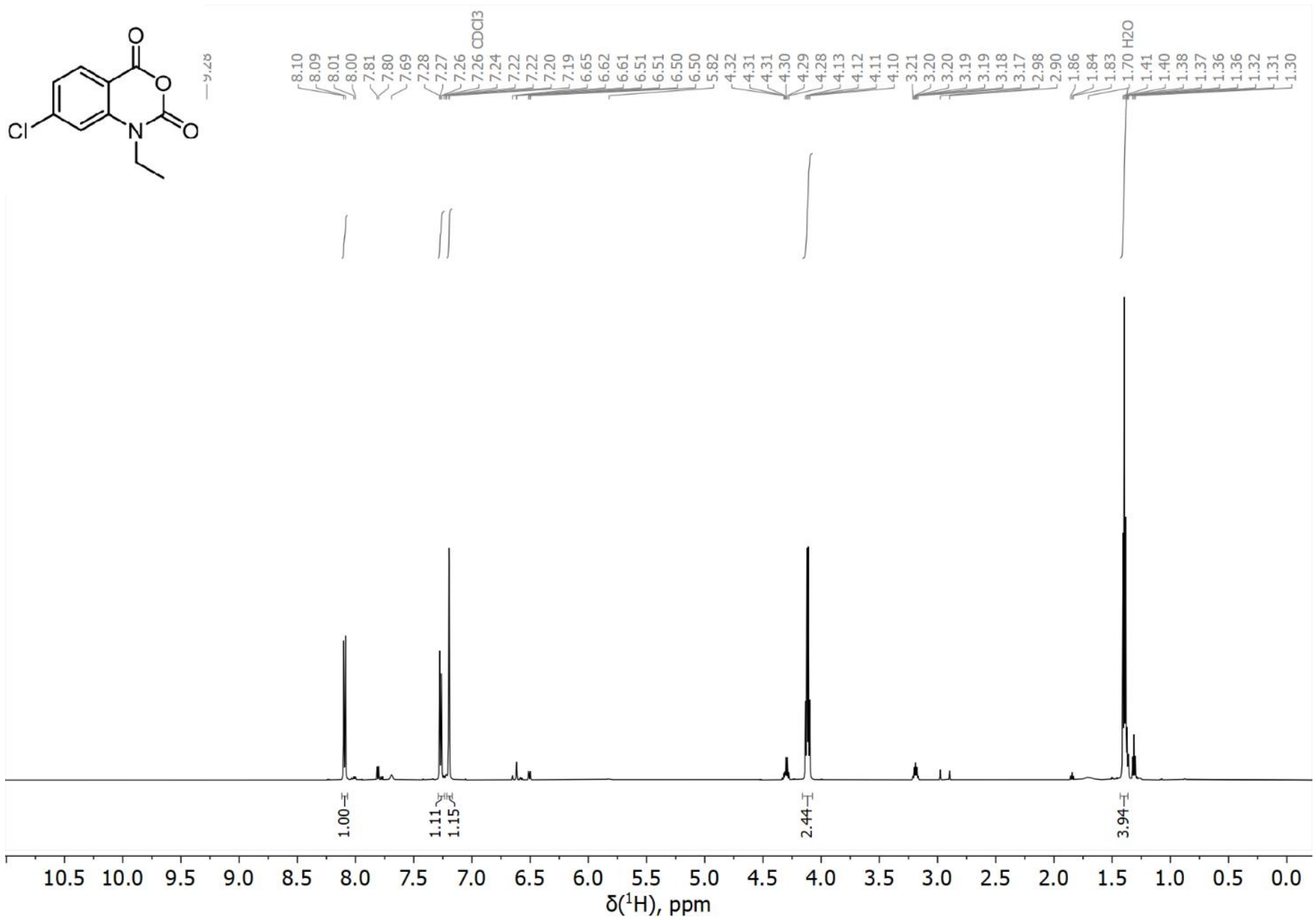

Supplementary Figure S20. ^{1}H and ^{13}C NMR spectrum of **4b**

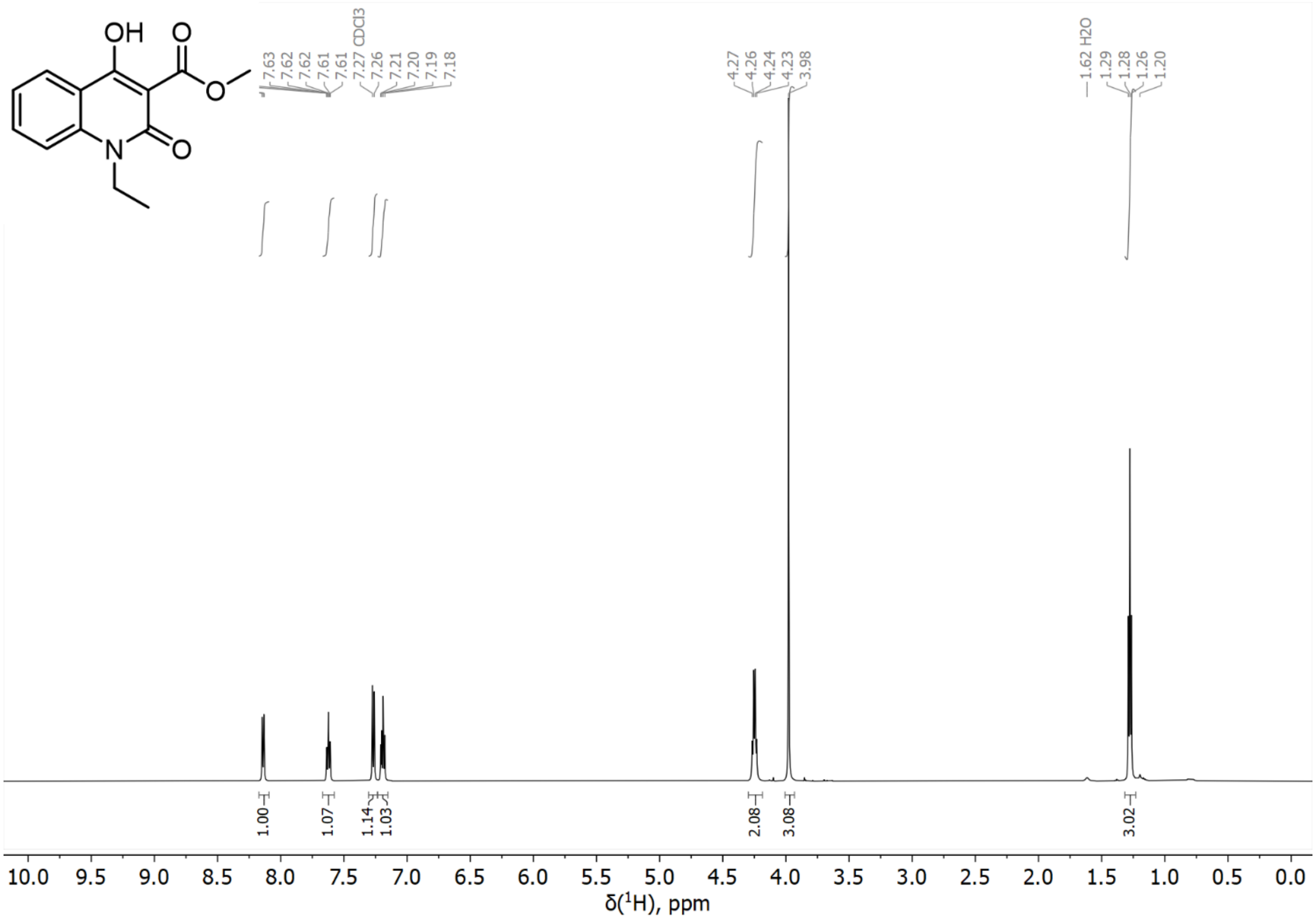

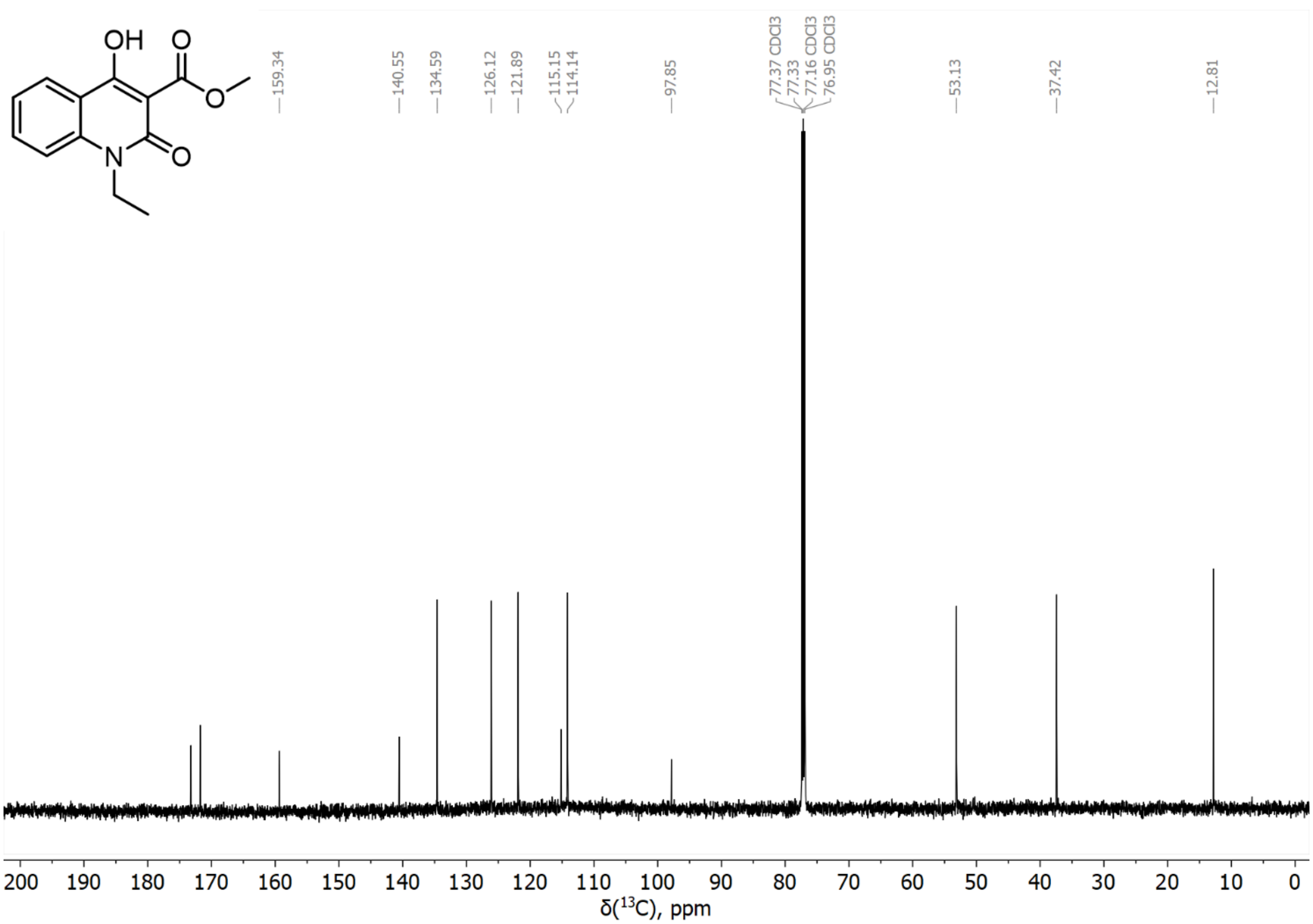

Supplementary Figure S21. ^{1}H and ^{13}C NMR spectrum of **5a**

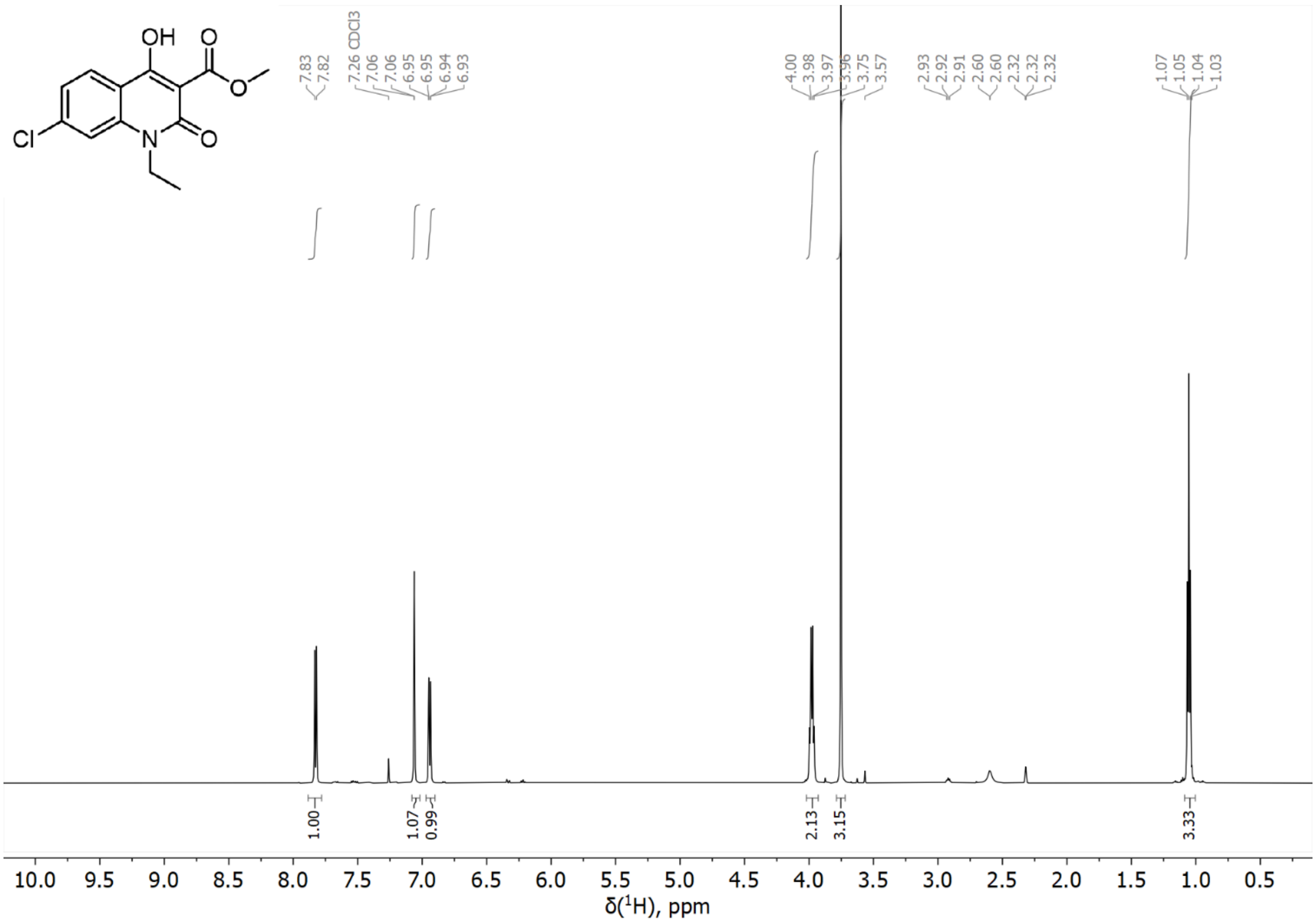

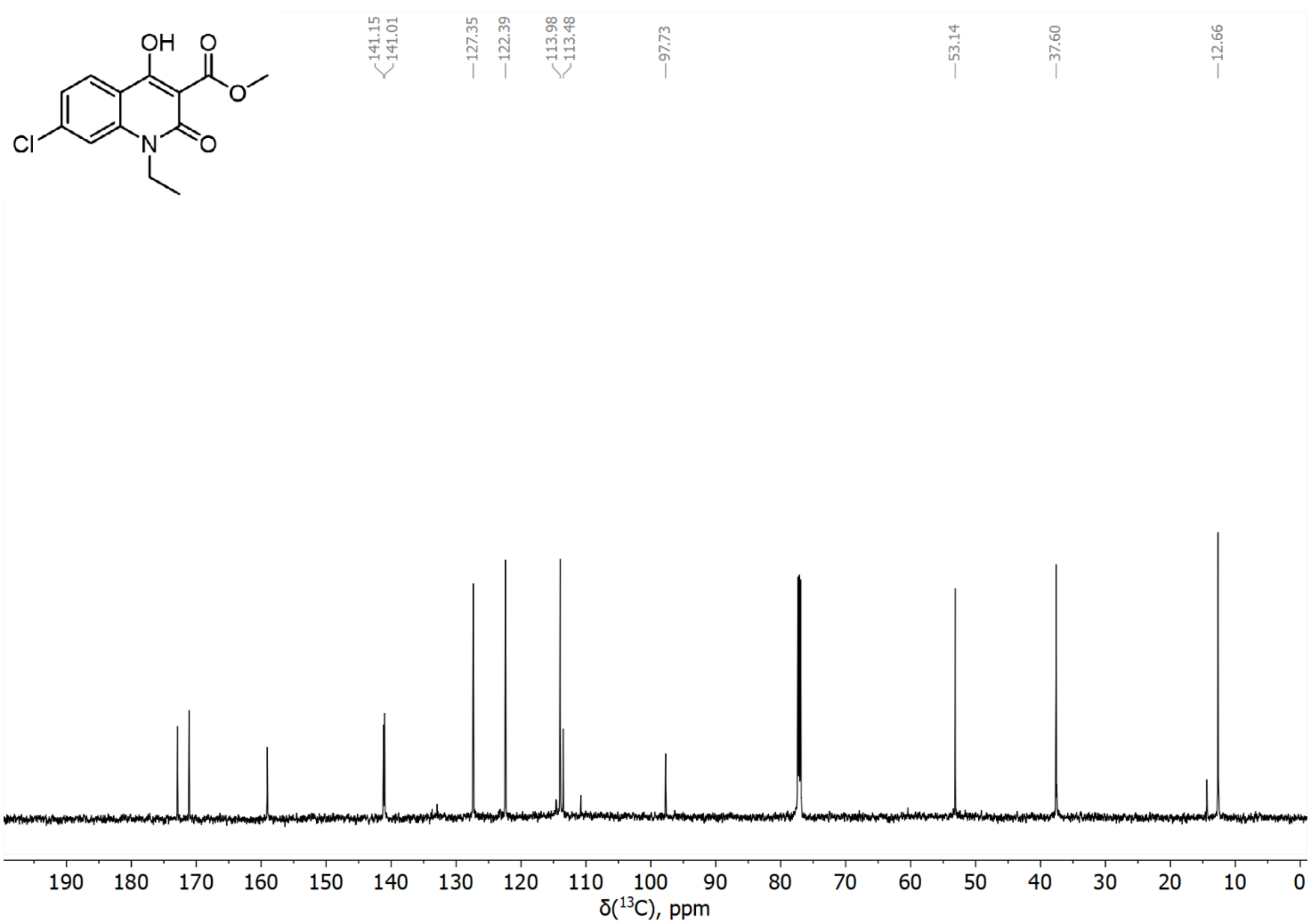

Supplementary Figure S22. ^{1}H and ^{13}C NMR spectrum of **5b**

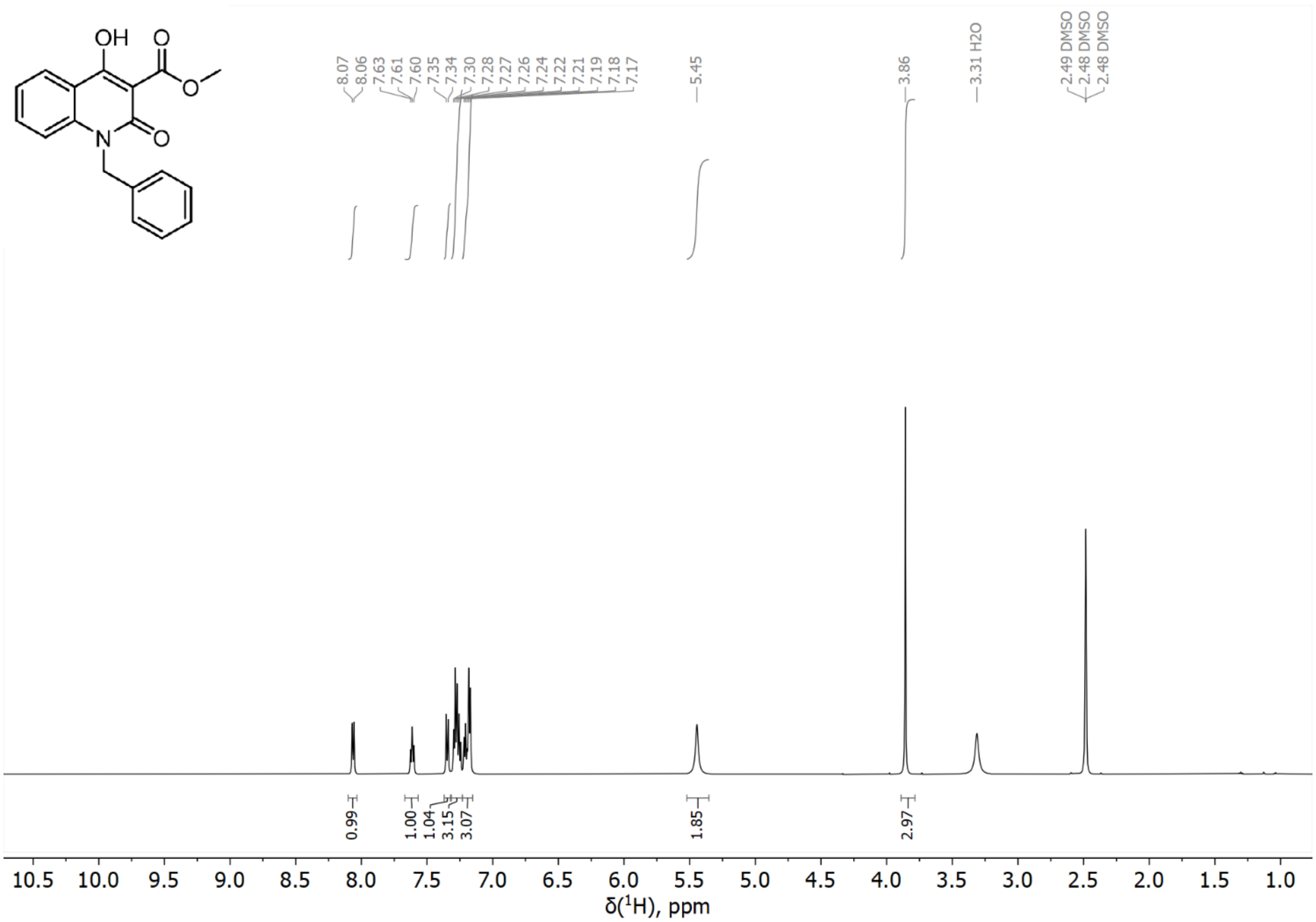

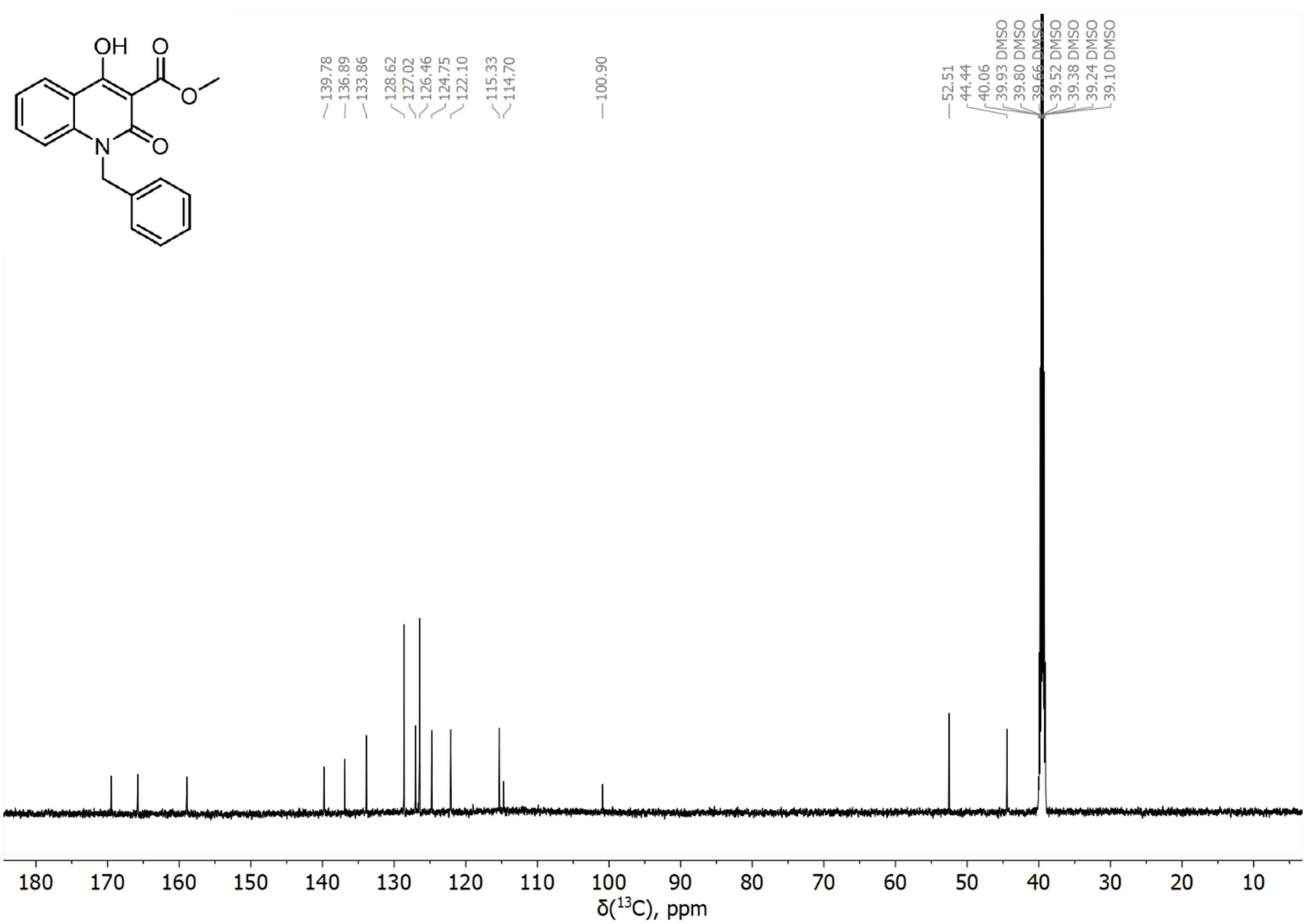

Supplementary Figure S23. ^{1}H and ^{13}C NMR spectrum of **5c**

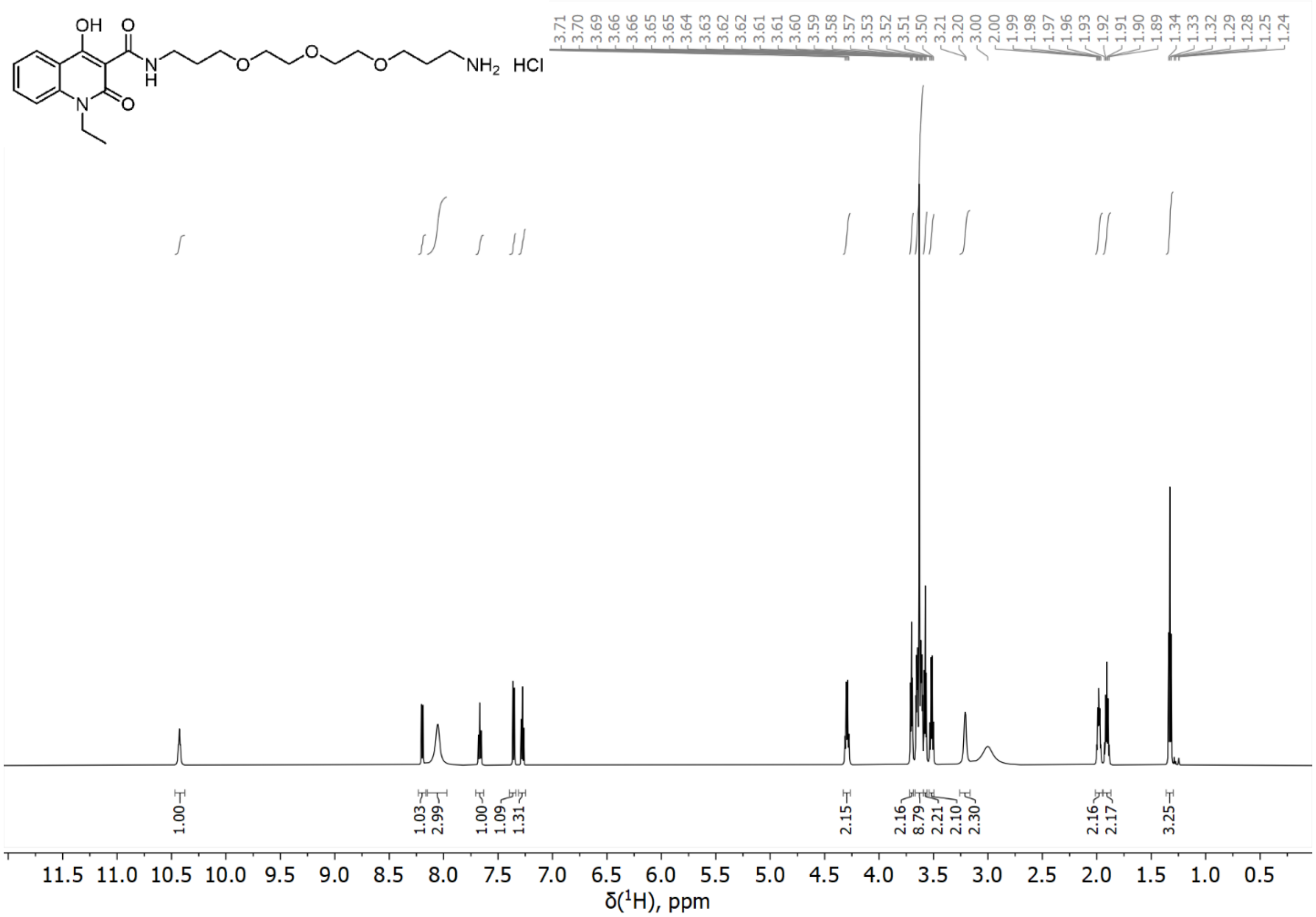

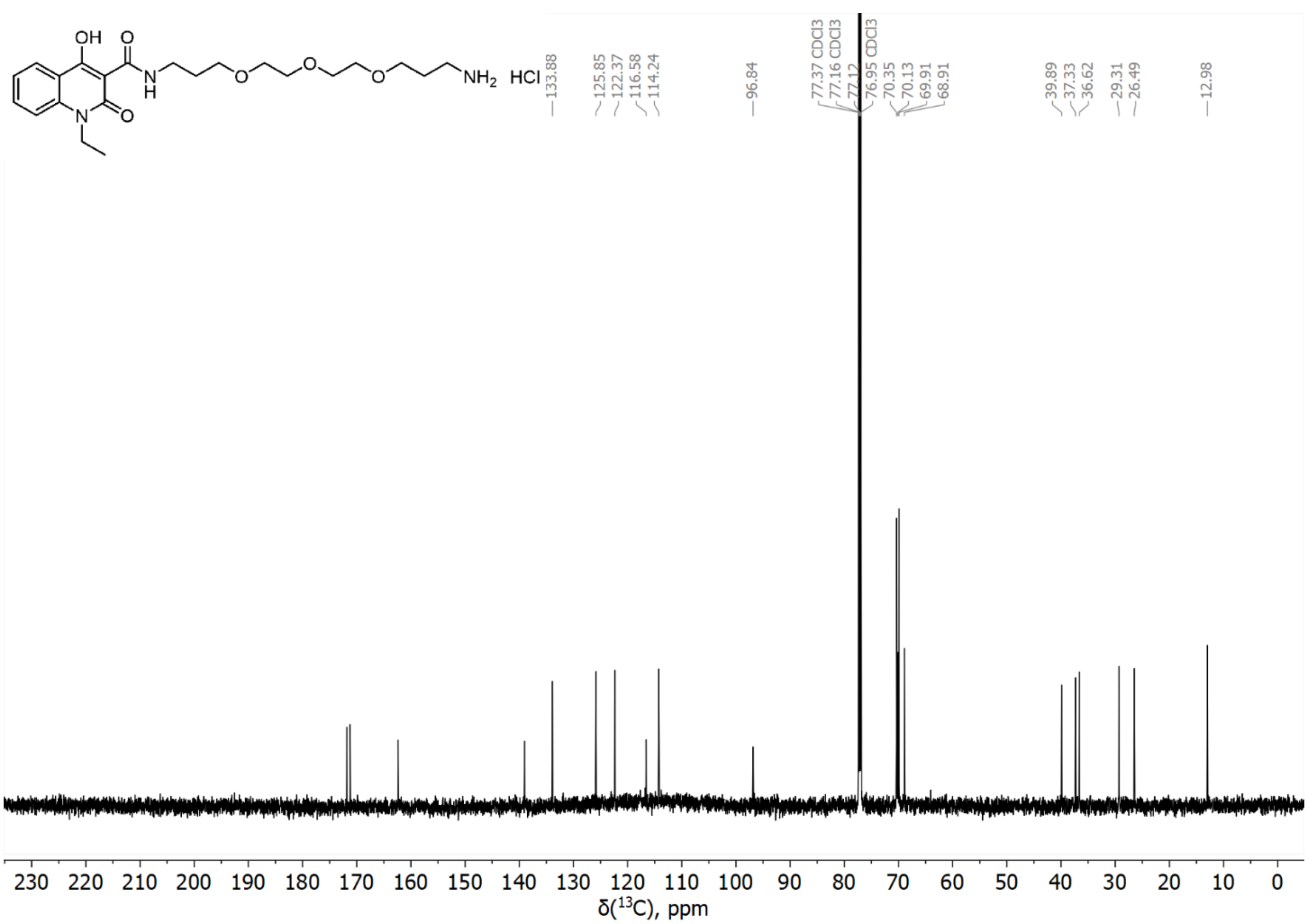

Supplementary Figure S24. ^{1}H and ^{13}C NMR spectrum of **6a**

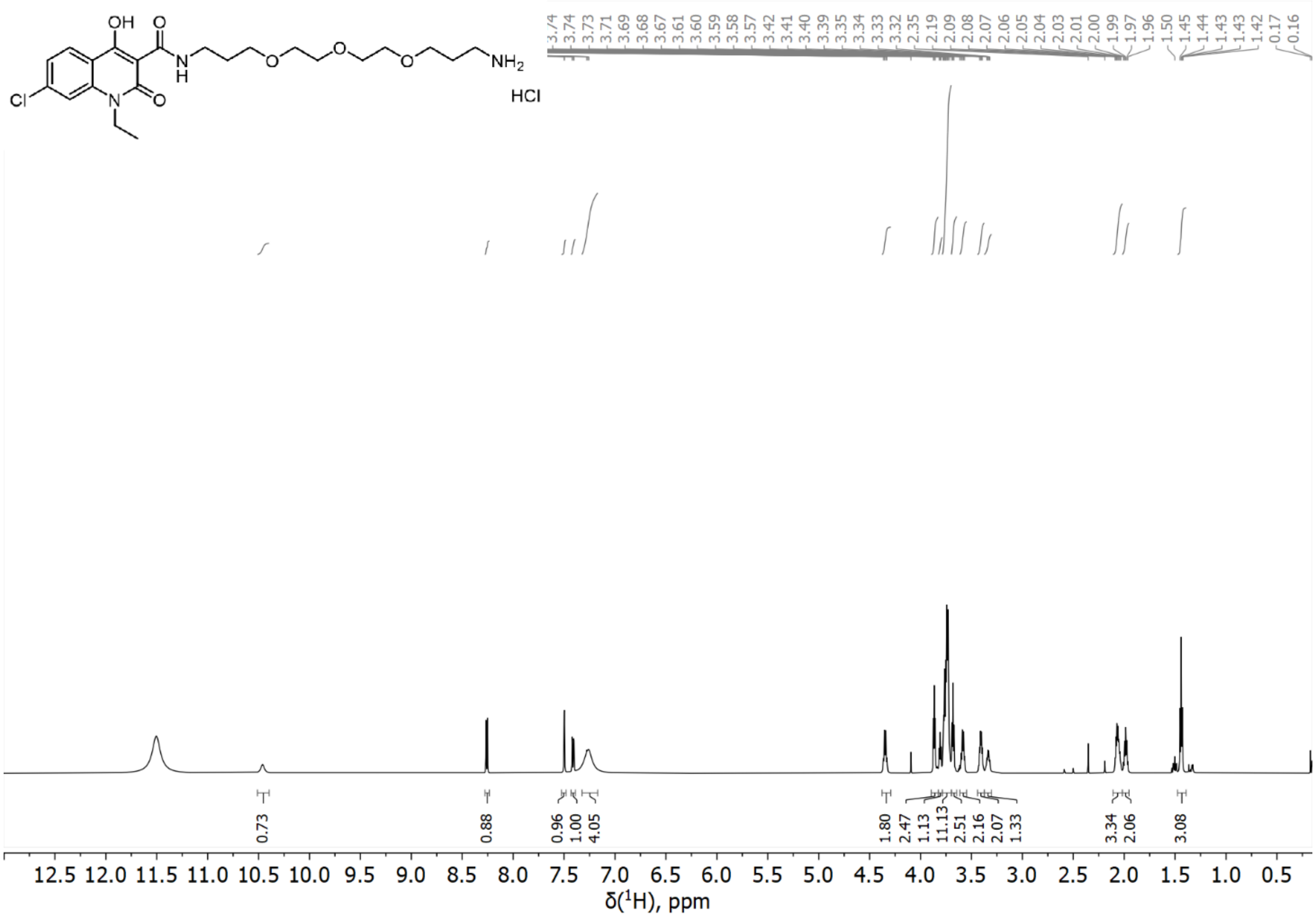

Supplementary Figure S25. ^{1}H and ^{13}C NMR spectrum of **6b**

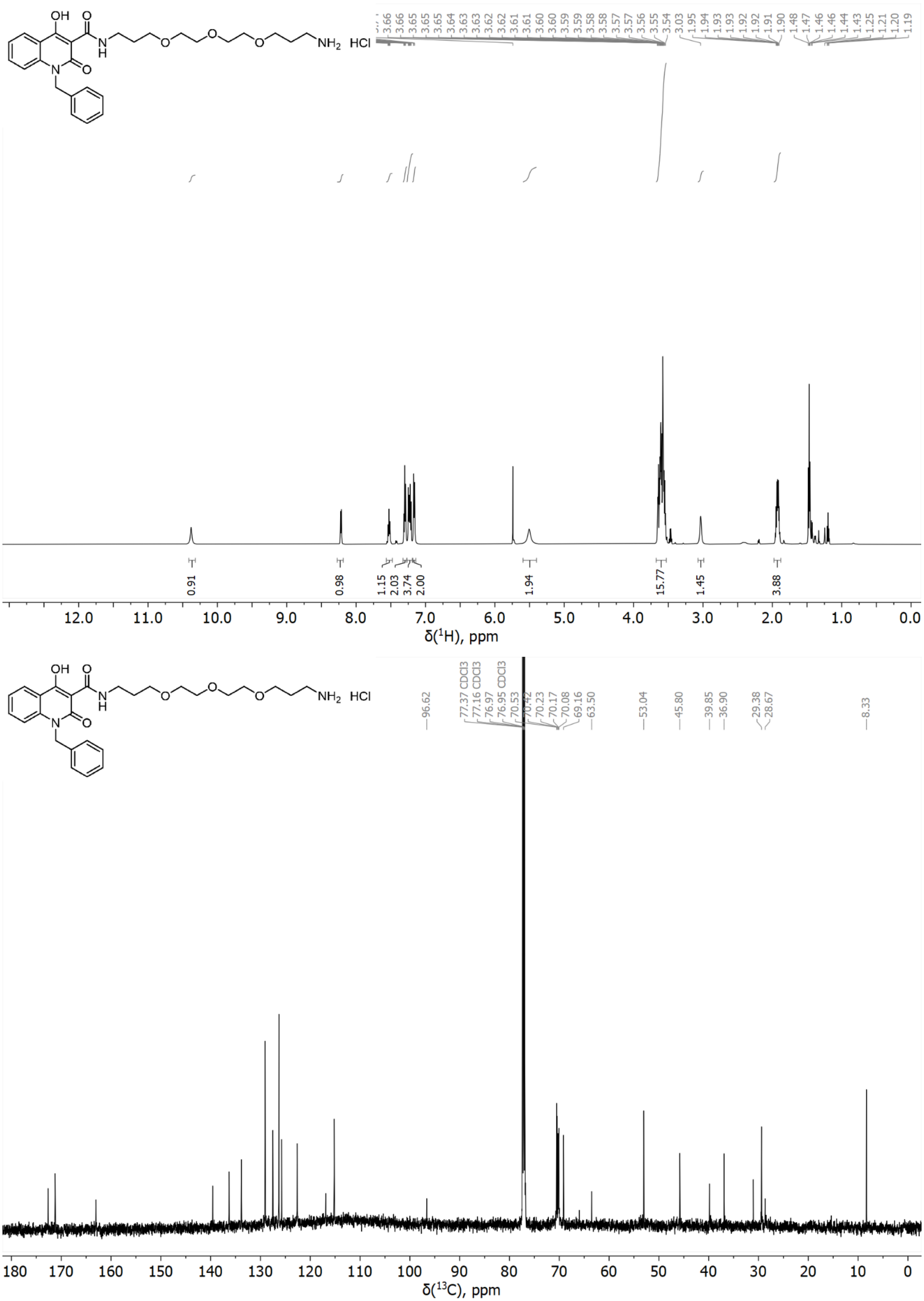

Supplementary Figure S26. ^{1}H and ^{13}C NMR spectrum of **6c**

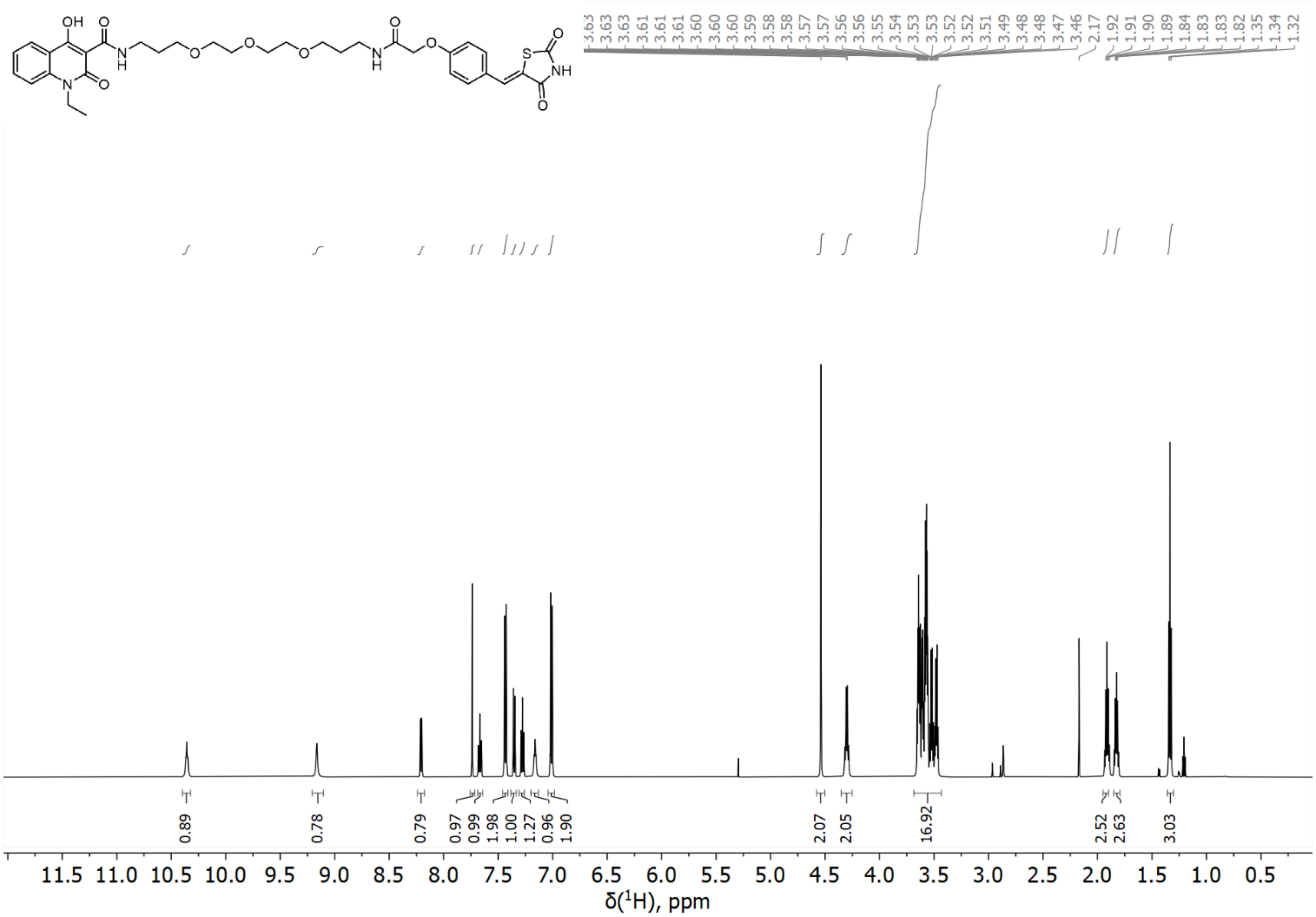

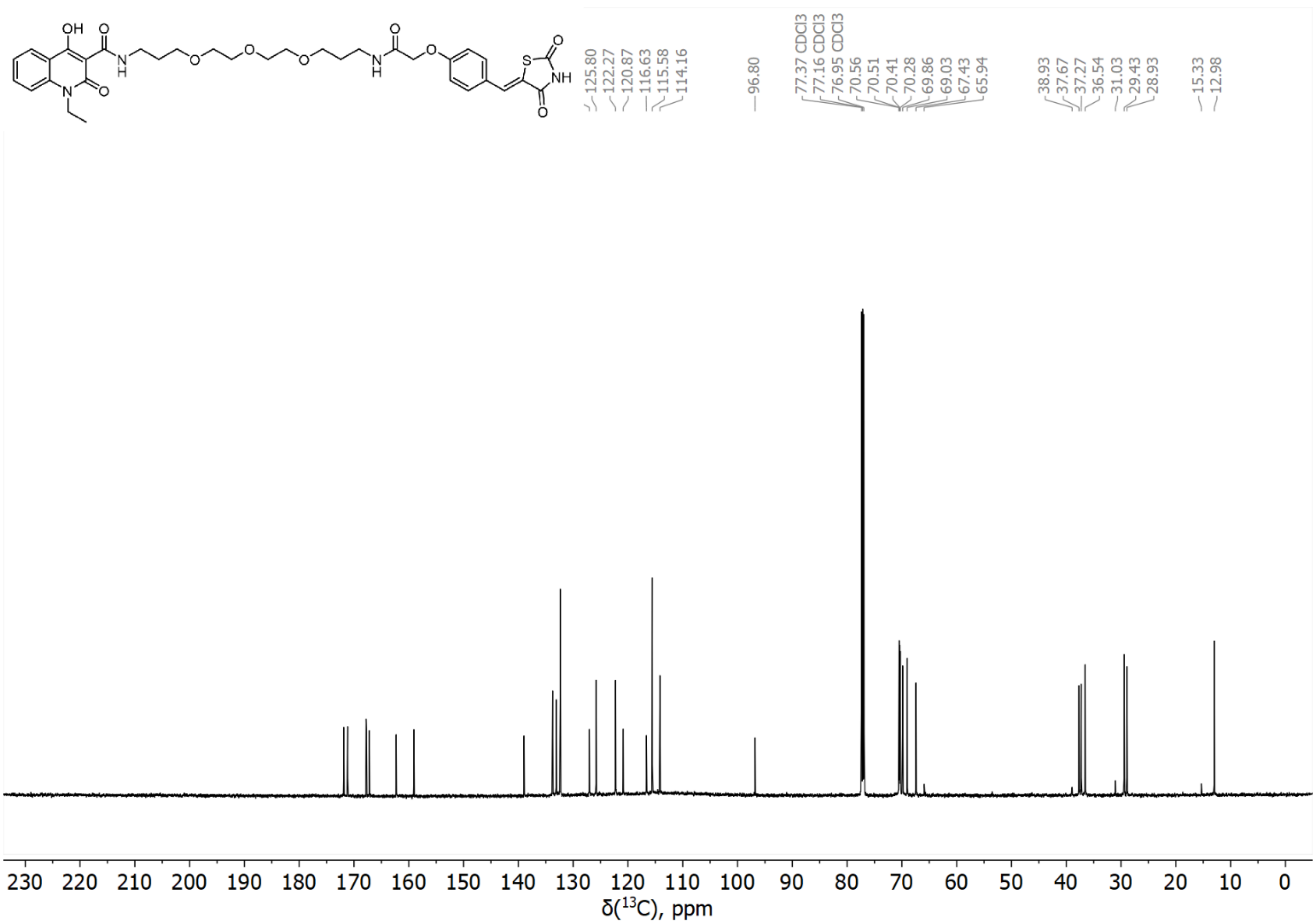

Supplementary Figure S27. ^{1}H and ^{13}C NMR spectrum of **MG-1**

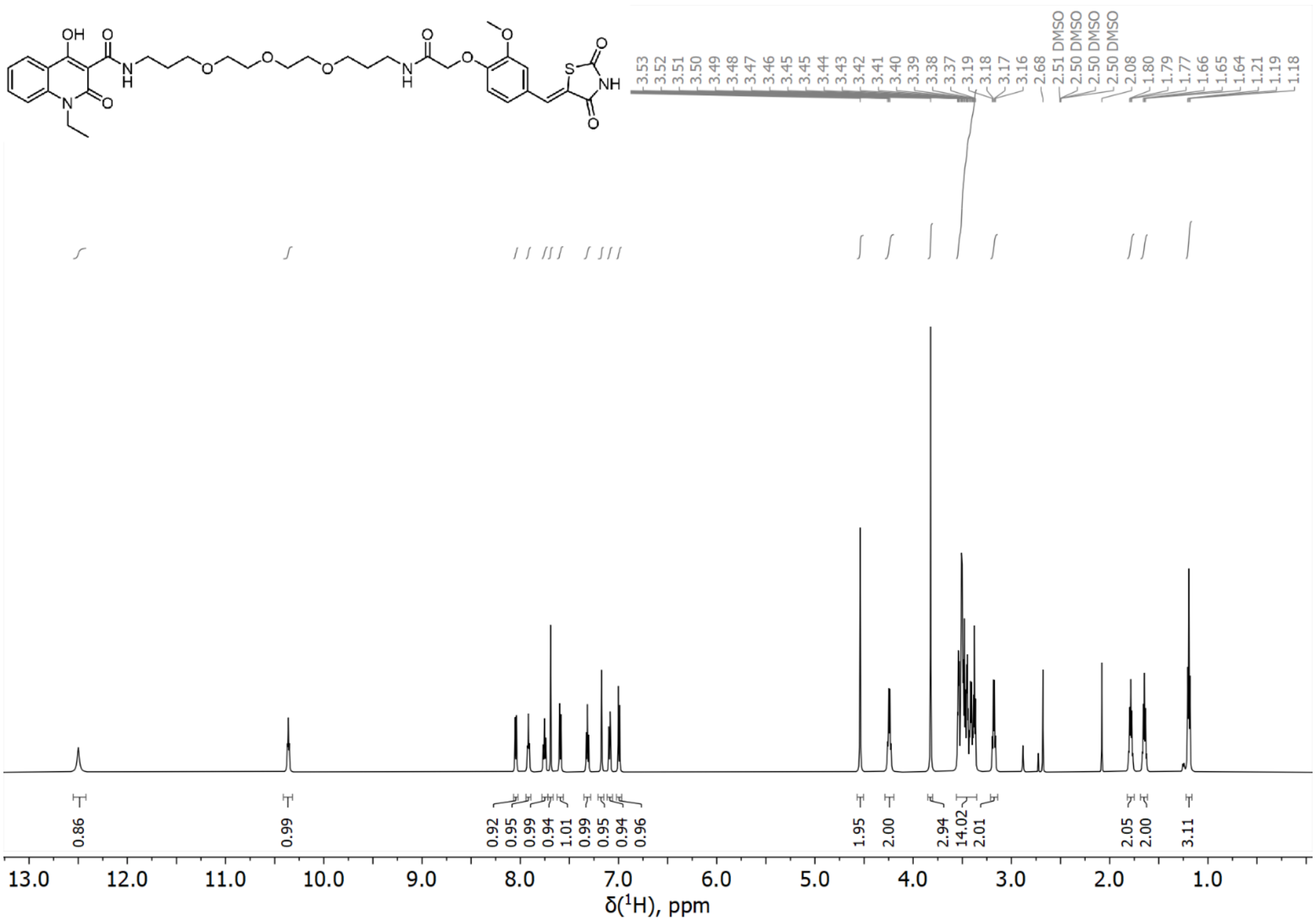

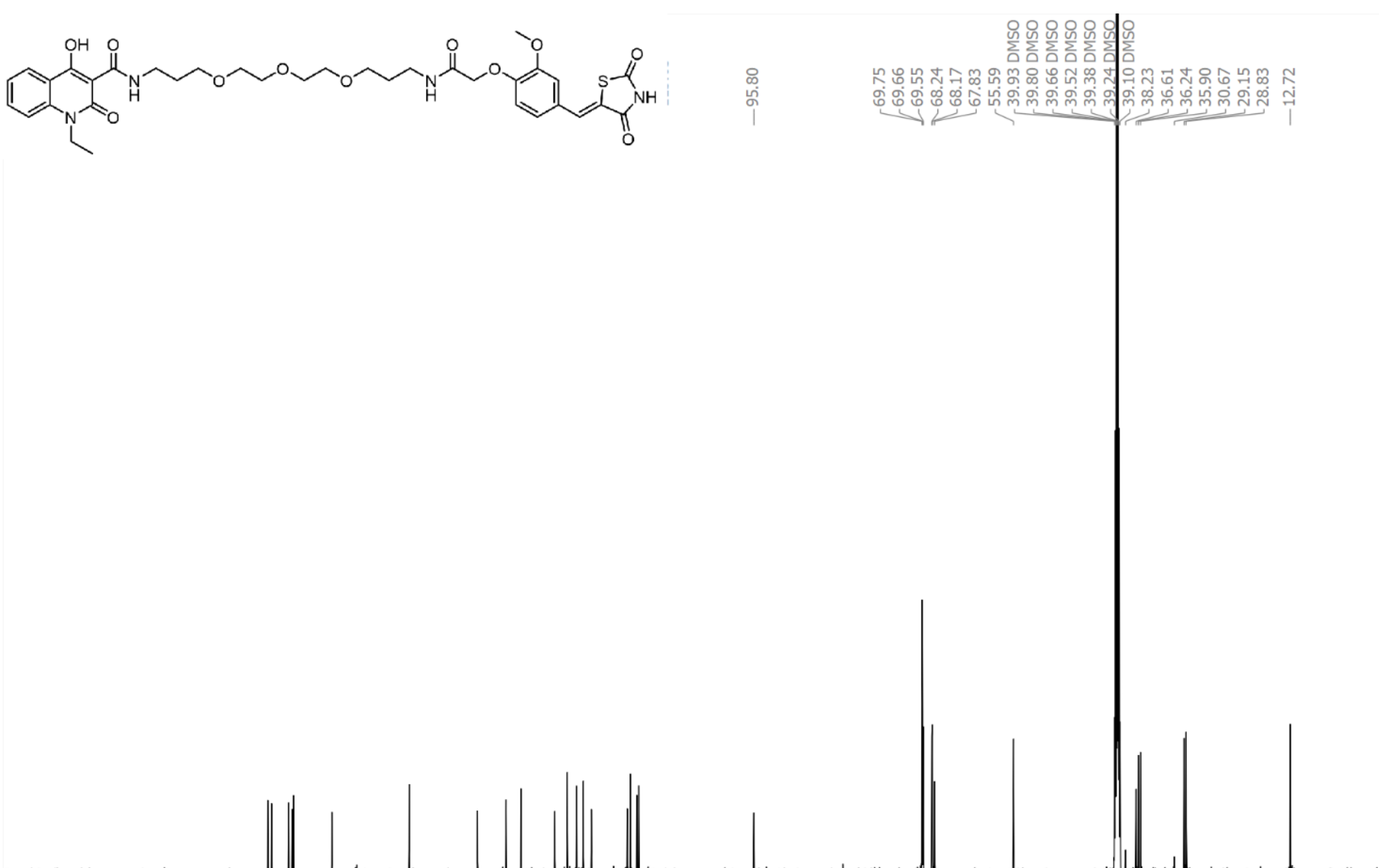

Supplementary Figure S28. ^{1}H and ^{13}C NMR spectrum of **MG-2**

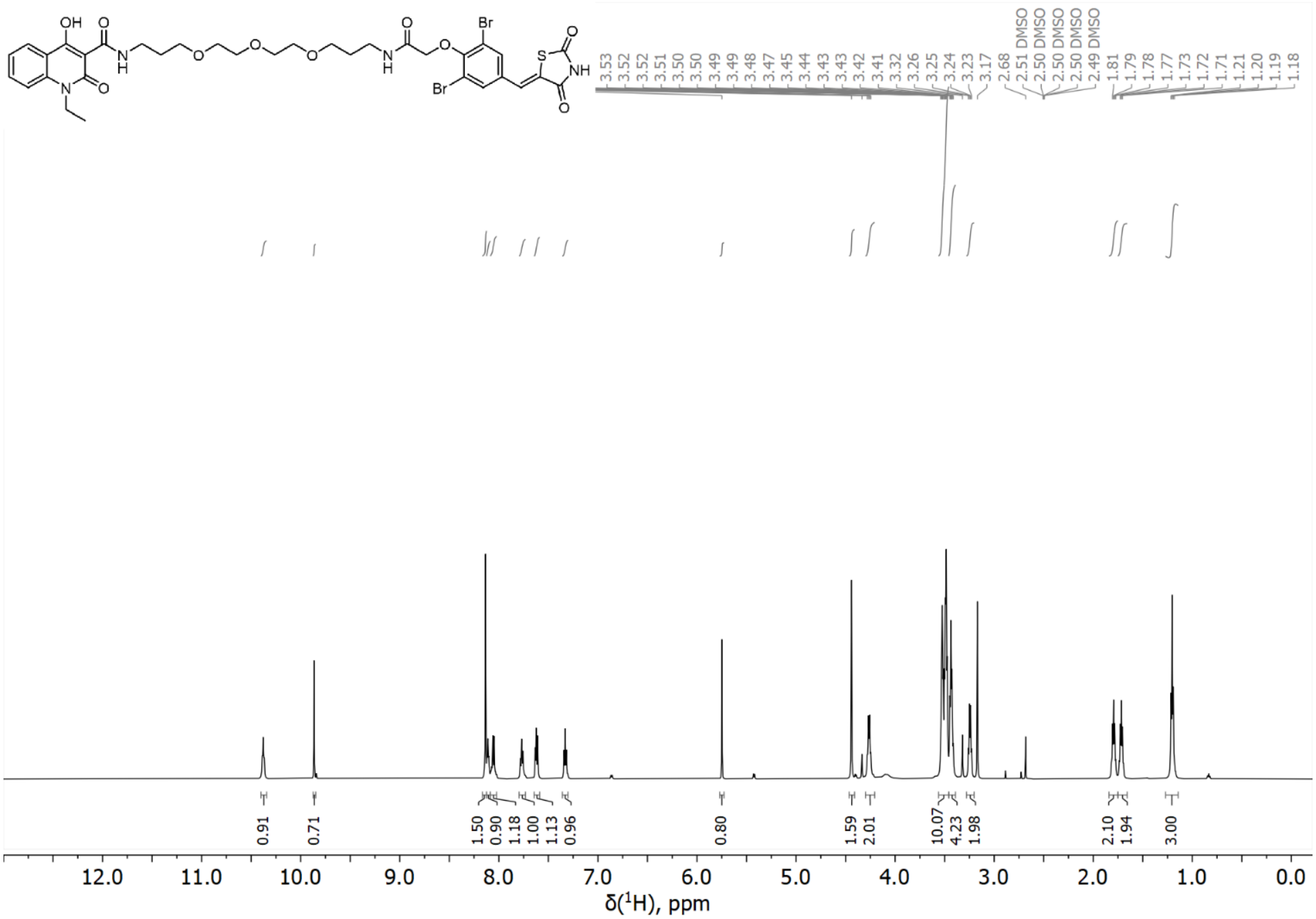

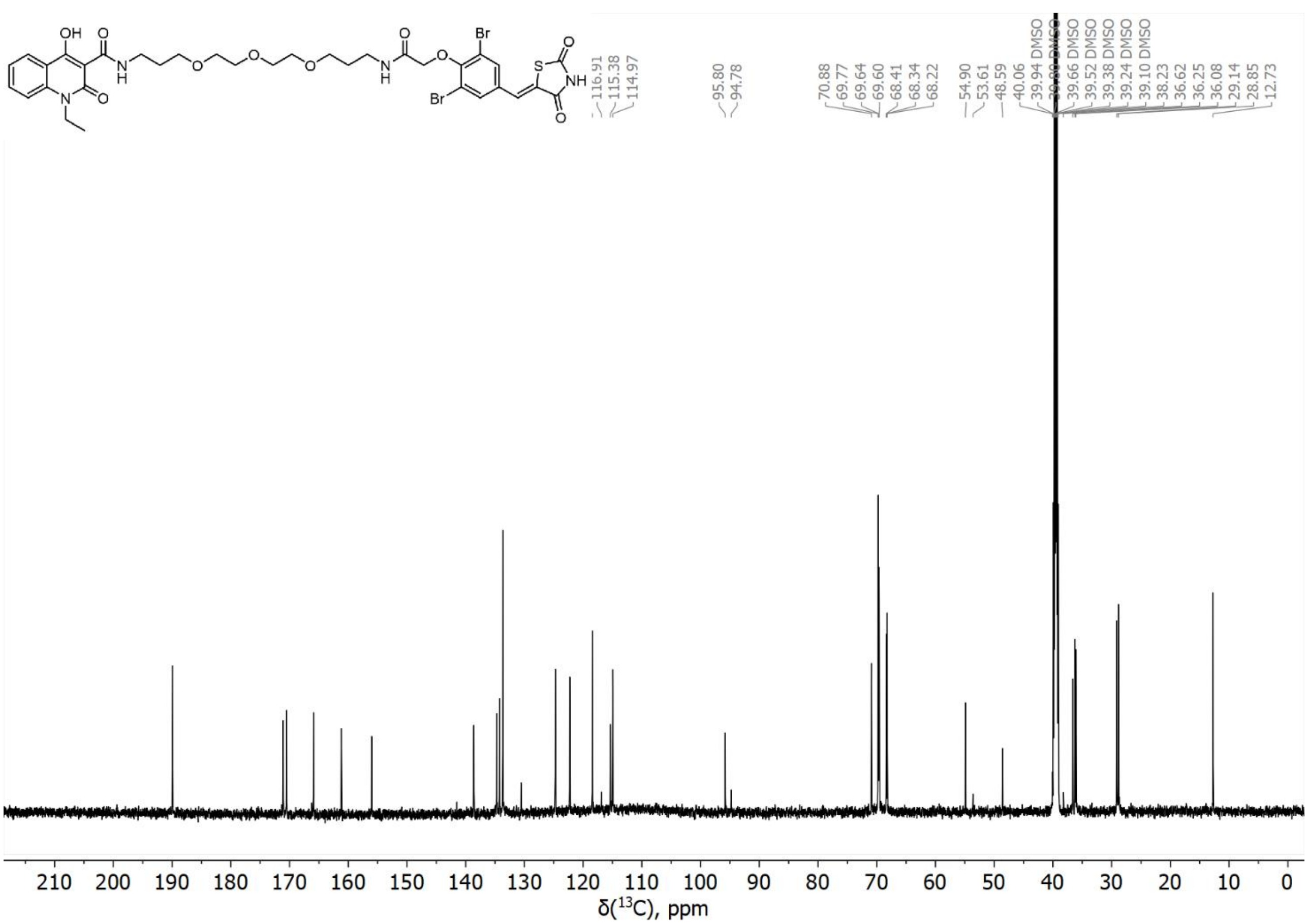

Supplementary Figure S29. ^{1}H and ^{13}C NMR spectrum of **MG-3**

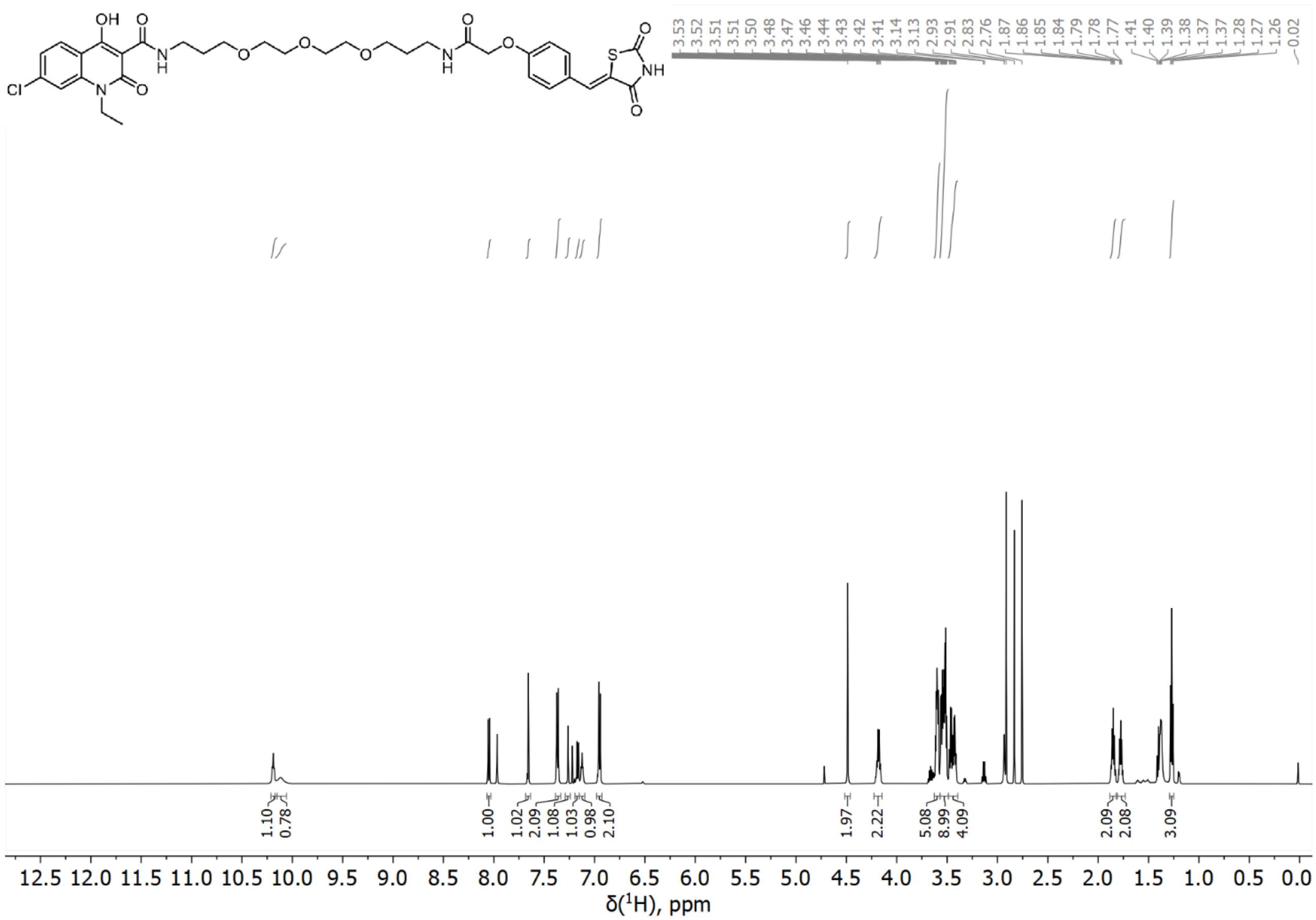

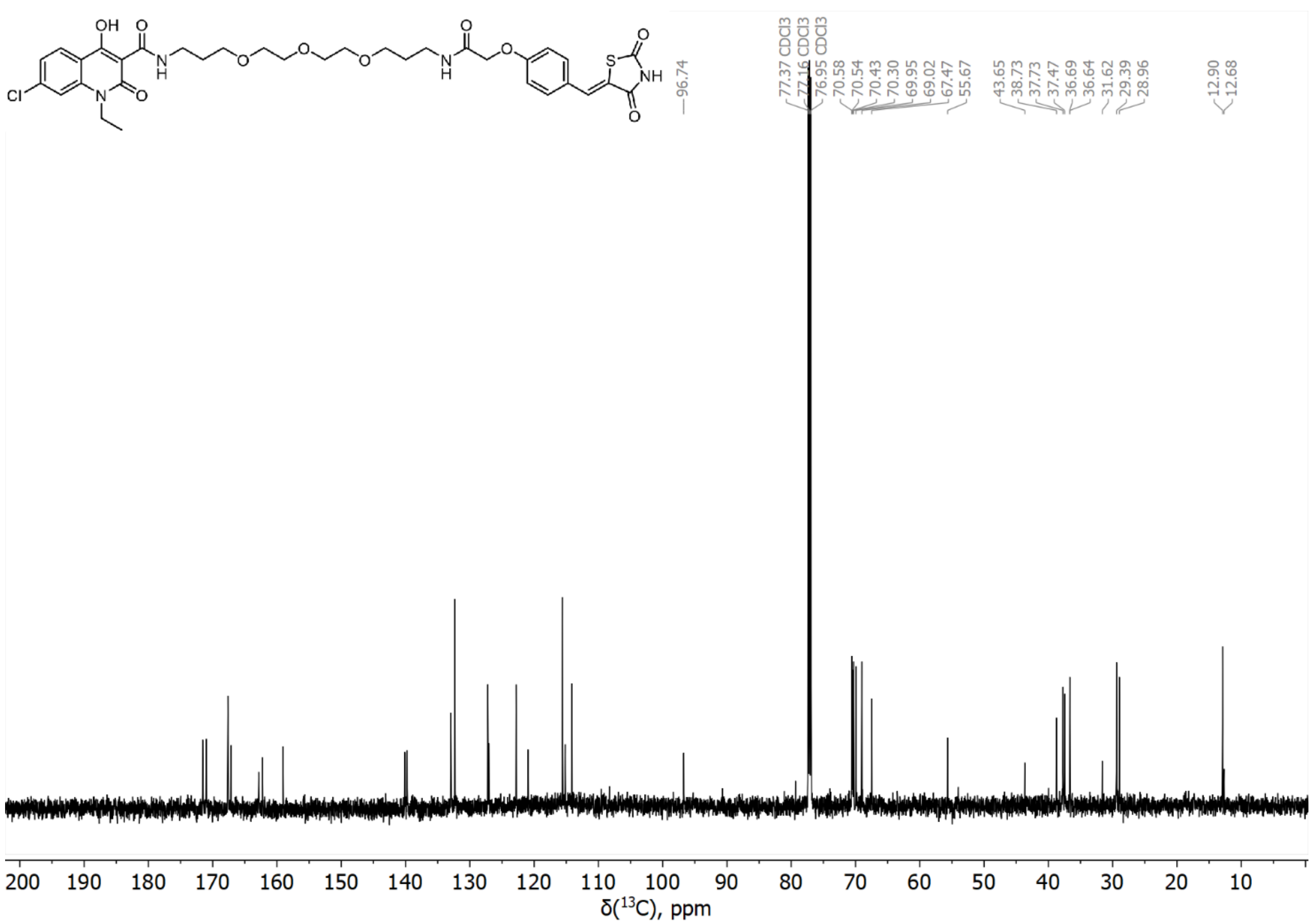

Supplementary Figure S30. ^{1}H and ^{13}C NMR spectrum of **MG-4**

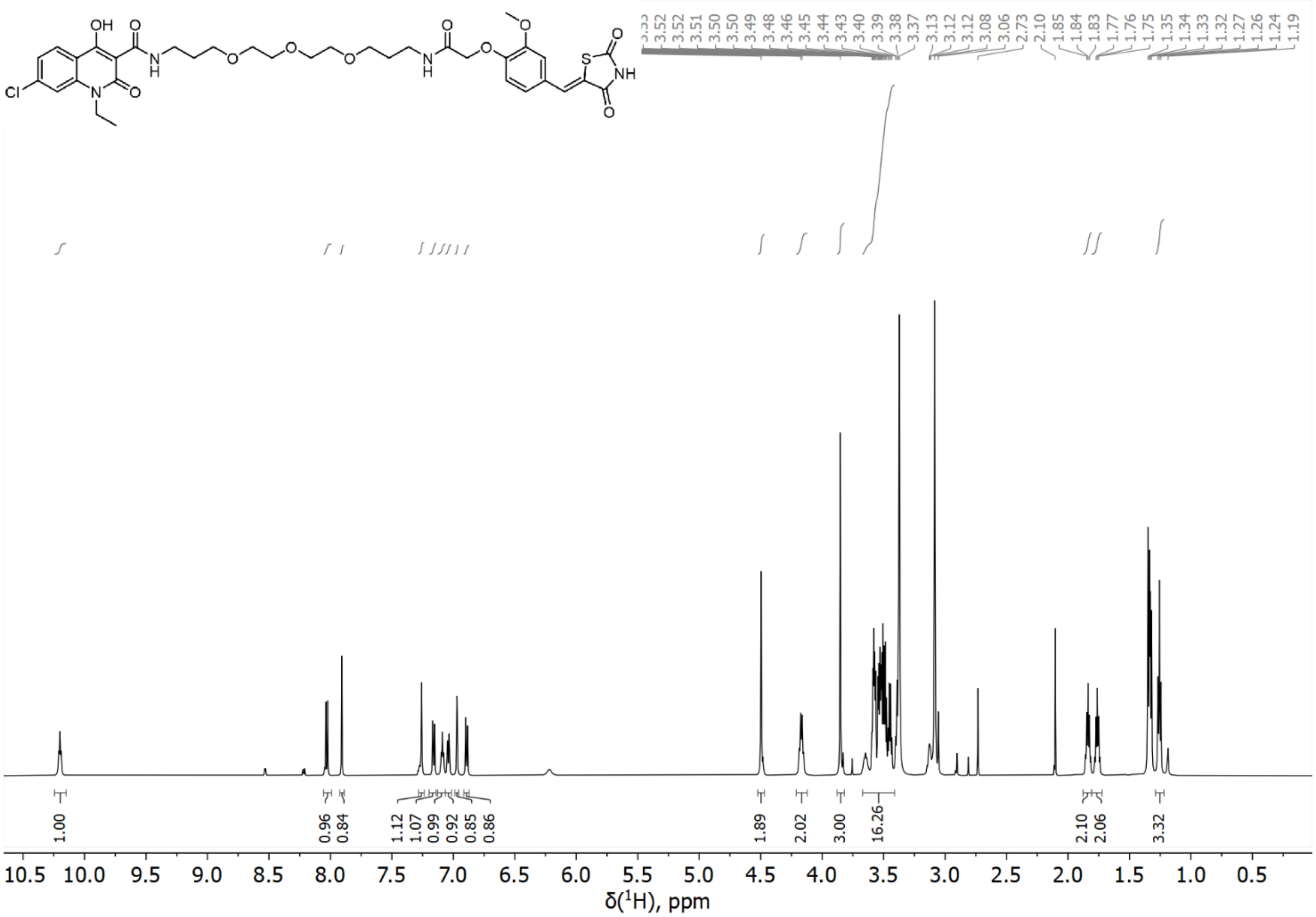

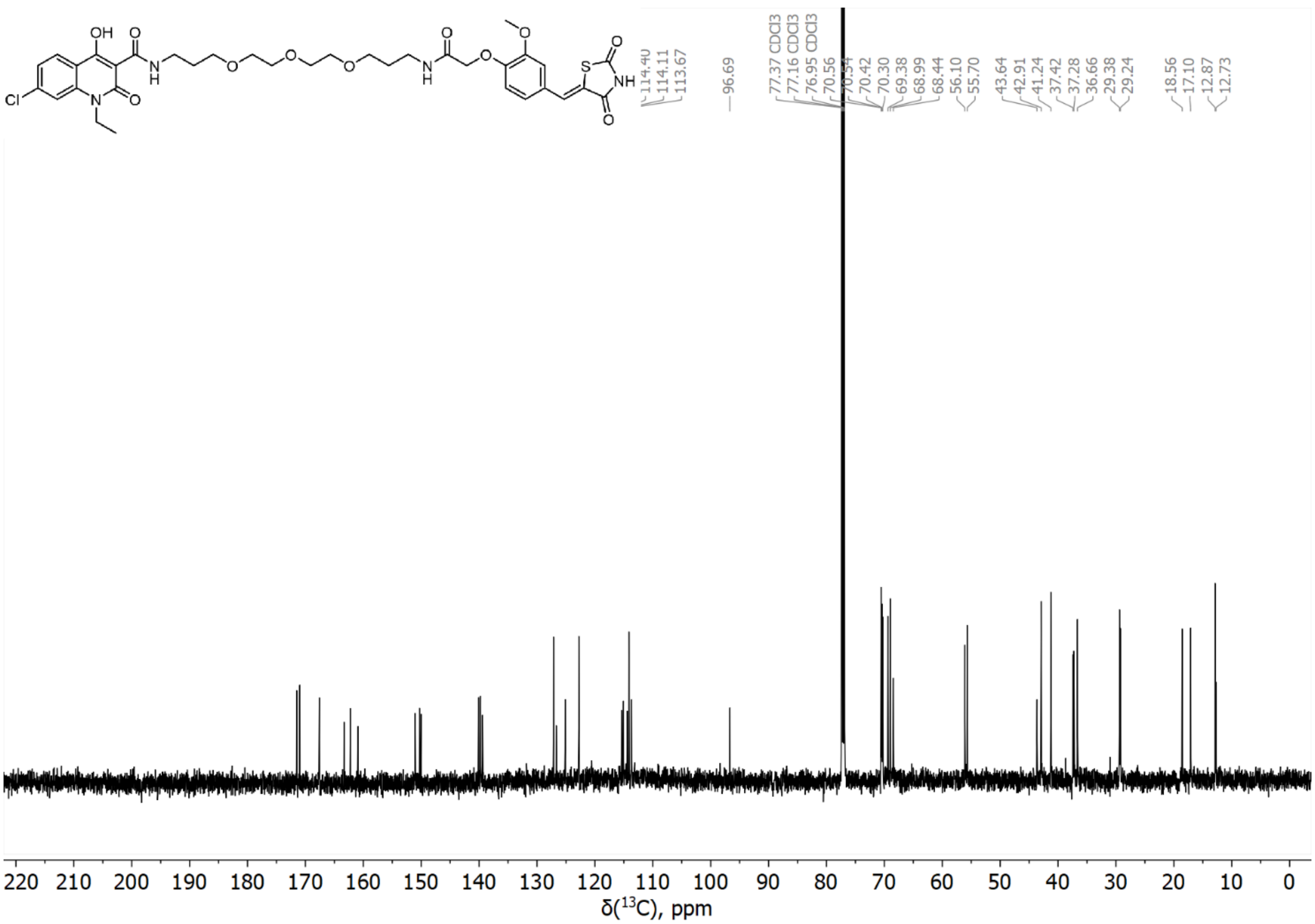

Supplementary Figure S31. ^{1}H and ^{13}C NMR spectrum of **MG-5**

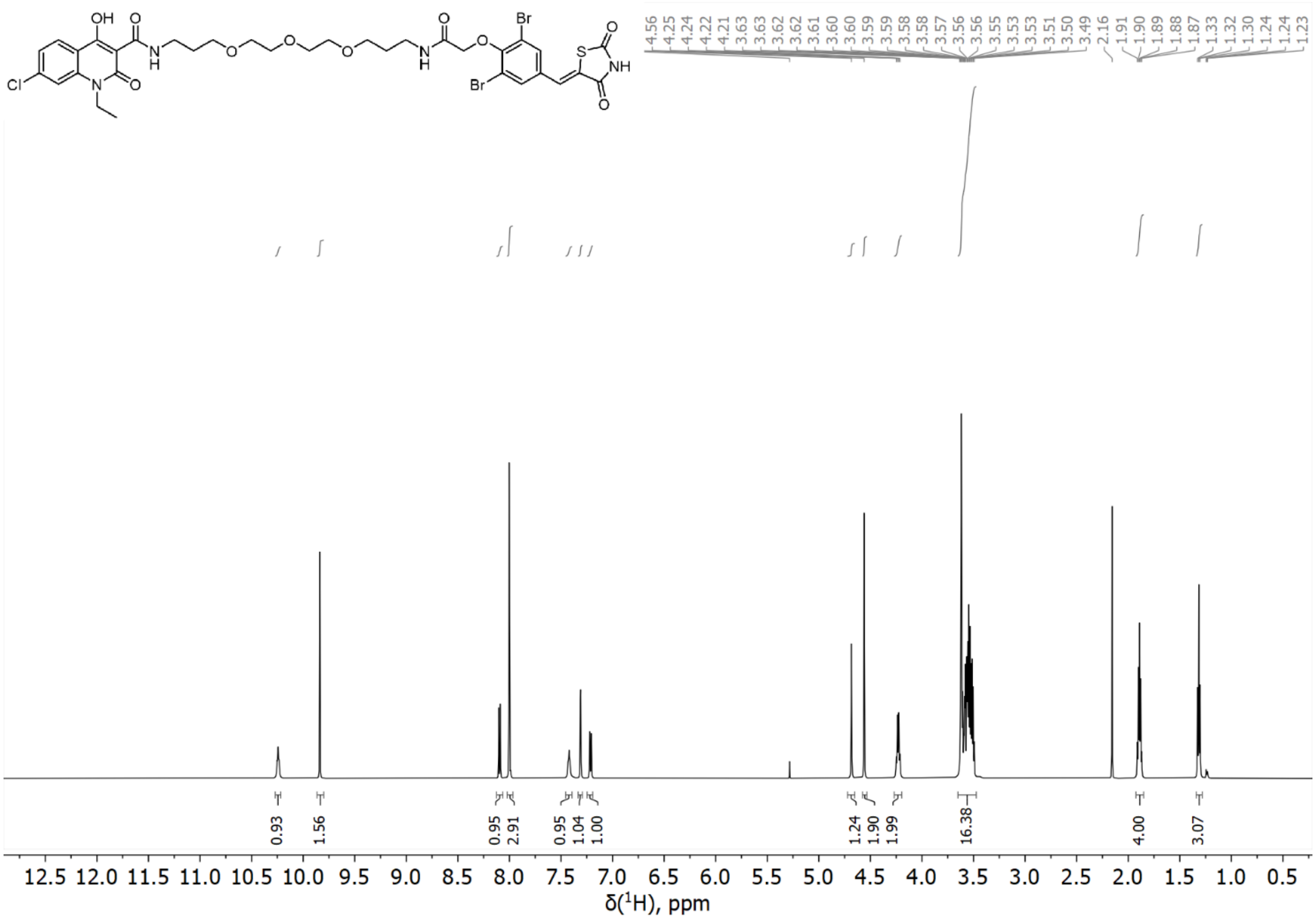

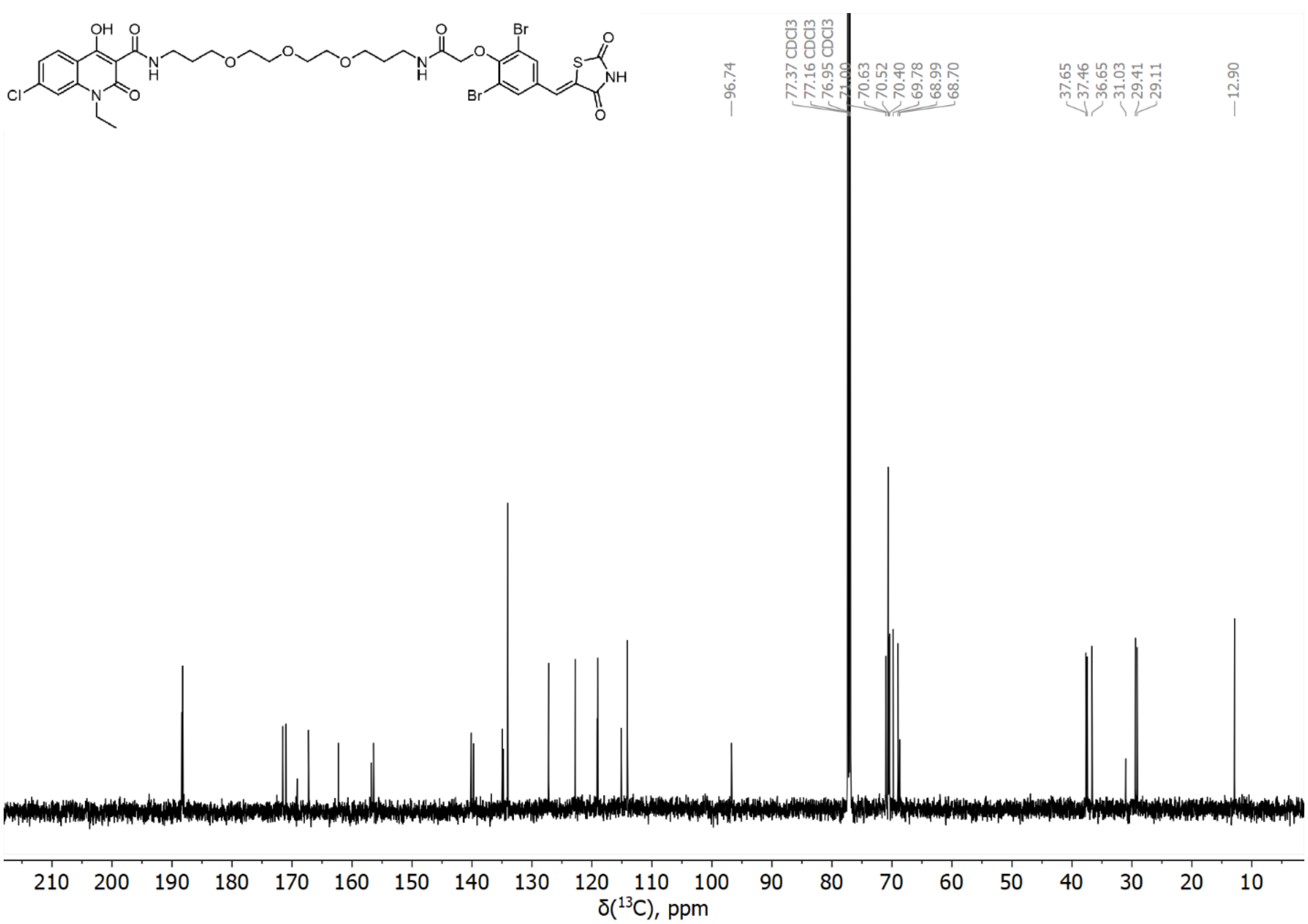

Supplementary Figure S32. ^{1}H and ^{13}C NMR spectrum of **MG-6**

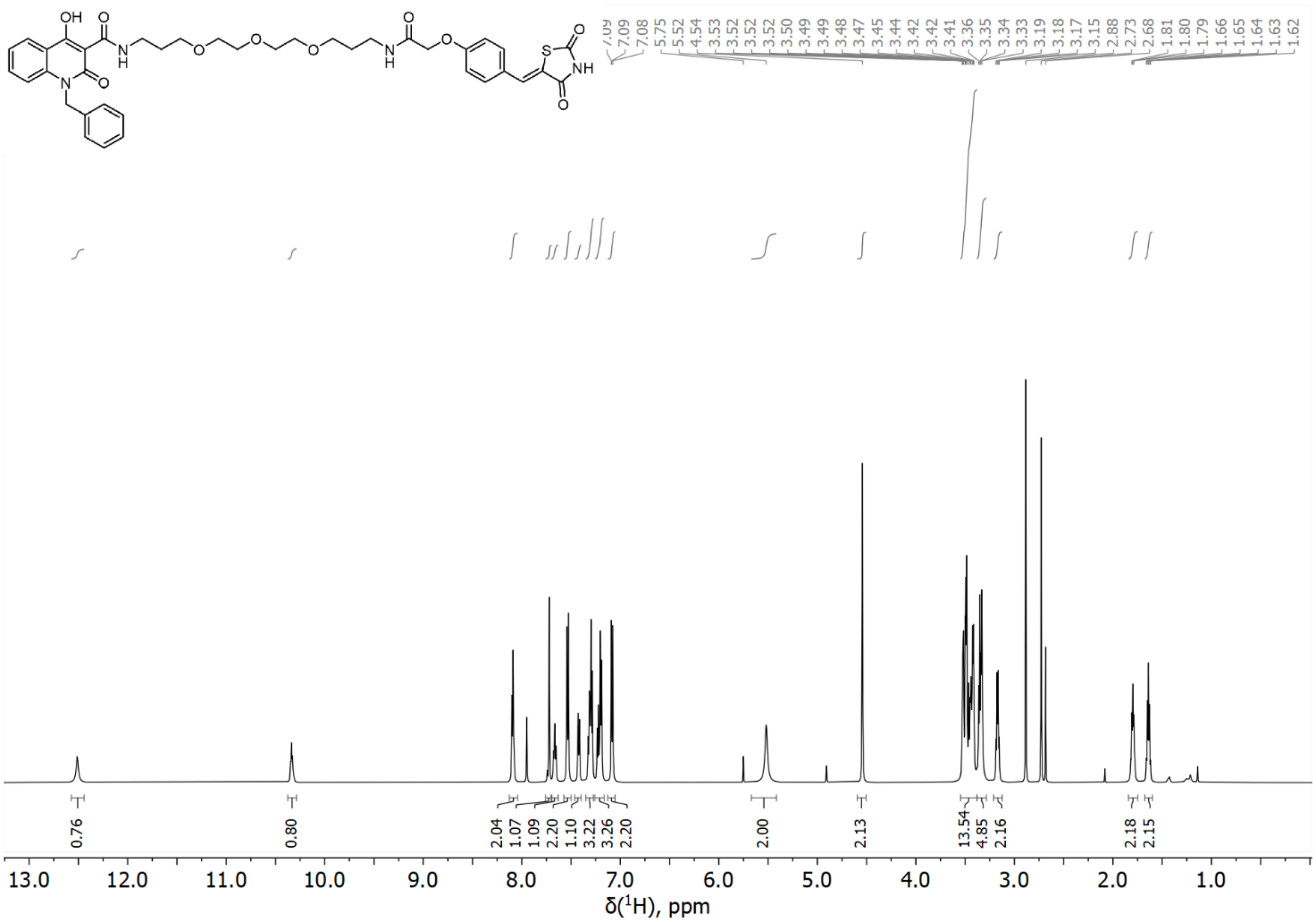

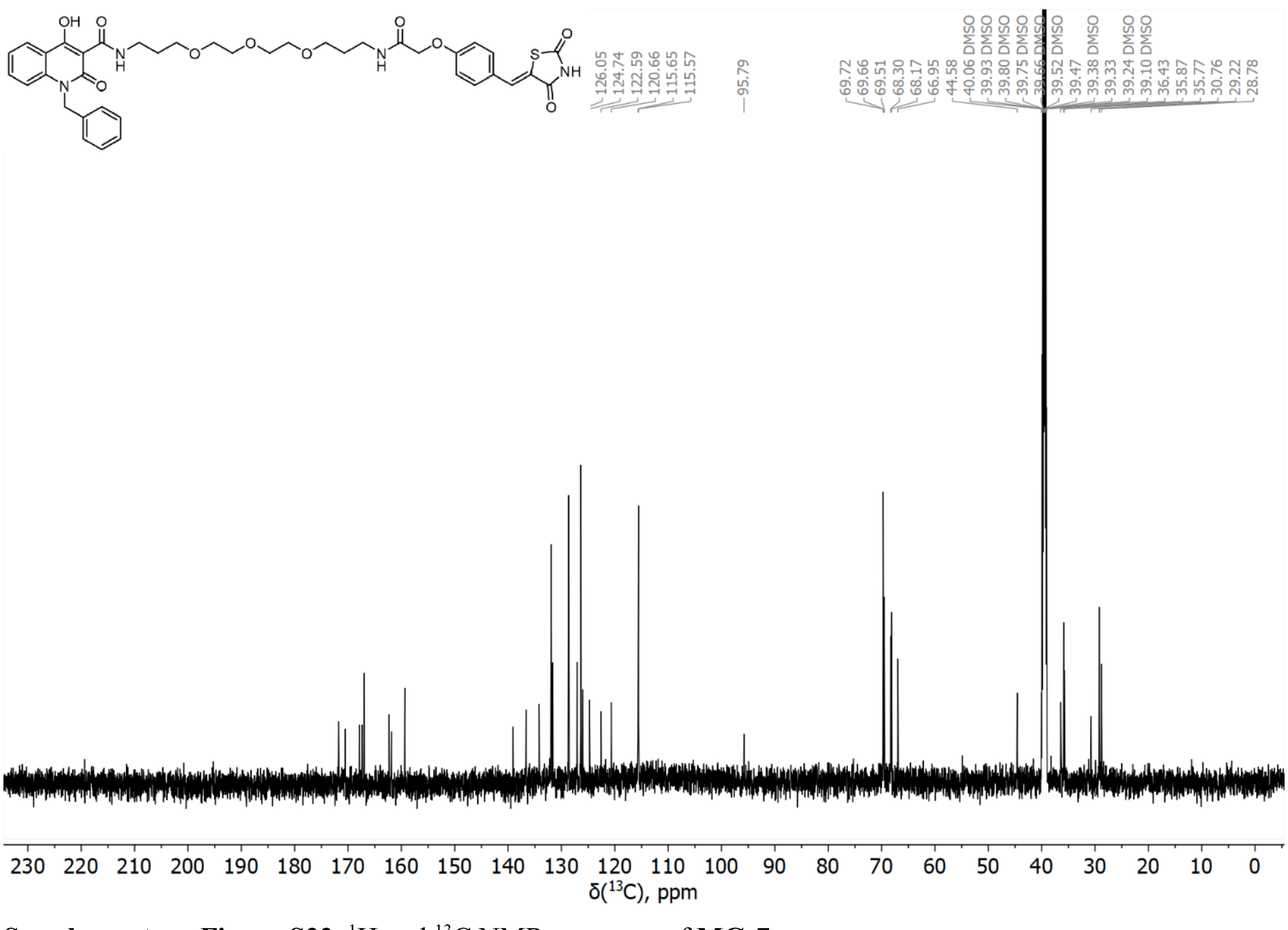

Supplementary Figure S33. ^{1}H and ^{13}C NMR spectrum of **MG-7**

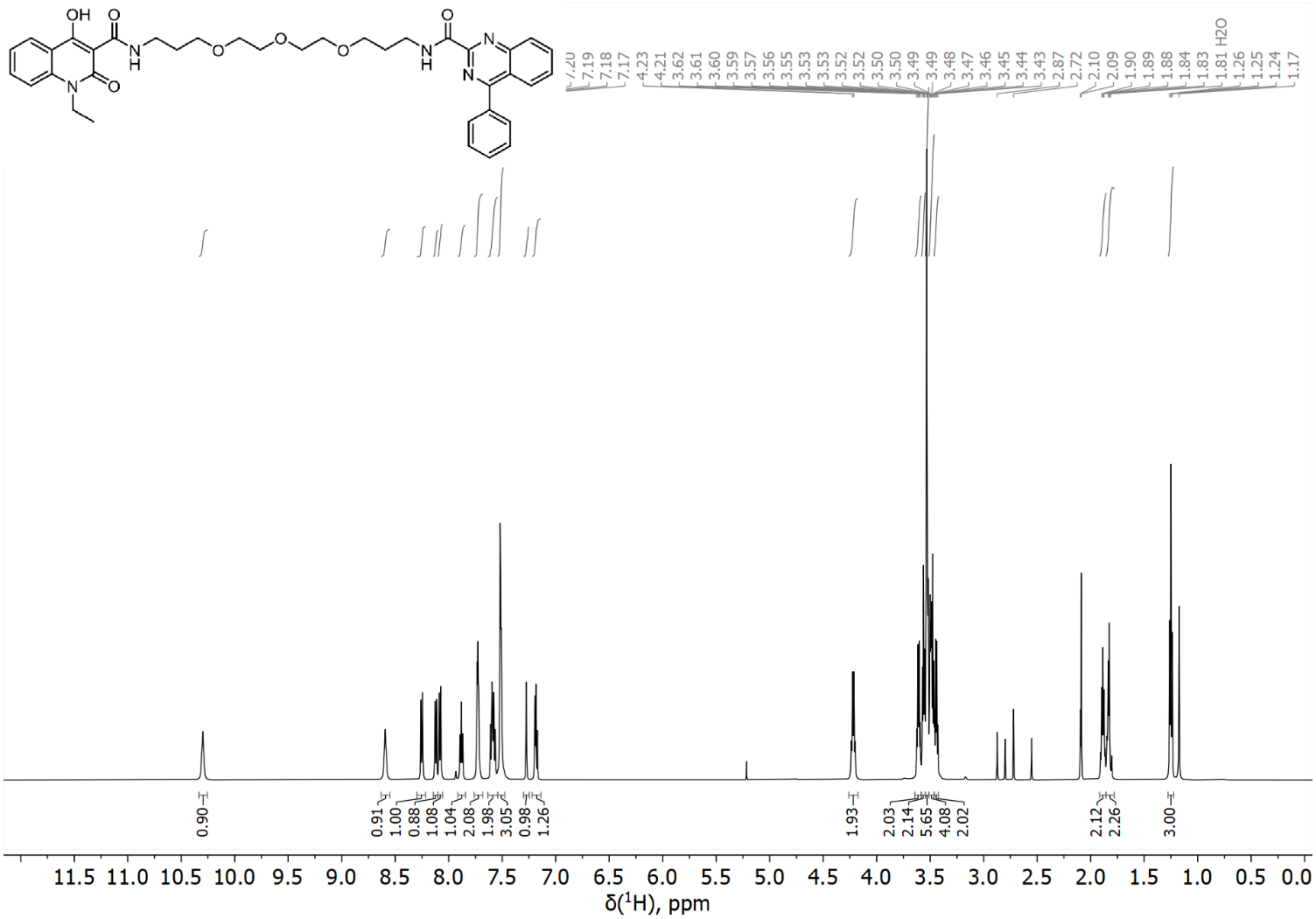

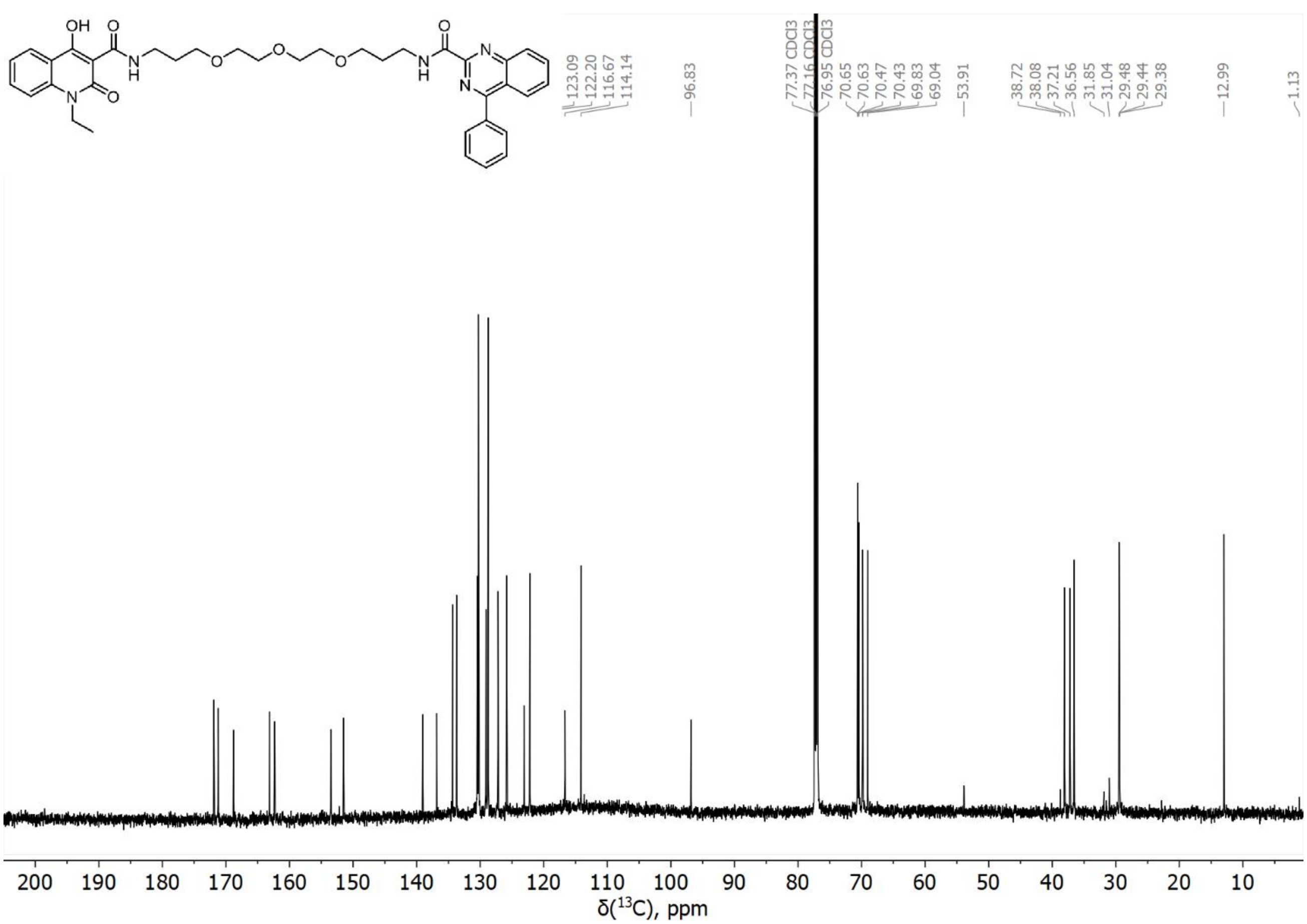

Supplementary Figure S34. ^{1}H and ^{13}C NMR spectrum of **MG-8**

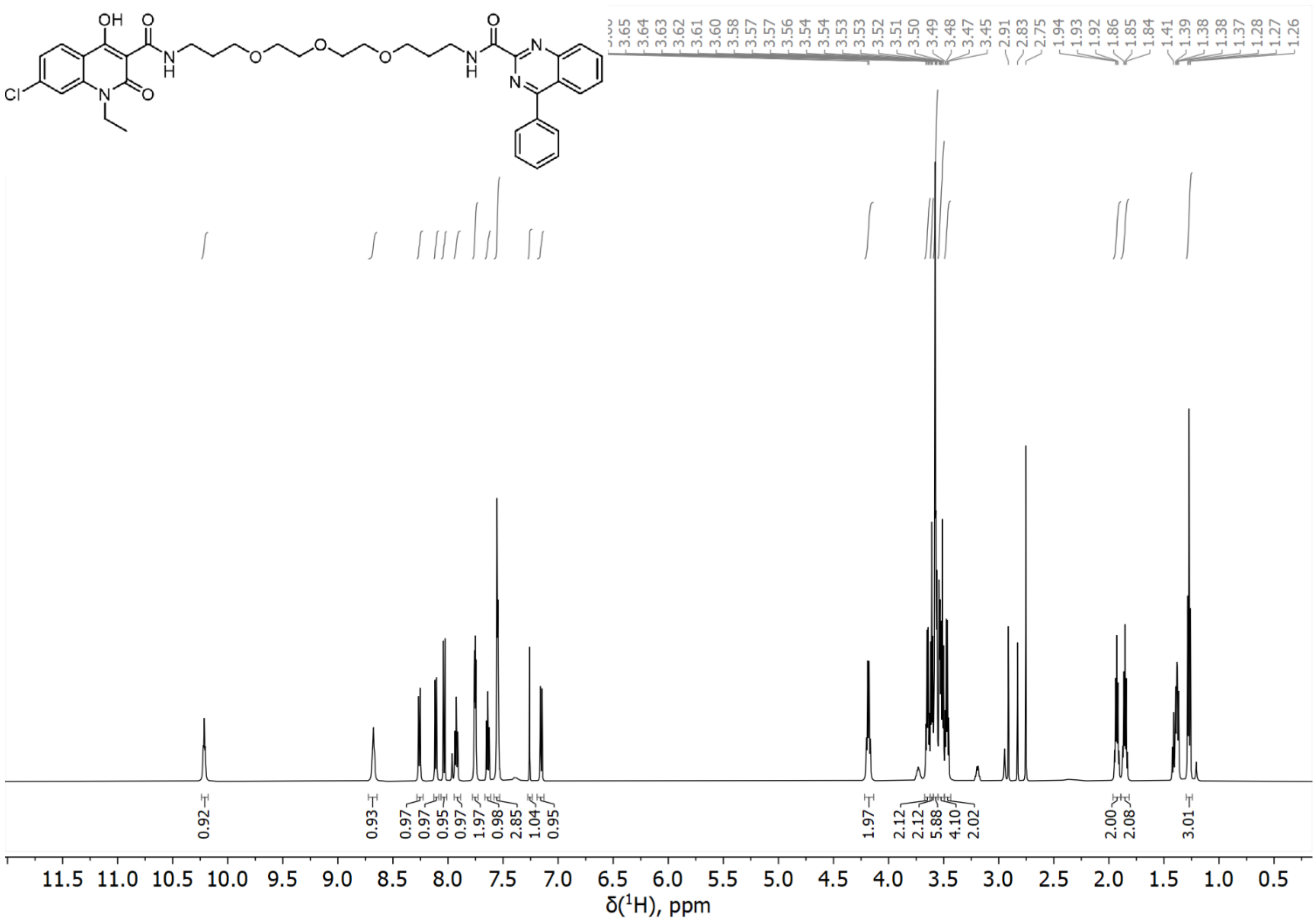

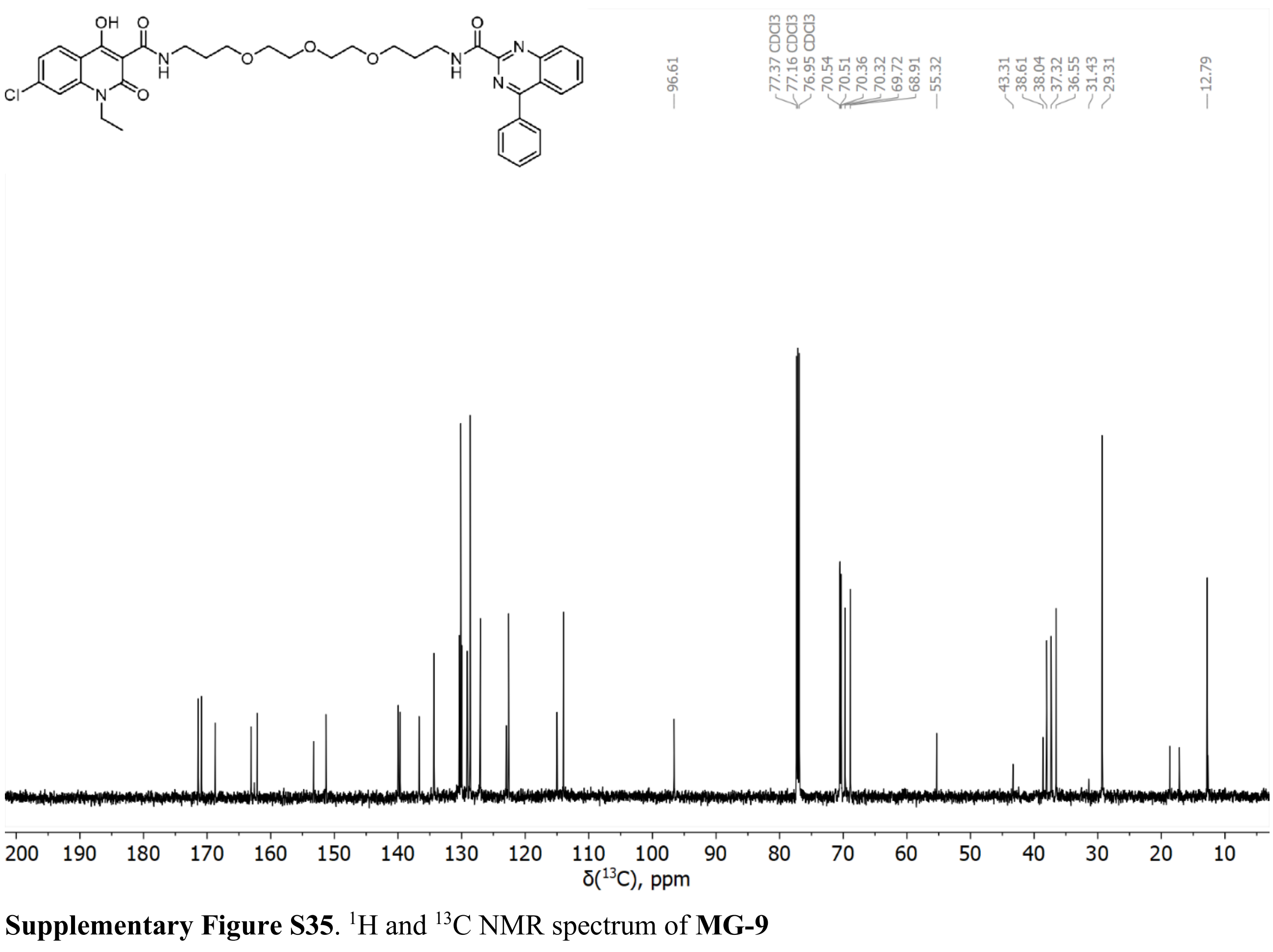

Supplementary Figure S35. ^{1}H and ^{13}C NMR spectrum of **MG-9**

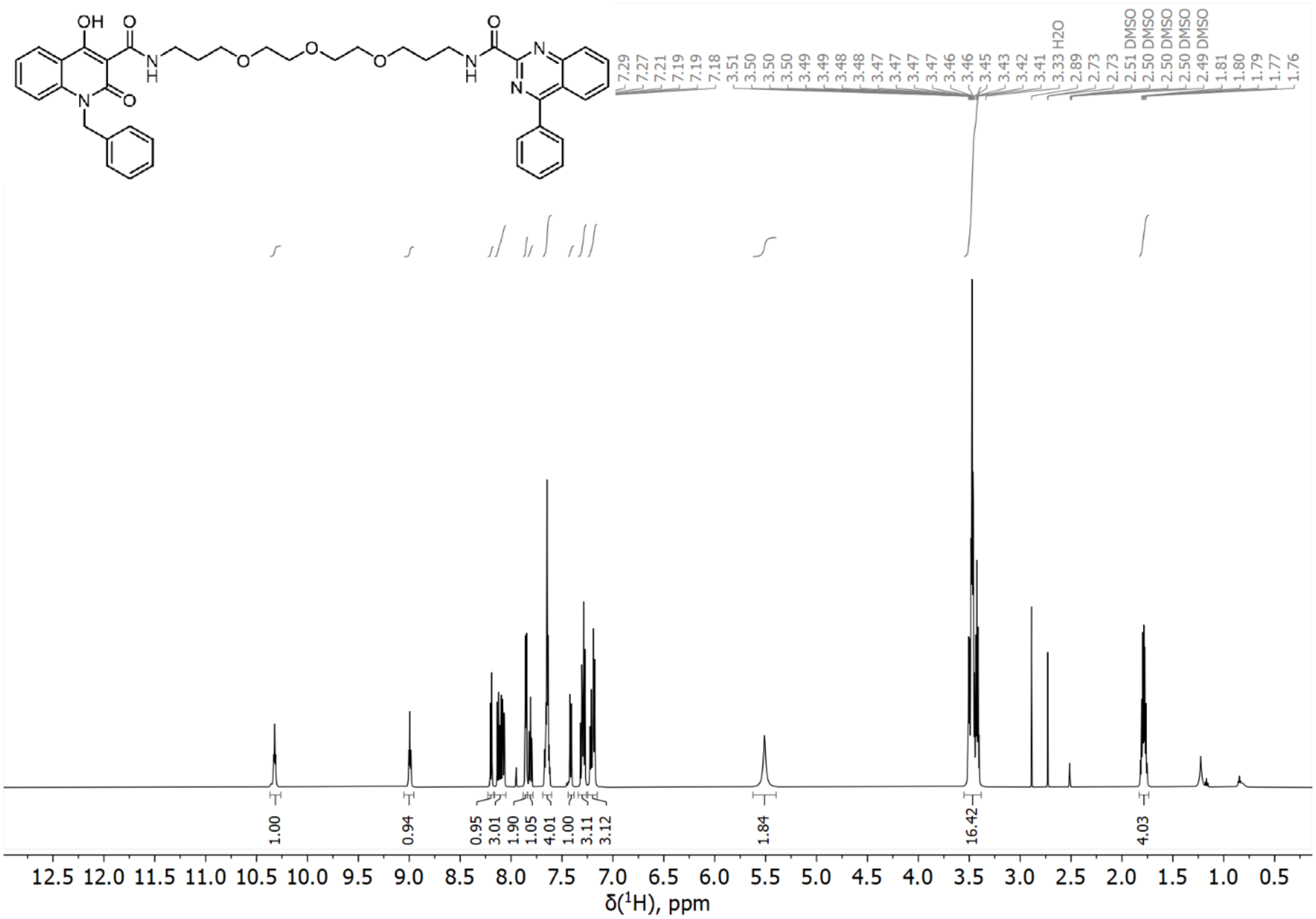

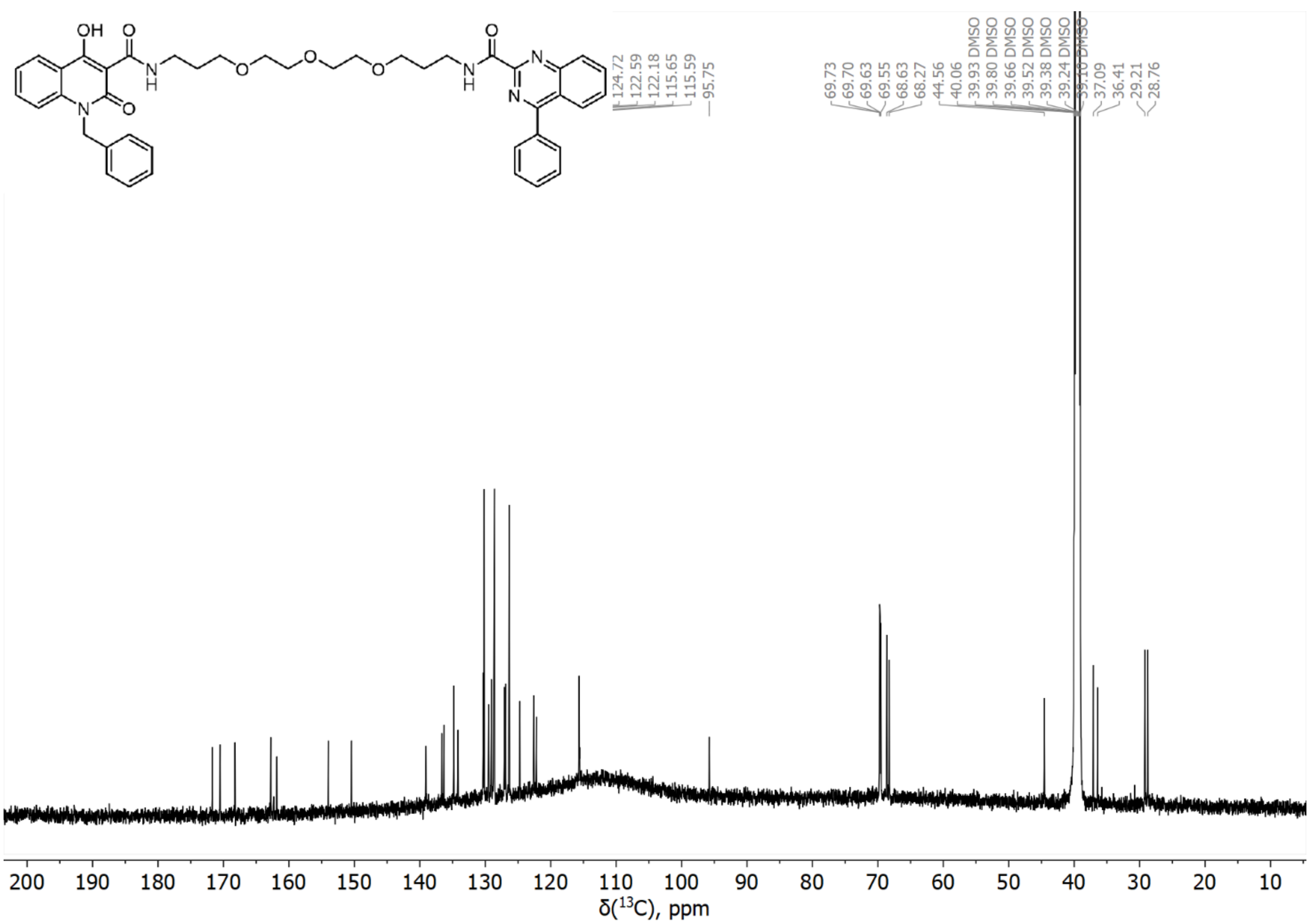

Supplementary Figure S36. ^{1}H and ^{13}C NMR spectrum of **MG-10**

References

1. E. A. Guseva, P. N. Kamzeeva, S. Y. Sokolskaya, et al., “Modified (2′-Deoxy)Adenosines Activate Autophagy Primarily through AMPK/ULK1-Dependent Pathway,” *Bioorganic & Medicinal Chemistry Letters 113* (2024): 129980, https://doi.org/10.1016/j.bmcl.2024.129980.

2. E. A. Guseva, A. G. Tereshchenkov, P. N. Kamzeeva, et al., “AMPK-Specific Autophagy Activator Based on 1,3-Diaza-2-Oxophenoxazine,” *Bioorganic Chemistry 162* (2025): 108588, https://doi.org/10.1016/j.bioorg.2025.108588.

3. K. Morita, Y. Hama, T. Izume, et al., “Genome-Wide CRISPR Screen Identifies TMEM41B as a Gene Required for Autophagosome Formation,” *Journal of Cell Biology 217*, no. 11 (2018): 3817–3828, https://doi.org/10.1083/jcb.201804132.

4. J. R. Liang, E. Lingeman, S. Ahmed, and J. E. Corn, “Atlastins Remodel the Endoplasmic Reticulum for Selective Autophagy,” *Journal of Cell Biology 217*, no. 10 (2018): 3354–3367, https://doi.org/10.1083/jcb.201804185.

5. K. R. Martin, S. L. Celano, A. R. Solitro, et al., “A Potent and Selective ULK1 Inhibitor Suppresses Autophagy and Sensitizes Cancer Cells to Nutrient Stress,” *iScience 8* (2018): 74–84, https://doi.org/10.1016/j.isci.2018.09.012.

6. O. A. Averina, O. A. Permyakov, M. A. Emelianova, et al., “Kidney-Related Function of Mitochondrial Protein Mitoregulin,” *International Journal of Molecular Sciences 24*, no. 10 (2023): 9106, https://doi.org/10.3390/ijms24109106.

7. J. Schindelin, I. Arganda-Carreras, E. Frise, et al., “Fiji: An Open-Source Platform for Biological-Image Analysis,” *Nature Methods 9*, no. 7 (2012): 676–682, https://doi.org/10.1038/nmeth.2019.

8. M. H. Fabricius, L. K. Wilms, J. Larsen, P. L. Pedersen, S. Anthonsen, and J. Kvetny, “Measure of Expression of Mitochondrial Related Genes in Human Mononuclear Blood Cells, Adipose White Tissue and Smooth Muscle Cells,” *Clinica Chimica Acta 411*, nos. 9–10 (2010): 749–753, https://doi.org/10.1016/j.cca.2010.02.005.

9. C. Northam, and C. M. R. LeMoine, “Metabolic Regulation by the PGC-1α and PGC-1β Coactivators in Larval Zebrafish (Danio Rerio),” *Comparative Biochemistry and Physiology Part A: Molecular & Integrative Physiology 234* (2019): 60–67, https://doi.org/10.1016/j.cbpa.2019.04.011.

10. M. Porta-de-la-Riva, L. Fontrodona, A. Villanueva, and J. Cerón, “Basic Caenorhabditis Elegans Methods: Synchronization and Observation.,” *Journal of visualized experiments : JoVE* no. 64 (2012): e4019–e4019, https://doi.org/10.3791/4019.

11. Test No. 420: Acute Oral Toxicity - Fixed Dose Procedure, OECD 2002, https://doi.org/10.1787/9789264070943-en.

12. O. Wasel, and J. L. Freeman, “Chemical and Genetic Zebrafish Models to Define Mechanisms of and Treatments for Dopaminergic Neurodegeneration,” *International Journal of Molecular Sciences 21*, no. 17 (2020): 5981, https://doi.org/10.3390/ijms21175981.

13. J. Schweitzer, and W. Driever, “Development of the Dopamine Systems in Zebrafish,” 2009, pp. 1–14, https://doi.org/10.1007/978-1-4419-0322-8_1.

14. A. M. Waterhouse, J. B. Procter, D. M. A. Martin, M. Clamp, and G. J. Barton, “Jalview Version 2—a Multiple Sequence Alignment Editor and Analysis Workbench,” *Bioinformatics 25*, no. 9 (2009): 1189–1191, https://doi.org/10.1093/bioinformatics/btp033.

15. R. C. Edgar, “MUSCLE: A Multiple Sequence Alignment Method with Reduced Time and Space Complexity,” *BMC Bioinformatics 5*, no. 1 (2004): 113, https://doi.org/10.1186/1471-2105-5-113.

16. A. Soriano, R. Ventura, A. Molero, et al., “Adenosine A $_{2A}$ Receptor-Antagonist/Dopamine D $_2$ Receptor-Agonist Bivalent Ligands as Pharmacological Tools to Detect A $_{2A}$ -D $_2$ Receptor Heteromers,” *Journal of Medicinal Chemistry 52*, no. 18 (2009): 5590–5602, https://doi.org/10.1021/jm900298c.

17. R. Y. Utomo, S. Okada, A. Sumiyoshi, I. Aoki, and H. Nakamura, “Development of an MRI Contrast Agent for Both Detection and Inhibition of the Amyloid-β Fibrillation Process,” *RSC Advances 12*, no. 8 (2022): 5027–5030, https://doi.org/10.1039/D2RA00614F.

18. J. Lu, L. Lei, Y. Huan, et al., “Design, Synthesis, and Activity Evaluation of GK/PPARγ Dual-Target-Directed Ligands as Hypoglycemic Agents,” *ChemMedChem 9*, no. 5 (2014): 922–927, https://doi.org/10.1002/cmdc.201400009.

19. S. Castellano, S. Taliani, C. Milite, et al., “Synthesis and Biological Evaluation of 4-Phenylquinazoline-2-Carboxamides Designed as a Novel Class of Potent Ligands of the Translocator Protein,” *Journal of Medicinal Chemistry 55*, no. 9 (2012): 4506–4510, https://doi.org/10.1021/jm201703k.

20. I. Kostopoulou, A. Tzani, K. Chronaki, et al., “Novel Multi-Target Agents Based on the Privileged Structure of 4-Hydroxy-2-Quinolinone,” *Molecules 29*, no. 1 (2023): 190, https://doi.org/10.3390/molecules29010190.

21. V. Palomo, D. I. Perez, C. Roca, et al., “Subtly Modulating Glycogen Synthase Kinase 3 β: Allosteric Inhibitor Development and Their Potential for the Treatment of Chronic Diseases,” *Journal of Medicinal Chemistry 60*, no. 12 (2017): 4983–5001, https://doi.org/10.1021/acs.jmedchem.7b00395.

22. G. L. Beutner, J. T. Kuethe, and N. Yasuda, “A Practical Method for Preparation of 4-Hydroxyquinolinone Esters,” *The Journal of Organic Chemistry 72*, no. 18 (2007): 7058–7061, https://doi.org/10.1021/jo071200x.
